# Stream microbial communities and ecosystem functioning show complex responses to multiple stressors in wastewater

**DOI:** 10.1111/gcb.15302

**Published:** 2020-09-03

**Authors:** Francis J. Burdon, Yaohui Bai, Marta Reyes, Manu Tamminen, Philipp Staudacher, Simon Mangold, Heinz Singer, Katja Räsänen, Adriano Joss, Scott D. Tiegs, Jukka Jokela, Rik I. L. Eggen, Christian Stamm

**Affiliations:** ^1^ Eawag Swiss Federal Institute of Aquatic Science and Technology Dübendorf Switzerland; ^2^ Department of Aquatic Sciences and Assessment Swedish University of Agricultural Sciences Uppsala Sweden; ^3^ Research Center for Eco‐Environmental Sciences Chinese Academy of Sciences Beijing People's Republic of China; ^4^ Institute of Integrative Biology ETH Zürich Zürich Switzerland; ^5^ Department of Biological Sciences Oakland University Rochester MI USA; ^6^ Institute of Biogeochemistry and Pollutant Dynamics ETH Zürich Zürich Switzerland; ^7^ Present address: Department of Biology University of Turku Turku Finland; ^8^ Present address: Agroscope Reckenholzstrasse 191 Zurich 8046 Switzerland

**Keywords:** biodiversity, carbon processing, cotton‐strip assay, micropollutants, next‐generation sequencing, nutrients, temperature, warming

## Abstract

Multiple anthropogenic drivers are changing ecosystems globally, with a disproportionate and intensifying impact on freshwater habitats. A major impact of urbanization are inputs from wastewater treatment plants (WWTPs). Initially designed to reduce eutrophication and improve water quality, WWTPs increasingly release a multitude of micropollutants (MPs; i.e., synthetic chemicals) and microbes (including antibiotic‐resistant bacteria) to receiving environments. This pollution may have pervasive impacts on biodiversity and ecosystem services. Viewed through multiple lenses of macroecological and ecotoxicological theory, we combined field, flume, and laboratory experiments to determine the effects of wastewater (WW) on microbial communities and organic‐matter processing using a standardized decomposition assay. First, we conducted a mensurative experiment sampling 60 locations above and below WWTP discharges in 20 Swiss streams. Microbial respiration and decomposition rates were positively influenced by WW inputs via warming and nutrient enrichment, but with a notable exception: WW decreased the activation energy of decomposition, indicating a “slowing” of this fundamental ecosystem process in response to temperature. Second, next‐generation sequencing indicated that microbial community structure below WWTPs was altered, with significant compositional turnover, reduced richness, and evidence of negative MP influences. Third, a series of flume experiments confirmed that although diluted WW generally has positive influences on microbial‐mediated processes, the negative effects of MPs are “masked” by nutrient enrichment. Finally, transplant experiments suggested that WW‐borne microbes enhance decomposition rates. Taken together, our results affirm the multiple stressor paradigm by showing that different aspects of WW (warming, nutrients, microbes, and MPs) jointly influence ecosystem functioning in complex ways. Increased respiration rates below WWTPs potentially generate ecosystem “disservices” via greater carbon evasion from streams and rivers. However, toxic MP effects may fundamentally alter ecological scaling relationships, indicating the need for a rapprochement between ecotoxicological and macroecological perspectives.

## Graphical abstract

Global change assessments have typically ignored synthetic chemical pollution, despite the rapid increase of pharmaceuticals, pesticides, and industrial chemicals in the environment. Inputs of treated municipal wastewater are a major source of synthetic chemicals in receiving aquatic ecosystems. We found that microbial activity and decomposition rates were positively influenced by the presence of treated effluent via warming and nutrient enrichment, despite negative effects of synthetic chemicals, leading to increased respiration and estimated carbon evasion in streams and rivers.

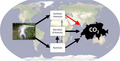

## 1 INTRODUCTION

Ecosystems are increasingly impacted by multiple anthropogenic pressures at a global scale, with a disproportionate and intensifying effect on freshwaters (Harrison et al., 2018; Reid et al., 2019). In particular, human alterations of material cycles (e.g., eutrophication) may profoundly affect freshwater biodiversity and ecosystem functioning at continental scales (Woodward et al., 2012). However, while the deterioration of freshwater ecosystems is evident, discerning causal pathways of environmental change remains difficult (Burdon McIntosh, & Harding, 2013, 2020). This challenge reflects the natural complexity underpinning ecosystems but also because multiple‐stressor interactions can lead to “ecological surprises” via antagonisms and synergisms (Jackson, Loewen, Vinebrooke, & Chimimba, 2016). Urban human populations are one pervasive source of ecological degradation with multifarious stressor pathways (Walsh et al., 2005). Initially designed to mitigate anthropogenic eutrophication (Vaughan & Ormerod, 2012), municipal wastewater treatment plants (WWTPs) are human‐made aquatic ecosystems that continue to influence receiving environments as urban populations grow and developing countries increase their use (Burdon et al., 2016; Schwarzenbach, Egli, Hofstetter, von Gunten, & Wehrli, 2010). These impacts include hydrological changes, altered thermal dynamics, and inputs of chemicals (e.g., nutrients) and microbes (Hamdhani, Eppehimer, & Bogan, 2020; Stamm et al., 2016).

Wastewater (WW) treatment plants are designed to perform key ecosystem services that include the removal of pathogens, degradable organic compounds, and nutrients from the WW effluent (Eggen, Hollender, Joss, Schärer, & Stamm, 2014). To achieve these goals, WWTPs typically make use of biological processes (i.e., “secondary” treatment) that are performed by aquatic microorganisms (e.g., bacteria) within a managed aerobic habitat (Shammas & Wang, 2010). Even with these beneficial treatment effects, WWTPs can still release putatively harmful materials and organisms because existing infrastructure are not optimized for their removal. WW‐borne contaminants include antibiotic‐resistant bacteria (typically from hospitals; Czekalski, Berthold, Caucci, Egli, & Burgmann, 2012), microplastics (Kay, Hiscoe, Moberley, Bajic, & McKenna, 2018), and synthetic chemicals (Stamm et al., 2016). The latter is of particular concern, because despite a steady rise in the manufacture and release of synthetic chemicals to the environment globally, research on the ecological effects of “micropollutants” is severely lacking (Bernhardt, Rosi, & Gessner, 2017; Wang, Walker, Muir, & Nagatani‐Yoshida, 2020).

Micropollutants (MPs) typically consist a wide array of substances including pharmaceuticals, personal care products, pesticides, and industrial chemicals (Schwarzenbach et al., 2006). These are usually present at very low concentrations (ng to μg/L), but often have high potency due to their bioactive properties (Stamm et al., 2016). MPs can enter aquatic environments from agricultural and urban runoff (Wittmer et al., 2010), although for many compounds the main source are WWTP discharges (Luo et al., 2014). Consequently, MPs impact freshwater ecosystems at the continental scale (Malaj et al., 2014) with potential negative effects across multiple levels of biological organization (i.e., ranging from the cellular to the ecosystem level; Halstead et al., 2014; Ren, Lee, Han, & Kim, 2009). For instance, MPs can have adverse effects on detritivorous invertebrates at the individual level, leading to lower rates of consumption which may upscale to ecosystem‐level impacts on organic‐matter processing (Bundschuh, Pierstorf, Schreiber, & Schulz, 2011).

Organic‐matter decomposition is a fundamental ecosystem process that contributes to the global carbon cycle by regulating local carbon stocks and fluxes, thus influencing concentrations of atmospheric CO_2_ and CH_4_ (Battin et al., 2009). This ecosystem process also affects food‐web dynamics and ecosystem functioning in a wide range of habitats, including headwater streams (Moore et al., 2004; Tank, Rosi‐Marshall, Griffiths, Entrekin, & Stephen, 2010). In stream ecosystems, organic matter is broken down by physical and biological processes that include microbial conditioning (i.e., mineralizing leaf litter and rendering it more palatable to higher consumers) and detrital consumption by invertebrates (Hieber & Gessner, 2002). Consequently, anthropogenic stressors that affect key food‐web compartments can influence breakdown rates, making decomposition assays a powerful functional indicator for measuring environmental change (Gessner & Chauvet, 2002).

Microbes can play a compensatory role in organic‐matter processing when stream invertebrates are impacted by pollution (Pascoal, Cássio, Marcotegui, Sanz, & Gomes, 2005). In particular, faster decomposition rates influenced by inputs of WW (e.g., Spänhoff et al., 2006) could in part result from positive effects of warming and nutrient enrichment that offset the impacts of toxic pollutants present in the effluent (Burdon et al., 2016). However, although the relationship between microbial community structure and functioning may be environmentally contingent (Feckler et al., 2018), biodiversity‐ecosystem functioning (BEF) theory suggests that positive diversity effects due to complementarity (e.g., resource partitioning and facilitation) or selection (i.e., an increased probability of species with strong effects) mechanisms should be lost with increasing pollution (Cardinale, 2011; Gessner et al., 2010). Several studies have identified positive BEF relationships for microbial community stability (Awasthi, Singh, Soni, Singh, & Kalra, 2014), MP degradation (Johnson et al., 2015), and decomposition (Evans et al., 2017), indicating that for a range of functions microbial community performance improves with increasing species richness. Thus, determining how freshwater microbes and organic‐matter decomposition respond to widespread human pressures such as WWTPs is fundamentally important to understanding environmental change (Cavicchioli et al., 2019).

To assess how WW‐borne stressors influence stream ecosystem functioning, we used standardized cotton‐strip assays (CSA) in experiments involving a mensurative field survey, artificial channels (flumes), and laboratory microcosms. The CSA has been used to demonstrate how ecosystem processes change across global environmental gradients, making it an important functional indicator of microbial‐mediated decomposition (Tiegs et al., 2019). The assay is sensitive to human impacts in freshwater ecosystems including metal pollution (Costello & Burton, 2014), warming (Griffiths & Tiegs, 2016), lake‐shoreline “hardening” (Wensink & Tiegs, 2016), and stream acidification (Colas et al., 2019). In our study, we sought to better understand the growing threat of MPs to microbial communities and ecosystem functioning using the CSA. Given the inherent complexity of receiving stream ecosystems and the multifarious composition of WW effluent, we made the following predictions:

1. Consistent with BEF theory (Cardinale, 2011; Gessner et al., 2010), decomposition rates downstream of the WW input could be *lower* if there are negative effects of WW‐borne MPs on microbial diversity (Drury, Rosi‐Marshall, & Kelly, 2013), or *higher* if microbial community diversity was greater (due to a combining of natural stream and WW‐borne microorganisms; Wakelin, Colloff, & Kookana, 2008). Alternatively, and potentially in conflict with BEF theory, decomposition rates might be conserved or *higher* downstream even if there was a decrease in diversity (Drury et al., 2013; Feckler et al., 2018). This alternate hypothesis is grounded in the pollution‐induced community tolerance (PICT) paradigm, where an increase in tolerance to chemical pollutants due to environmental filtering of sensitive species is predicted (i.e., species turnover), thus helping to preserve process rates due to functional redundancies (Clements & Rohr, 2009).
2. Multiple‐stressor theory (Jackson et al., 2016) suggests that different constituents of WW might exert opposing effects. On the one hand, the negative effects of MPs on decomposition could be “masked” by the positive effects of warming and nutrient enrichment on microbial community activity. Alternatively, the presence of MPs could fundamentally alter the performance of microbes, thus affecting the temperature‐dependent scaling relationship predicted by the metabolic theory of ecology (MTE; Brown, Gillooly, Allen, Savage, & West, 2004).
3. Differing concentrations of chemicals targeting key biotic compartments could lead to variation in ecosystem responses. In particular, we expected that MPs with fungicidal or bactericidal properties would be more associated with reduced functioning than other synthetic compounds because these chemicals target microbes contributing to decomposition (Stamm et al., 2016; Zubrod et al., 2019).

We used the CSA as a model functional indicator to enable comparisons of different processes affecting decomposition across field, flumes, and laboratory experiments, thus providing new insights for understanding how the complexity of WW‐borne stressors influence aquatic ecosystems. We argue that reconciling macroecological and ecotoxicological perspectives would form the basis for a more predictive, transdisciplinary approach to understanding interactions between environmental stressors, biodiversity change, and ecosystem functioning.

## 2 MATERIALS AND METHODS

### 2.1 Site selection and study design

In all, 20 sites across Switzerland were selected to investigate WWTP impacts on receiving stream ecosystems (Burdon et al., 2016; Stamm et al., 2016). Briefly, streams used had no other WWTPs upstream, treated WW >20% of total discharge (*Q*_347_), and catchment land‐uses <21% urban and <10% orcharding (e.g., vineyards) by area. At each study site, we designated one downstream sampling location (D), and two upstream sampling locations (U1, U2; Stamm et al., 2016). Sites had comparable stream morphology, riparian land use and vegetation types above and below WW discharges. In all, 12 sites were used in 2013 and 8 in 2014. For further site details, see Appendix A.

### 2.2 Water quality

We characterized water quality over 12 months at upstream and downstream locations by collecting grab samples during base flow conditions. For the 2013 sites, we took monthly grab samples between March 2013 and February 2014. For the 2014 sites, we sampled bi‐monthly (from March 2014 to January 2015). We analyzed all samples for 20 general water quality parameters (Table SC1). We analyzed organic MPs in two samples (June 2013 and February 2014) for the 2013 sites, and MPs and heavy metals (HMs) in all six samples for the 2014 sites (Munz et al., 2016). Toxic units (TUs) were calculated from organic MPs and HMs following Munz et al. (2016). We calculated WW quantity as the proportion of treated effluent in the receiving stream (Burdon et al., 2019). For more details, see Appendix C.

### 2.3 Cotton‐strip assay

We used the CSA following Tiegs, Clapcott, Griffiths, and Boulton (2013). For the field survey, we attached four strips, evenly spaced apart (≈20 cm) to a nylon cord anchored to the streambed in shallow run‐riffle habitats near the bank. We allocated eight cotton strips (CSs) on two cords to each sampling location (D, U1, U2) at 12 sites in 2013 (4 November–18 December) and eight sites in 2014 (5 November–2 December). We incubated CSs for 14 days in 2013 (121 ± 19 degree‐days, mean ± 1 *SD*) with one exception (Messen, 19 days). In 2014, we incubated strips for 20 days (193 ± 17 degree‐days).

We performed an in situ respiration assay on the CSs at the time of collection following Tiegs et al. (2013). Dissolved oxygen (DO; mg/L) was recorded at the beginning and end of the assay using a handheld probe (Hach HQ40d). We then immersed the strips in 100% ethanol (10 s) before oven drying (48°C for 72 hr) and weighing to the nearest 0.001 mg. We calculated the oxygen consumption of microbes on each CS, *R*_CS_ (i.e., respiration) as:

(1) $$R_{\text{CS}}=\left[\left({\text{DO}}_{S_{0}}‐{\text{DO}}_{{\text{CS}}_{t}}\right)‐\left({\text{DO}}_{S_{0}}‐{\text{DO}}_{C_{t}}\right)\right]\times \frac{V_{H_{2}O}}{(m_{\text{CS}})t},$$

where ${\text{DO}}_{S_{0}}$ is the DO concentration in the stream water at the start of the incubation, ${\text{DO}}_{{\text{CS}}_{t}}$ is the DO concentration in the cotton‐strip chambers at the end of the incubation period *t*, ${\text{DO}}_{C_{t}}$ is the DO concentration in the control (i.e., blank) chambers at *t*, $V_{H_{2}O}$ is the volume of water in the respiration chamber, and *m*_CS_ is the cotton‐strip mass.

We converted oxygen consumption to CO_2_ production (mg C hr^−1^ g^−1^ DM) using the empirically‐derived respiratory quotient in Berggren, Lapierre, and del Giorgio (2012). We then used our respiration data to estimate carbon evasion at study locations and in the flumes experiments using a hydrogeomorphic scaling relationship (Raymond et al., 2012). Finally, we used two approaches to scale‐up changes in carbon evasion to estimate the increase in Swiss carbon emissions due to inputs of WW in small‐ to medium‐sized stream and rivers. The first approach used global estimates for lotic carbon fluxes (Raymond et al., 2013) and surface area (Allen & Pavelsky, 2018) in combination with the median relative change in evasion at downstream locations. The second approach relied on the median absolute areal change in carbon evasion. Both approaches accounted for the surface area of small–medium streams and rivers (Strahler stream order 1–6; i.e., stream sizes based on a hierarchy of tributaries) affected by WW inputs in Switzerland. For more details on these methods, see Appendix C.1.

We used a tensiometer (Mark‐10, MG100) to estimate cotton‐strip tensile strength (TS) loss. After oven‐drying, individual strips were pulled at a fixed rate of 2 cm/min and maximum TS recorded. We used TS to estimate the cotton‐breakdown rate coefficient *k* (Equation 2):

(2) $$k_{D}=\frac{‐\ln\left(\frac{{\text{TS}}_{t}}{{\text{TS}}_{0}}\right)}{t},$$

where TS*_t_* is the maximum tensile strength recorded for each of the strips incubated in the field, TS_0_ is the mean tensile strength of 10 strips that were not incubated in the field (but sterilized with ethanol, dried, and stored in a desiccator), and *t* is the incubation period (days). We summed the average daily water temperatures over each incubation period to calculate temperature‐days (i.e., the temperature‐days accumulated from Day 1 to the retrieval day) for *t* (Benfield, 2007); an approach that accounts for temperature effects. Finally, to supplement our main indicators of microbial activity (i.e., cotton‐strip respiration and TS loss), we calculated cotton mass‐loss rates using the general form of Equation (2), replacing TS_0_ with mean initial mass (*n* = 10) and TS*_t_* with final mass following incubation in the field.

We estimated the activation energies of two ecosystem functions (EFs) using the CSA: (a) community respiration and (b) decomposition (i.e., TS loss [*k*_D_]) from a linearized Arrhenius function (Equation 3):

(3) $$\ln\text{EF(}T)=‐E_{a}\left(\frac{1}{k_{B}T}‐\frac{1}{k_{B}T_{\bar{x}}}\right)+\ln\left[\text{EF(}T_{\bar{x}})\right],$$

where EF(*T*) is the rate of the respective ecosystem functions at absolute temperature *T* (Kelvin), *k*_B_ is the Boltzmann constant (8.6 × 10^−5^ eV/K), and *E*_a_ is the activation energy. Following Perkins et al. (2012), we express the natural logarithm (ln) of the EF as a function of standardized temperature ($1/k_{B}T‐1/k_{B}T_{\bar{x}}$) which centers the inverse temperature data around zero, to make the intercept of the model ($\ln\left[\text{EF}\left(T_{\bar{x}}\right)\right]$) equal to the EF rate at standardized temperature, $T_{\bar{x}}$ (here $T_{\bar{x}}$ = 6.748°C = 279.9 K). In Equation (3), which is similar in form to those derived from the MTE (e.g., Perkins et al., 2012; Yvon‐Durocher, Jones, Trimmer, Woodward, & Montoya, 2010), $\ln\left[\text{EF}\left(T_{\bar{x}}\right)\right]$ is hypothesized to be determined by the total mass‐corrected biomass of organisms in the “ecosystem” (Allen, Gillooly, & Brown, 2005)—here the CS. To ensure consistency with the above approach, we expressed the cumulative degree‐days used to temperature correct tensile and mass‐loss rates (*k*_D_) as (1/*k*_B_T), and hence are described as “temperature‐days.”

### 2.4 Microbial communities

We took a 10‐mm subsample of cotton material from individual strips retrieved at 10 of the field sites in 2013 for the analysis of microbial communities (Table SA1). In the field, we pooled two cotton‐strip sections for one sample in a sterile plastic microtube stored on ice to obtain triplicate samples from each sampling location. We later (<12 hr) stored samples at −80°C prior to further analysis. We used one cotton‐strip sample per sampling location (D, U1) at 10 streams for DNA extraction using a PowerBiofilm DNA Isolation Kit (MO BIO Laboratories). We used next‐generation sequencing (NGS) to profile bacterial and fungal communities on the cotton strips (CSs). We amplified 16S rRNA and ITS1 genes from cotton‐strip DNA samples by polymerase chain reaction (PCR) using barcoded primers in triplicate. The 16S rRNA gene was amplified using primers 515F (5′‐GTGCCAGCMGCCGCGG‐3′) and 907R (5′‐CCGTCAATTCMTTTRAGTT‐3′). The ITS1 gene was amplified using primers ITS1‐F (5′‐CTTGGTCATTTAGAGGAAGTAA‐3′) and ITS2‐R (5′‐GCTGCGTTCTTCATCGATGC‐3′). See Table SC3 for thermal programs used. We purified PCR products using an AxyPrep DNA Gel Extraction Kit (Axygen). We measured the DNA concentration of the purified PCR products using the QuantiFluor™‐ST dsDNA System (Promega). Prior to sequencing, we pooled the amplicons from triplicate PCR products in equal amounts based on concentration. BGI performed the sequencing using the Illumina MiSeq sequencing platform. We initially filtered sequencing reads by removing low‐quality reads using the “fastq_filter” functionality of “usearch” (v. 8.1). We clustered the filtered sequences into operational taxonomic unit (OTUs) using the “fastx_uniques” and “cluster_otus” functionality of usearch. Finally, we created OTU tables using the “usearch_global” functionality of usearch with 97% sequence similarity as the OTU threshold. We determined the taxonomy using the “sintax” functionality of usearch; the “RDP” 16S database (v. 16) was used for the 16S rRNA OTUs and the “uchime” reference dataset (June 28, 2016) used for the ITS1 sequences. We performed OTU processing with R (v. 3.6.1) using “Tidyverse” (v. 1.2.1) and “phyloseq” (v. 1.28) and normalized the read counts by dividing each OTU read count by the total sample read count (McMurdie & Holmes, 2013). We compared sites and identified indicator taxa using “DESeq2” (v. 1.24; Love, Huber, & Anders, 2014); for ITS1 gene data, we compared occupancy patterns of potential indicator taxa. Unix commands and R code used for sequence and OTU processing are available at github.com/manutamminen/ecoimpact_cotton_strip_analyses.

### 2.5 Flumes experiments

We developed a system of 16 flumes (Maiandros; see technical description in Appendix B) configured in four experimental “blocks” to test the effects of WW and associated contaminants on decomposition using the CSA. Four water‐mixing units enabled us to deliver four distinct water treatments randomly assigned to one channel in each block. We located Maiandros at a WWTP (ARA Bächwiss) beside the River Glatt in Kanton Zürich. This site enabled river water to be mixed with WWTP effluent or dosed (e.g., with MPs or nutrients).

The first experiment (Exp. 1) tested the response of the CSA to river water (control) and three dilutions of secondary‐treated WW (15%, 50%, and 85%). We incubated four CSs in each channel for 13 days (Table 1). We recorded respiration, TS loss, and mass loss following the methods described above. The second and third experiments (Exp. 2, Exp. 3; Table 1) manipulated water quality by continuously dosing pre‐defined levels of MPs and nutrients to the river water (Table 1). We used these experiments to test the prediction that WW‐borne nutrients “mask” the negative effects of MPs. We used chemical mixtures for the two “dosing” experiments with compounds and concentrations described in Stamm et al. (2016) and listed in Table SC9. We continuously dosed at a rate of 0.65 ml/min for the entire experiment duration(s). Exp. 2 included a MP, a nutrient, and a MP + nutrient treatment. We incubated four CSs in each channel for 29 days (Table 1). In Exp. 3, we modified the chemical mixture to reduce the solvent (methanol) necessary to keep all MPs dissolved in the stock solution and substituted a technical control for the nutrient‐only treatment used in Exp. 2. For Exp. 3, we incubated four CSs in each channel for 35 days (Table 1). We recorded respiration, TS loss, and mass loss as described above.

**TABLE 1:** Description of the Maiandros flumes experiments conducted at ARA Bachwis, Fällanden, Switzerland using Glatt river water

Experiment	Treatment abbreviation	Treatment	Start	End	Results
Exp. 1	0% WW	River water (control)	August 6–7, 2014	August 19–20, 2014	Figure 4a,b
	15% WW	River mixed with treated WW (15%)			
	50% WW	River + WW (50%)			
	85% WW	River + WW (85%)			
Exp. 2	River	River water (control)	October 21–22, 2014	November 18–19, 2014	Figure SD16
	Nutrients	River dosed with N and P			
	MPs	River + MP‐mix.1			
	MPs + Nutrients	River + MP‐mix.1 + N and P			
Exp. 3	River	River water (control)	April 14–15, 2015	May 19–20, 2015	Figure 4c,d
	Control	River dosed with methanol (technical control)			
	MPs	River + MP‐mix.2			
	MPs + Nutrients	River + MP‐mix.2 + N and P			
Exp. 4	River	River water (control)	October 21, 2015	November 10, 2015	Figure 4e
	50% WW	River water mixed with treated WW (50%)			

We used Exp. 4 (Table 1) to test the hypothesis that inoculation with WW‐borne microbes leads to enhanced cotton decomposition (i.e., the inoculation hypothesis). In all, 18 CSs were inoculated in one of two treatments (river water, 50% river water + 50% WW) for 7 days (i.e., 36 strips in total). We then transferred 12 strips from each treatment to individual semi‐permeable membrane‐based mesocosms following Pomati and Nizzetto (2013). For the mesocosms, we used reconstituted cellulose dialysis membranes (Spectra/Por; Spectrum Europe) with a molecular weight cutoff of 3.5 kDa. These mesocosms were intended to prevent further microbial colonization of strips while maintaining realistic environmental conditions. We reciprocally transplanted six strips in mesocosms into the opposing treatment following inoculation. We kept another six strips in mesocosms in each inoculation treatment. The final six strips were left fully exposed to the water treatments in each inoculation treatment to assess mesocosm “cage‐effects.” We placed two strips per treatment combination in six channels (i.e., each water treatment paired in three experimental blocks). Following the transplants, we incubated CSs for a further 14 days at which point we ended the experiment because of degradation to the membranes in the diluted WW treatment. We recorded cotton‐strip TS and mass loss following the methods described above.

We encountered two complications with the flumes experiments. Exp. 1 and Exp. 2 experienced flow‐regulation issues, but we cannot detect any systematic biases in treatments because this problem equally affected all channels. Treatments in Exp. 2 were affected by the methanol concentrations used to keep MPs and nutrients in solution, so we report the results of this experiment in Appendix D. Full details for the flumes experiments are provided in Appendices C and D.

### 2.6 Laboratory experiment

We performed a reciprocal‐transplant laboratory experiment to further test the inoculation hypothesis. We inoculated 150 CSs with aquatic microbes at sampling locations above and below WW discharges at three study sites (Table SA1). After incubation in the stream for 7 days (July 3–10, 2014), we removed the CSs and placed them in stream‐water filled containers for transport to the laboratory. We reserved five strips from each sampling location at this point to baseline response measurements (i.e., TS and mass loss). After exposure in the streams, we incubated the CSs in the laboratory treatments for another 7 days (July 11–18, 2014). The 20 remaining strips from each of the six sampling locations were exposed to four different treatments with five replicate strips each. The four water treatments used were (a) downstream water filtered and UV irradiated (DS‐F), (b) untreated control downstream water (DS‐C), (c) upstream water filtered and UV irradiated (US‐F), and (d) untreated control upstream water (DS‐C). There was no exchange of water or CSs between streams, and we collected water from the six sampling locations at the time of retrieval. We filtered and irradiated water from each stream with ultraviolet light (i.e., treatments US‐F and DS‐F; see Appendix C.3.5 for details). Control water (i.e., US‐C and DS‐C) was left untreated. For the experiment, we filled 185 ml plastic cups with 125 ml of stream water from the respective water treatments. We then placed inoculated CSs (120 in total) in individual cups, grouped into six trays each with 20 cups. We submerged the CSs and each cup was aerated to create a gentle current and maintain DO concentrations (~8 mg/L). To replenish chemical concentrations, we exchanged another 125 ml of treatment water on Day 4 of the laboratory phase. After 7 days incubation in the laboratory, we recorded cotton‐strip respiration, TS loss, and mass loss following the methods described above.

### 2.7 Data analysis

#### 2.7.1 Communities

To test microbial (i.e., bacterial and fungal) responses using NGS data, we analyzed within sampling location (α) and among location (β) diversity. We estimated bacterial α‐diversity (richness and Shannon diversity) using a sample‐size‐based rarefaction and extrapolation (R/E) sampling curve approach extrapolated to 25,000 reads with the “iNEXT” (v. 2.0.20) R package (Hsieh, Ma, & Chao, 2016), following singleton‐correction for sequence data (Chiu & Chao, 2016). See Figure SC7 for R/E species richness curves. We also calculated evenness (Pielou's *J'*), dominance (Berger–Parker), and rareness (Fisher's alpha) from the singleton‐corrected 16S data using the “vegan” (v. 2.5‐6) R package (Oksanen et al., 2019). We only used OTU richness for the ITS1 data since this region is highly variable in length and copy number per cell meaning significant bias to some fungal taxa and groups, thus violating assumptions of rarefaction (Lindahl et al., 2013; Sota, Kagata, Ando, Utsumi, & Osono, 2014). We implemented non‐metric multidimensional scaling (NMDS) analysis on NGS data using the Bray–Curtis dissimilarity (Legendre & Gallagher, 2001). For these analyses, we used normalized counts (16S) as well as presence/absence (occupancy) data for both datasets (16S and ITS) provided by the “decostand” function in vegan. We used NMDS site scores (Axes 1−2) as response and predictor variables (see below). Differences in α and β‐diversity metrics were tested between sampling locations using linear mixed‐effect (LME) models with the random effect “Site.” We calculated mean standardized effect sizes with Hedge's correction using the “batch_calc_ES” function in the R package “SingleCaseES” (v. 0.4.3). We also tested LME models with a wider range of environmental predictors, using likelihood ratio tests to identify significant influence factors (Appendix C.4.1). Changes in community composition between sampling locations (U1, D) were tested using the vegan “adonis” (permutational multivariate analysis of variance; PERMANOVA) and “betadisper” (multivariate homogeneity of group dispersions) functions, respectively, with a strata term for site and permutation testing with 999 randomizations. We included upstream land use (% arable land) as a control factor in our PERMANOVA models (Table SD4). We used the “rda” and “varpart” functions to test the associations of Hellinger‐transformed microbial community data with (a) environmental predictors and (b) EFs (Appendix C.4.1.). We sought to disentangle the contribution of spatial turnover (species replacements) and nestedness (species losses) to β‐diversity patterns (Baselga, 2010). We calculated Sørenson's, Simpson's (turnover), and nestedness measures across and within sites using the “betapart” (v. 1.5.1) R package (Baselga & Orme, 2012).

#### 2.7.2 Ecosystems

We first used LME models to test WW impacts on ecosystem responses (Table 3). All models tested “Sampling Location” (D, U1, U2) as a fixed effect and included a random effect “Site.” Separate LME models for TS loss (*k*_D_) and respiration included a standardized temperature covariate (1/*k*_B_ *T* − 1/*k*_B_ $T_{\bar{x}}$) and its interaction with sampling location. Effect sizes (between U1 and D) were calculated using the R package “SingleCaseES.” We then used structural equation modeling (SEM) with the R package “piecewiseSEM” (v. 2.1.0) (Lefcheck, 2016) to test causal hypotheses about factors influencing temperature‐corrected processes rates. We tested SEMs for all sites (Appendix C.4.2) and the subset of 2013 sites (*n* = 10) where microbial NGS data were available. Possible predictors included two ultimate pressures (%WW and % arable land in the upstream catchment), four proximate stressors (concentrations of dissolved inorganic nitrogen [DIN] and soluble reactive phosphorus [SRP], and TUs of fungicides and non‐fungicides), and seven biodiversity metrics. These included OTU richness and site scores from NMDS (Axes 1−2) calculated from bacteria and fungi NGS data (see above). For bacteria, we also included Shannon diversity, rareness, dominance, and evenness. “Fungicide” TUs included fungicides, biocides (e.g., triclosan), and their metabolites (46 potential compounds in total). “Non‐fungicide” TUs included herbicides, insecticides, personal care and pharmaceutical products, and industrial chemicals (341 potential compounds in total). A random effect term was included for “Site.” We removed non‐significant paths if it improved model fit (indicated by lower Bayesian information criterion scores), and used Fisher's test of directed separation to help identify model adequacy and correlated error terms.

#### 2.7.3 Experiments

We analyzed results from the flumes experiments with LME models where “Treatments” were the fixed effect and “Channel Block” the random effect. Models testing respiration included mean channel temperature as a covariate. We analyzed results from the laboratory experiment with LME models where “Treatments” were the fixed effect and random effects “Site” and “Tray.” The respiration model included assay duration (time) as a covariate. Carbon efflux was strongly related to respiration in the experiments, so we only report results from the latter.

Prior to analysis, we log or logit‐transformed experimental data to improve normality and heteroscedasticity. We computed LME models with the R packages “lmer4” (v. 1.1‐21) or “blme” (v. 1.0‐4). For all LME, we identified post‐hoc differences using a least‐squares means approach (“lsmeans”) with multiplicity adjustments (Holm correction) obtained from the R package “lmerTest” (v. 3.1‐1). All analyses were conducted in R (v. 3.6.2; R Core Team, 2015). For further details on data analyses, see Appendix C.4.

## 3 RESULTS

### 3.1 Contaminants in wastewater

The concentration ranges for different MP groups varied within and across streams. The highest concentrations were observed for HMs with an average concentration per metal of ~1.0 μg/L upstream and 1.7 μg/L downstream of WWTPs (Table SD1). For organic MPs, we detected the highest concentrations for typical household chemicals including artificial sweeteners and corrosion inhibitors, followed by pharmaceuticals. These two compound classes increased the most downstream (10−14×). Concentration levels and the increase between sampling locations were much more moderate for the three pesticide classes (fungicides, herbicides, and insecticides). However, maximum concentrations for single compounds at specific locations exceeded 1 μg/L for all classes except insecticides (Table SD2), and fungicide concentrations more than doubled on average downstream at the 2013 sites. Estrogenic activity barely exceeded 1 ng/L estradiol equivalents even downstream of the WWTPs (Kienle et al., 2019). Nutrient levels also increased downstream, with DIN increasing from a median 3.1 mg/L upstream to 5.0 mg/L below the WW input (Table SD3). Likewise, SRP increased from a median 15.4 μg/L upstream to 49.1 μg/L downstream.

### 3.2 Wastewater impacts on microbial communities

**FIGURE 1:**
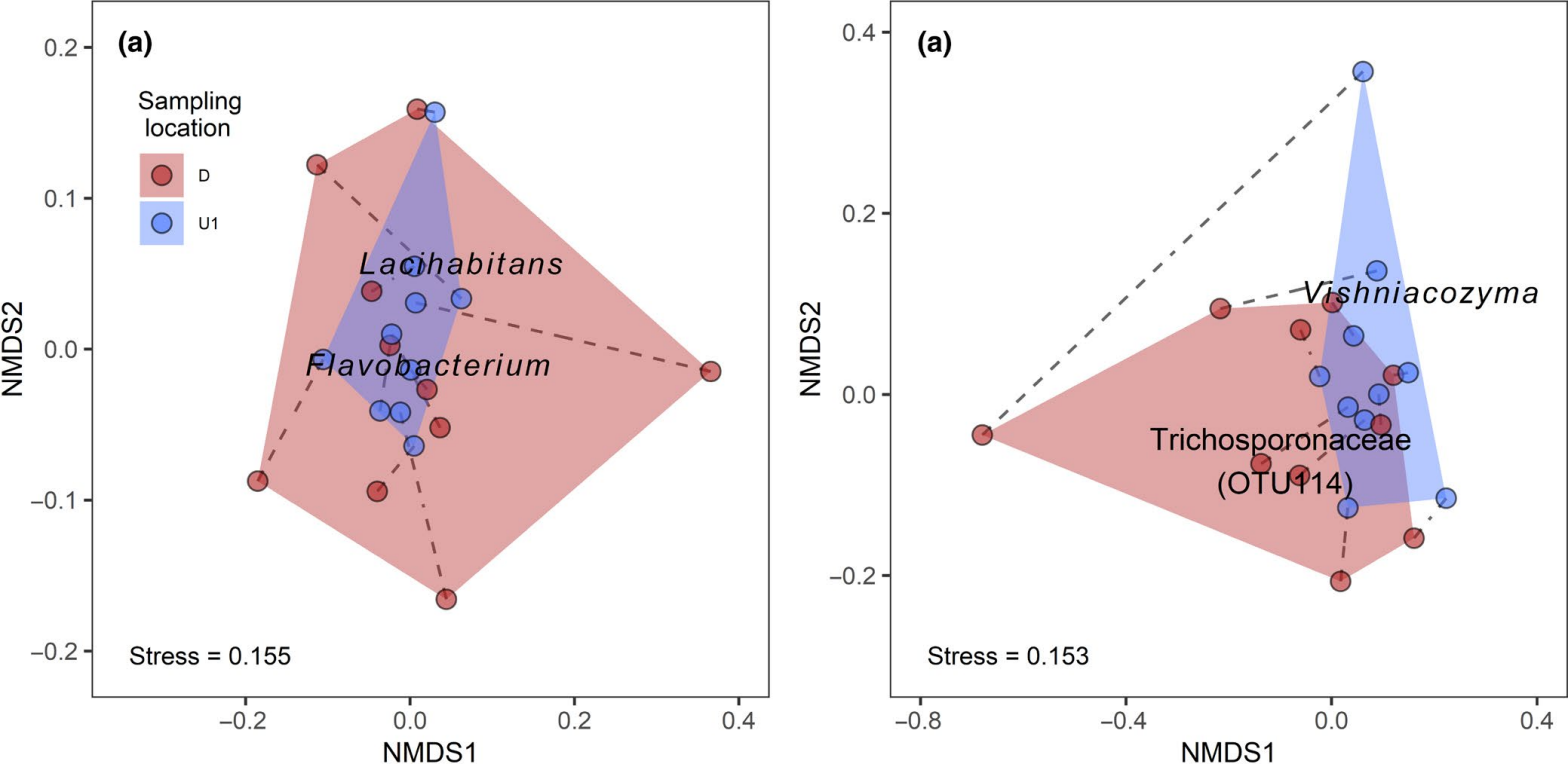
Non‐metric multidimensional scaling (NMDS) analyses showing differences in cotton‐strip microbial community composition characterized by next‐generation sequencing for (a) bacteria (16S rRNA) and (b) fungi (ITS1) using presence–absence data. Communities were sampled from cotton‐strips assays at locations upstream (U1) and downstream (D) of wastewater inputs at 10 study sites sampled in 2013. Examples of potential indicator taxa are shown (see Tables SD5 and SD6 for more information). Dashed lines indicate site pairs, and convex hulls overall differences between sampling locations

Bacterial diversity and composition on CSs responded to WW inputs. Rarefied bacterial OTU richness decreased at downstream sampling locations (Table 2), but the difference was not statistically significant. Instead, we saw a negative influence of non‐fungicides TUs countered by a positive influence of nutrients (Table SD7). Likewise, moderate effect sizes indicated that WW presence had a positive influence on dominant taxa (Berger–Parker index) and a negative influence on rare taxa (Fisher's alpha), but these indicators along with Shannon diversity and Pielou's evenness showed no statistically significant changes (Table 2). Our analysis of bacterial OTU composition showed that downstream communities were significantly different based on presence–absence (“adonis,” *F*_1,17_ = 1.11, *p* < .05, *R* ^2^ = 0.058; Figure 1a) and relative‐abundance data (*F*_1,17_ = 0.83, *p* < .05, *R^2^* = 0.045; Figure SD1). However, only NMDS2 based on relative‐abundance data showed a significant difference between upstream and downstream sampling locations (Table 2), and this indicator was associated with non‐fungicide TUs and mean stream temperatures (Table SD8). Non‐fungicide TUs influenced bacterial OTU occupancy patterns (*F*_1,17_ = 1.26, *p* < .05), but no environmental predictor was significantly associated with bacterial relative abundances in our redundancy analyses (Table SD9). Potential bacterial indicator taxa responding positively to WW inputs included *Flavobacterium* (Figure 1a), *Pseudomonas, Devosia,* and an unidentified species in the Cytophagaceae, whereas bacterial taxa responding negatively to WW included *Lacihabitan*s (Figure 1a), and taxa in the Rhodocyclales and Burkholderiales (Table SD5). Beta‐diversity partitioning indicated that a far greater proportion of community dissimilarity at downstream sites was attributable to turnover of OTUs (Simpson’s index 0.205 ± 0.049, 1 *SD*), as compared with the pure loss (or gain) of OTUs (Nestedness index 0.072 ± 0.065).

**TABLE 2:** Biodiversity indicators based on OTUs from next‐generation sequencing of bacterial and fungal communities on cotton strips (CSs). CSs were incubated at sampling locations downstream (D) and upstream (U1) of wastewater discharges in 10 Swiss streams sampled during Autumn 2013 (*n* = 10). Mean values are presented ±1 *SD*. Cohen's *d* (with Hedge's correction) quantifies differences in responses downstream (D) compared to upstream (U1). Parameter estimates, 95% confidence intervals (CI), *p* values, and the proportion of variance explained by the random factor (“Site”) are presented from mixed models using “Sampling location” (U1, D) as a fixed factor. Diversity, Shannon diversity index (*H*); Evenness, Pielou's *J'*; Dominance, Berger–Parker index; Rareness, Fisher's alpha

Community	Diversity	Indicator	Sampling location		Cohen's *d*	Estimate/IRR	CI	*p*	Site %var.
			U1	D					
Bacteria (16S rRNA)	α	Richness	616 ± 167	549 ± 107	−0.57	0.10	−0.04 to 0.24	.158	49
		Diversity	47.9 ± 6.8	47.4 ± 10.9	−0.04	0.02	−0.12 to 0.16	.759	26
		Evenness	0.621 ± 0.030	0.627 ± 0.026	0.17	−0.02	−0.10 to 0.05	.525	51
		Dominance	0.142 ± 0.037	0.152 ± 0.020	0.43	−0.10	−0.31 to 0.11	.343	25
		Rareness	93.4 ± 17.2	85.8 ± 16.6	−0.42	0.09	−0.05 to 0.22	.199	31
	β	RA NMDS1	0.028 ± 0.174	−0.028 ± 0.238	−0.22	0.06	−0.01 to 0.12	.093	85
		RA NMDS2	0.043 ± 0.179	−0.043 ± 0.192	−0.44	0.09	0.01–0.17	<.05	71
		PA NMDS1	−0.003 ± 0.045	−0.012 ± 0.144	0.04	0.04	−0.09 to 0.08	.883	14
		PA NMDS2	0.014 ± 0.062	−0.014 ± 0.100	−0.25	0.03	−0.01 to 0.07	.163	70
Fungi (ITS1)	α	Richness	559 ± 100	460 ± 162	−0.56	1.22	1.17–1.26	<.001	79
	β	PA NMDS1	0.032 ± 0.013	0.037 ± 0.019	0.24	−0.05	−0.18 to 0.08	.437	22
		PA NMDS2	0.076 ± 0.069	−0.076 ± 0.242	−0.58	0.15	0.01–0.29	<.05	16

Abbreviations: IRR, incident rate ratio; NMDS, non‐metric multidimensional scaling (site scores for Axes 1−2); PA, presence–absence (occupancy); RA, relative abundance (relative counts).

Fungal diversity and composition on CSs responded to inputs of WW. The presence of WW had a significantly negative effect on fungal α‐diversity, as indicated by a decrease in OTUs at downstream locations (Table 2), and there was evidence for negative influences of fungicide TUs (Table SD7). Using presence–absence data, we found that there was a shift in fungal community composition at the downstream locations (“adonis,” *F*_1,17_ = 0.981, *p* < .05, *R* ^2^ = .052; Figure 1b). Our NMDS analyses (presence–absence data) revealed that only NMDS2 showed a significant difference between upstream and downstream (Table 2). Redundancy analysis indicated that MPs and temperature jointly influenced fungal community composition (Table SD9). An unidentified species in the Trichosporonaceae was a potential fungal indicator taxon responding positively to WW inputs, whereas *Vishniacozyma* responded negatively (Figure 1b; Table SD6). Despite the loss of α‐diversity, β‐diversity partitioning within sites indicated that a greater proportion of community dissimilarity was attributable to turnover of OTUs (Simpson’s index 0.376 ± 0.098, 1 *SD*) compared with the pure loss (or gain) of OTUs (Nestedness index 0.054 ± 0.087).

Overall, we found that within‐site changes to fungal community composition were greater than bacteria with significantly higher turnover in response to WW (*t*_19_ = −10.8, *p* < .001). Despite this result, cotton decomposition rates (TS loss) were more strongly associated with bacterial (“rda,” *F*_1,18_ = 3.65, *p* < .05, $R_{\text{adj}}^{2}$ = .12) than fungal (*F*_1,18_ = 1.84, *p* < .05, $R_{\text{adj}}^{2}$ = .04) community composition. Finally, although we were unable to detect significant differences in community dispersion between sampling locations, we found evidence for sampling location‐dependent changes in β‐diversity across sites using beta‐diversity partitioning. Fungal and bacterial communities at WW‐impacted locations showed greater nestedness and overall dissimilarity (“betapart,” *p* < .001) across sites than upstream communities, indicating the potential for WW to contribute to changes in regional diversity patterns (γ‐diversity).

### 3.3 Wastewater disturbance and carbon processing

The CSA showed increased microbial activity downstream of WWTPs. Rates of mass and TS loss were significantly greater at downstream locations (Table 3). Respiration rates (CO_2_ production) on CSs also increased at downstream locations, potentially leading to greater CO_2_ evasion (Table 3). The median areal flux of carbon at upstream locations (U1) was 23.8 kg C m^−2^ year^−1^ (9.5–34.7 kg C m^−2^ year^−1^, 95% CI), whereas this increased to 33.0 kg C m^−2^ year^−1^ (17.9–97.8 kg C m^−2^ year^−1^) below the WWTPs. The rise in carbon efflux at downstream locations represented a median increase of 38.5% (−18.8 to 314.3%, 95% CI). Based on this median estimate, we predicted that CO_2_ evasion from WW‐impacted small–medium streams and rivers in Switzerland potentially adds 29.7 Gg C/year (−21.6 to 249.6 Gg C/year) to the atmosphere. This figure was an order of magnitude lower than an alternative estimate using the difference in median areal carbon efflux between downstream and upstream locations (i.e., 0.34 Tg C/year; −0.04 to 0.84 Tg C/year).

**TABLE 3:** Results of cotton‐strip assay measuring ecosystem functions at sampling locations downstream (D) and upstream (U1, U2) of wastewater discharges in 20 Swiss streams sampled during Autumn 2013 (*n* = 12) and 2014 (*n* = 8). Tensile strength measurements and respiration assays were only performed in the 2013 survey. Mean values are presented ± 1 *SD*. Cohen's *d* (with Hedge's correction) quantifies differences in responses downstream (D) compared to upstream (U1). *F*‐statistics, *df*, *p* values and the proportion of variance explained by the random factor (“Site”) are presented from mixed models using “Sampling location” (U1, U2, D) as a fixed factor

Response	Year	Unit	Sampling location			Cohen's *d*	*F‐*stat	*df*	*p*	Site % Var.
			U2	U1	D	(D, U1)				
Mass loss	All	*k_D_ *(day^−1^)	0.007 ± 0.013	0.006 ± 0.015	0.012 ± 0.013	0.25	22.0	2, 446	<.001	57
		*k*_TD_ * * (1/k_B_ *T* day^−1^)	0.005 ± 0.008	0.005 ± 0.010	0.010 ± 0.011	0.30	34.4	2, 446	<.001	62
TS loss	2013	*k*_D_ * * (day^−1^)	0.032 ± 0.032	0.036 ± 0.027	0.042 ± 0.020	0.92	29.6	2, 266	<.001	60
		*k*_TD_ * * (1/k_B_ *T* day^−1^)	0.016 ± 0.016	0.017 ± 0.013	0.021 ± 0.010	0.96	31.3	2, 266	<.001	60
Respiration	2013	mg C hr^−1^ g^−1^ DM	0.798 ± 0.341	0.811 ± 0.310	0.918 ± 0.218	1.00	21.8	2, 267	<.001	57
Efflux	2013	kg C m^−2^ year^−1^ g^−1^ DM	53.6 ± 54.4	47.5 ± 55.0	82.4 ± 81.3	1.45	35.9	2, 267	<.001	74
Daily water temperature	All	C°	8.67 ± 1.46	8.71 ± 1.43	9.13 ± 1.67	0.33	14.0	2,40	<.001	96
	2013	C°	8.26 ± 1.68	8.32 ± 1.65	8.61 ± 1.84	0.16	5.83	2,22	<.01	98

Abbreviations: DM, dry mass; TS, tensile strength.

**FIGURE 2:**
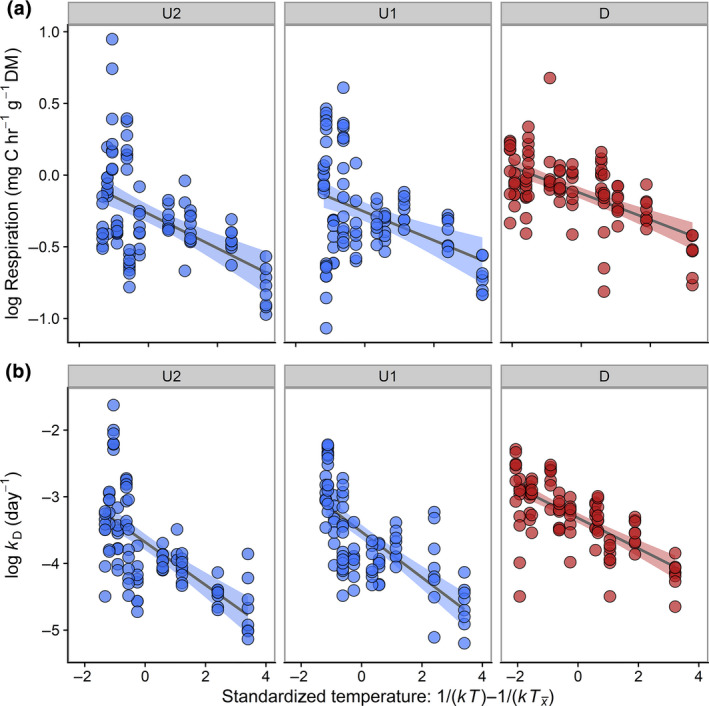
Arrhenius plots across 12 streams sampled in 2013 for individual cotton‐strip (a) respiration and (b) decomposition (measured by rates of tensile strength loss) at locations above (U2, U1) and below (D) wastewater treatment plant (WWTP) discharges. The data reveal that the temperature dependency of cotton‐strip respiration across all three locations—given by the slope of the relationship between the natural logarithm of respiration rates as a function of standardized stream temperature (1/*k*_B_ *T* − 1/*k*_B_ $T_{\bar{x}}$)—were indistinguishable from that predicted by the metabolic theory of ecology (i.e., *E*_a_: 0.6–0.7 eV; Brown et al., 2004). In contrast, activation energies for cotton‐strip decomposition were overall much greater (e.g., *E*_a_: 1.1–1.9 eV) than that predicted by theory, but *E*_a_ was significantly lower (i.e., a flatter slope) at the downstream location D compared to upstream locations U1 and U2. This difference indicates a weaker temperature dependency for decomposition in the presence of WWTP discharges (Table 3)

Stream temperatures were significantly warmer below WW inputs (Table 3), with a mean increase of 0.44°C (0.19–0.69°C, 95% CI). This warming influence supported our decision to assess the temperature dependency of microbial activity on CSs. Although respiration rates were strongly driven by temperature (Figure 2a), there was no significant difference in the activation energy *E*_a_ (i.e., indicated by the slope estimates) of microbial respiration at downstream and upstream locations (Table 4). Furthermore, the estimated slope for all locations (0.73 eV; 0.31–1.14, 95% CI) was similar to the range (0.6–0.7 eV) predicted by theory (Brown et al., 2004). The rates of TS loss (*k*_D_ days^−1^) were also strongly temperature dependent (Figure 2b), yet we observed that *E*_a_ was much greater than predicted by theory across sampling locations (Figure 2b; Table 4). The activation energy of decomposition was influenced by the presence of treated effluent, with a lower *E*_a_ below the WWTP input when compared to the upstream locations (Figure 2b; Table 4). Decomposition rates also differed between the two upstream sites (Table 4), but the effect size (0.05) was much smaller than that between U1 and the WW‐impacted downstream location (0.92).

**TABLE 4:** Results from linear mixed‐models testing the temperature dependency for individual cotton‐strip respiration and decomposition (measured by rates of tensile strength loss) at locations above (U2, U1) and below (D) WWTP discharges in 12 Swiss streams. Temperature is standardized (i.e., 1/*k*_B_ *T* − 1/*k*_B_ $T_{\bar{x}}$)

Response	Predictors	Estimates	CI	Marginal *R* ^2^	Conditional *R* ^2^
log Respiration (mg C hr^−1^ g^−1^ DM)	(Intercept)	−0.28***	−0.40 to −0.16	.309	.642
	Temperature	−0.79***	−1.23 to −0.35		
	U1	0.01	−0.04 to 0.07		
	D	0.14***	0.08–0.20		
	Temperature: Location U1	0.14	−0.10 to 0.39		
	Temperature: Location D	0.15	−0.09 to 0.39		
log *k*_D_ (day^−1^)	(Intercept)	−3.70***	−3.89 to −3.50	.417	.644
	Temperature	−1.73***	−2.50 to −0.96		
	U1	0.14*	0.02–0.25		
	D	0.37***	0.25–0.50		
	Temperature: Location U1	−0.17	−0.65 to 0.32		
	Temperature: Location D	0.59*	0.13–1.06		

Abbreviation: WWTP, wastewater treatment plant.

* *p* < .05;

*** *p* < .001.

**FIGURE 3:**
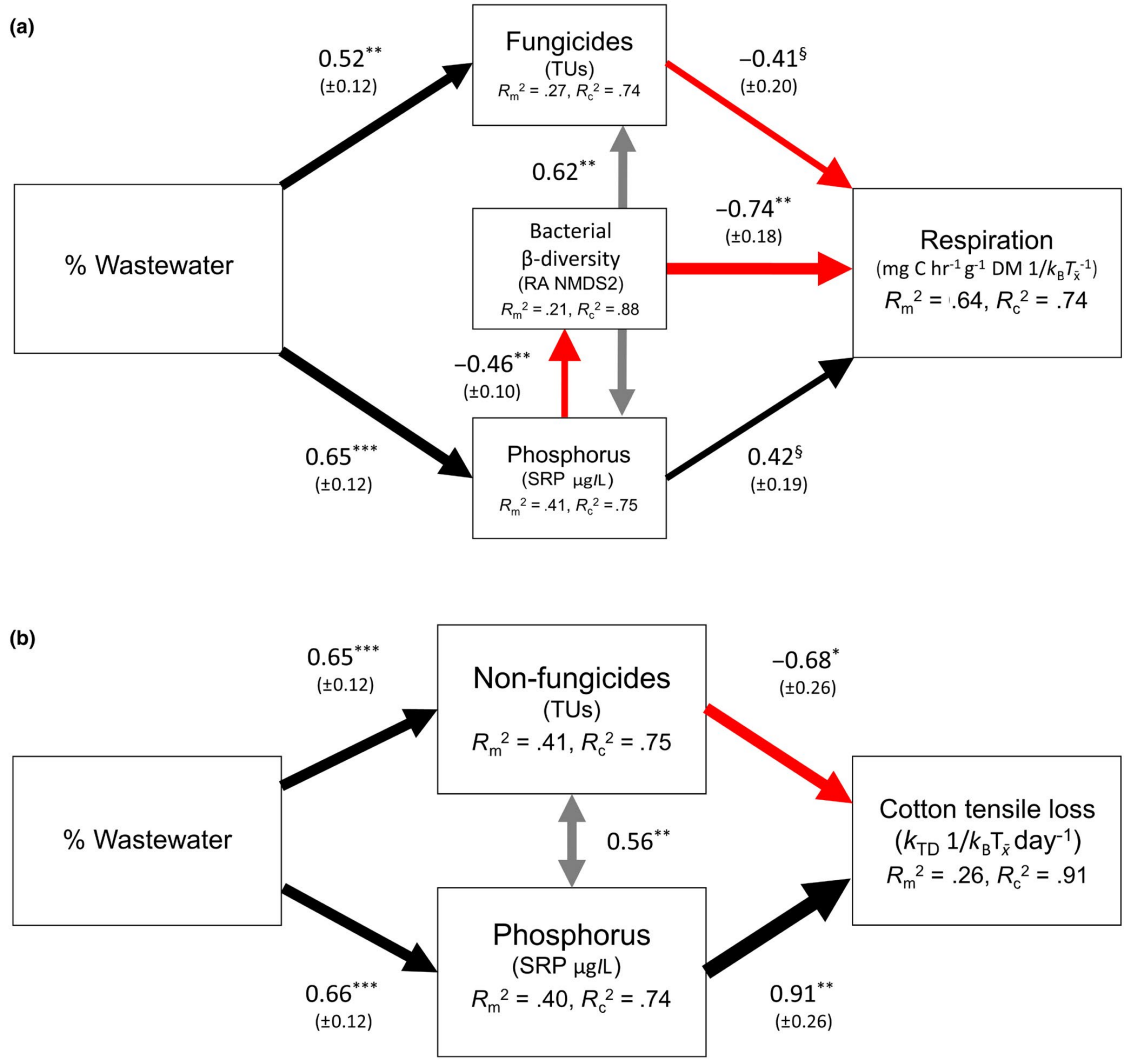
Piecewise structural equation models showing the influence of wastewater inputs on temperature‐corrected indicators of ecosystem functioning as measured by the cotton‐strip assay—rates of (a) respiration and (b) decomposition (tensile strength loss). Mean values are used from ten 2013 study sites with sampling located above (U1) and below (D) wastewater inputs. A random effect term accounts for the non‐independence of site. Solid black lines indicate significant positive influences; red significant negative influences; gray significant correlated errors; all are scaled to the strength of the relationship. Standardized values for path coefficients (±1 *SE*) are indicated. Marginal *R^2^* values indicate the goodness of fit for endogenous variables excluding variance explained by the random effects. Conditional *R^2^* values indicate both fixed and random variance. Model statistics: (a) Fisher's *C* = 3.351, *p* = .764, 6 *df*, ΔBIC = 2.377, (b) Fisher's *C* = 2.967, *p* = .227, 2 *df*, ΔBIC = 0.869. ^§^ *p* < .1; *p < .05; **p < .01; ***p < .001

Using mass‐loss rates recorded at all 20 sites, the first SEM showed no evidence of direct (i.e., unmeasured) effects of WW on decomposition. Instead, positive indirect (i.e., measured) effects appeared to be mediated through concentrations of soluble reactive phosphorus which were positively influenced by WW inputs (Figure SD11). There was no significant influence of other stressors, including MPs (TUs), DIN, or land use (% arable cropping) on cotton mass‐loss rates. However, the second SEM revealed that cotton‐strip respiration rates were influenced by bacterial community composition (β‐diversity; Figure 3a), which, in turn, were indirectly affected by inputs of WW mediated through changes in dissolved phosphorus concentrations. Phosphorus also had a positive direct influence on respiration rates, whereas TUs of fungicides had a negative influence (Figure 3a). Similarly, we found that TS loss was indirectly affected by inputs of WW through a positive influence of phosphorus and a negative influence of non‐fungicide TUs (Figure 3b).

### 3.4 Experimental evidence of altered functioning

**FIGURE 4:**
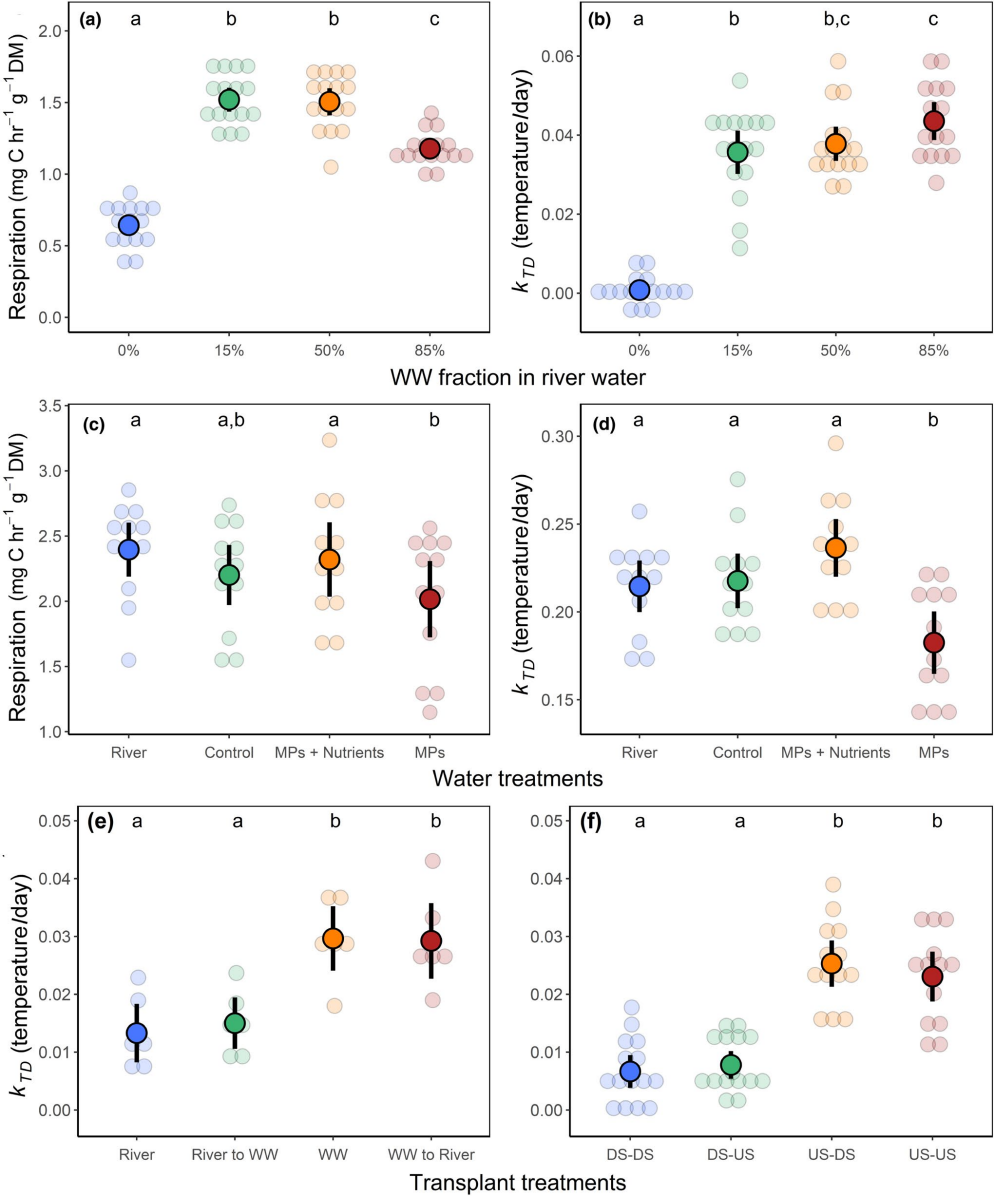
Mean (±95% CI) rates of cotton‐strip respiration and decomposition (tensile strength loss) from the wastewater (WW) “Dilution” experiment (Exp. 1) in the Maiandros flumes system (a, b); the “Dosing” experiment (Exp. 3) in the Maiandros flumes system (c, d); and cotton tensile strength loss rates from the reciprocal‐transplant experiments conducted in (e) the flumes (Exp. 4, Table 1) and (f) the laboratory. To better enable comparisons between the two reciprocal‐transplant experiments, only data from in situ mesocosms are presented from Exp. 4. The laboratory experiment involved cotton strips inoculated at three study sites with locations upstream (US) and downstream (DS) of WW inputs, then laboratory incubated in filtered and sterile river water collected from the field (US/DS locations). For further details regarding flumes experiments, see Table 1

In the flumes experiment with fractions of treated WW (Exp. 1), we found that diluted WW inputs generally increased respiration and TS loss rates (Figure 4a,b). Respiration was the most sensitive response (*F*_3,53_ = 118, *p* < .001), indicating significant differences between all treatments except between the low and medium WW fractions (Figure 4a). Respiration rates at the highest fraction of WW (85%) were lower than that observed in the 15% and 50% WW treatments. In contrast, cotton breakdown responded positively in all WW fractions as demonstrated by TS loss rates (*F*_3,56_ = 89.5, *p* < .01), but there were no significant differences between the medium WW fraction (50%) and the other WW dilutions (Figure 4b). Similar results were observed for mass loss rates ($X_{3}^{2}$ = 120, *p* < .001).

Exp. 2 results are reported in Appendix D because treatments were affected by the methanol carrier. In Exp. 3, we found that MPs had a significantly negative effect on ecosystem functioning as measured by microbial respiration (*F*_3,43_ = 8.06, *p* < .001; Figure 4c), cotton TS loss (*F*_3,41_ = 8.36, *p* < .001; Figure 4d), and mass loss (*F*_3,40_ = 11.2, *p* < .001). There were no significant differences between the “River,” “Technical Control” (i.e., the MeOH carrier treatment), and the combined “MPs and Nutrients” treatment (Figure 4c,d). Respiration rates in the technical control and MPs treatments were not significantly different (“lsmeans,” *t* = 2.37, *p* = .088). However, we did find that comparing effect sizes relative to the river treatment indicated a stronger negative effect in the “MPs” treatment (Cohen's *D* ± 1 *SE*, −0.97 ± 0.50) than in the technical control (−0.49 ± 0.41). Furthermore, we found a small negative effect of the MPs treatments relative to the technical control (−0.33 ± 0.35). Results in Exp. 3 were similar to those in Exp. 2 (Appendix D.2).

The flumes transplant experiment (Exp. 4) showed that inoculation with WW‐borne microbes led to greater cotton TS loss (Figure 4e) that persisted when strips were transferred to river water (*t* = 0.14, *p* = .89). Likewise, the strips inoculated in river water showed no significant change when transferred to diluted WW (*t* = −1.13, *p* = .54). We observed virtually identical responses with mass loss, except for a small relative decrease (−19%) when strips inoculated in WW were transferred to river water (*t* = 3.88, *p* < .001), and a larger relative increase (38%) when strips inoculated in river water were transferred to diluted WW (*t* = −5.23, *p* < .001). These responses may have been due to differences in nutrient concentrations, although caution is needed when interpreting mass‐loss data (Colas et al., 2019).

The laboratory study confirmed the overall pattern of enhanced decomposition through WW inoculation. First, no significant three‐way interactions were observed between location of inoculation, water source, and water treatment (e.g., TS loss, $X_{3}^{2}$ = 1.74, *p* = .63), meaning we tested the two‐way effects (location of inoculation and water source) in each water treatment separately. The *negative* effect of treatment (i.e., filtering and sterilizing) on cotton breakdown was not significant (e.g., TS loss, $X_{1}^{2}$ = 0.83, *p* = .36), and there was no significant interaction with water source ($X_{1}^{2}$ = 0.05, *p* = .83). CSs inoculated in diluted WW showed equivalent rates of TS loss when incubated in filtered river water from upstream and downstream of WWTPs (*t* = 0.94, *p* = .71; Figure 4f), and strips inoculated in river water showed no significant change in TS loss rates when incubated in diluted WW (*t* = 0.81, *p* = .71; Figure 4f). Similar patterns for the water treatments were observed with mass loss and respiration data, and across all three responses with the water control (i.e., no filtering and sterilizing). For all experiment results, see Appendix D.

## 4 DISCUSSION

There is mounting concern about widespread chemical pollution from urban populations (Bernhardt et al., 2017; Wang et al., 2020), and WWTPs are critically important infrastructure helping mitigate this threat (Schwarzenbach et al., 2010). Nonetheless, traditional WW treatment methods may no longer be fit for purpose, reflecting greater societal expectations of environmental quality and the growing multitude of MPs bypassing secondary treatment, thus entering receiving environments (Eggen et al., 2014). We found that the CSA was highly sensitive to warming, nutrients, and inputs of WW, with the net result showing increased microbial activity downstream of WWTPs potentially leading to greater CO_2_ efflux. However, our results deviated from the MTE (Brown et al., 2004), and we found that WW inputs decreased the activation energy of decomposition (i.e., a flatter slope), indicating a “slowing” of this fundamental ecosystem process in response to temperature. SEM suggested a potential reason—negative influences of fungicide and non‐fungicide MPs in WW acted in opposition to the positive influence of dissolved phosphorus concentrations on ecosystem functioning. We then showed experimentally that a realistic mixture of MPs has negative effects on stream ecosystem functioning, but the addition of nutrients “masks” this harmful impact. Contrary to the positive diversity effects predicted by BEF theory (Gessner et al., 2010), we found that process rates increased despite negative influences of WW inputs and MPs on cotton‐strip microbial richness. Instead, net positive WW effects on ecosystem processes likely reflected changes in microbial community composition (β‐diversity) due to environmental filtering (species sorting) and inoculation from the WWTPs. Altered microbial β‐diversity supports the PICT hypothesis (Clements & Rohr, 2009), and may help explain neutral–positive effects of nutrients despite increased concentrations of toxicants (Feckler et al., 2018). Still, we cannot completely rule out positive α‐diversity effects, and the “slowing” response of decomposition to temperature (i.e., decreased activation energies) below WWTPs could be, in part, a consequence of biodiversity loss. Overall, our results highlight key discrepancies in macroecological theory (i.e., MTE, temperature dependency; BEF, positive diversity effects) that suggest a more predictive, transdisciplinary approach incorporating ecotoxicological perspectives is needed to understand interactions between chemical stressors, biodiversity change, and ecosystem functioning.

### 4.1 Subsidy‐stress effects of multiple stressors

Anthropogenic stressors typically have antagonistic interactions when combined (Jackson et al., 2016). Our results (e.g., Figure 4a) further highlight the potential for “subsidy‐stress” effects of WW inputs on stream ecosystems (Aristi et al., 2015). Nutrients and warming stimulate certain processes, but toxicants can exert negative impacts on functioning. We used field data (Figure 3) and flume experiments (Figure 4c,d) to demonstrate competing positive and negative influences of nutrients and MPs, respectively, on ecosystem process rates. Similarly, Aristi et al. (2016) observed only antagonistic interactions between nutrients and MPs in an experiment involving artificial streams, as high nutrient concentrations compensated for the harmful effects of the toxicants on community respiration. Other studies have shown that stressors with expected negative effects act in opposition to nutrients. Bruder, Salis, McHugh, and Matthaei (2016) found that addition of fine inorganic sediment negated positive nutrient effects on litter decomposition. However, Feckler et al. (2018) showed that historical contingencies can modify stream microbial functioning in response to multiple stressors: communities from a disturbed agricultural stream demonstrated PICT. This resulted in an assemblage dominated by a few tolerant species with high litter breakdown efficiencies, thus explaining why functioning was maintained under exposure to fungicides at different nutrient concentrations. In contrast, breakdown rates with the pristine microbial community were greatly reduced under stress, further demonstrating that environmental context can determine biotic responses to stressors by reflecting historical “legacy effects” on community tolerances (Burdon et al., 2016).

Across all sampling locations and sites, there was evidence for a positive relationship between stream temperature and microbial activity. The activation energies (*E*_a_) we observed using microbial respiration on the CSs (*E*_a_: 0.73 eV) conformed closely with that predicted by metabolic theory (0.6–0.7 eV; Brown et al., 2004). In contrast, *E*_a_ for decomposition rates were much greater, despite a relative decrease in the presence of WW (Figure 2b). Our *E*_a_ values for decomposition also contrasted with a global CSA study where activation energies for this indicator were identical to theoretical predictions (Tiegs et al., 2019). Boyero et al. (2011) showed similar activation energies (0.46 eV; 0.02–1.00, 95% CI) in leaf breakdown rates of alder (*Alnus glutinosa*) over a global stream temperature gradient, whereas a meta‐analysis (Follstad Shah et al., 2017) indicates that the *E*_a_ for decomposition across a wider range of plant genera may be even lower (0.34 eV; 0.27–0.40, 95% CI). However, in a localized study of a temperate‐zone forested stream, Griffiths and Tiegs (2016) reported an *E*_a_ of 1.41 eV (1.07–1.74, 95% CI) for the CSA, which was more similar to the values we recorded. Part of this discrepancy may be an artefact of the relatively narrow mean temperature range in our study (e.g., 4.26–10.64°C at location D), meaning the slope of the relationship is potentially sensitive to values at either end of the gradient.

Warming can have synergistic interactions with nutrient enrichment, thus further increasing rates of microbial decomposition in streams (Fernandes, Seena, Pascoal, & Cássio, 2014; Ferreira & Chauvet, 2011). However, despite the potential for this interaction at the locations downstream of WWTPs where nutrient concentrations were highest, the activation energies of decomposition were lower. This indicates that over the same temperature range, decomposition would proceed slower at the downstream locations (elevated nutrient concentrations notwithstanding). Brown et al. (2004) highlighted that residual variation in decomposition not explained by temperature could be accounted for by other factors, such as resource stoichiometry (i.e., C:N ratios). However, nutrient content of organic matter typically increases with microbial biomass accrual (Gulis & Suberkropp, 2003), suggesting that in our example using a standardized functional indicator (i.e., the CSA), systematic changes with temperature in the presence of WW (e.g., slower fungal growth rates) might explain the reduction in activation energies. This discrepancy could be directly caused by negative effects of MPs in the WW effluent, or indirectly through reductions in microbial α‐diversity. Our findings show evidence for complex multiple‐stressor interactions between warming, nutrients, and toxicants that need to be further explored (e.g., measuring resource stoichiometry and fungal biomass) so that ecological risks can be accurately identified and mitigated.

### 4.2 Biodiversity and ecosystem functioning

Contrary to one of our main predictions (BEF, positive α‐diversity effects), but supporting an alternative hypothesis (i.e., PICT), enhanced stream ecosystem functioning was associated with changes in microbial β‐diversity in the presence of WW. Similar to Wakelin et al. (2008), we saw evidence of environmental filtering (species sorting) with negative WW effects on bacteria from the Rhodocyclales and Burkholderiales, yet turnover dominated nestedness in our analyses partitioning microbial β‐diversity. Microbes that are characteristic of heavily degraded ecosystems can be more stress tolerant (sensu PICT), enabling them to maintain process rates in the face of multiple stressors and thus buffering ecosystem functioning against biodiversity losses (Feckler et al., 2018; Gardeström, Ermold, Goedkoop, & McKie, 2016).

A key tenet of the PICT paradigm predicts functional redundancy in stream microbial assemblages. Fungi typically contribute more to decomposition than bacteria (Hieber & Gessner, 2002), and functional redundancy in fungal hyphomycete species is expected to be low because different taxa possess complementary enzymes targeting different chemical compounds found in natural organic matter and activity rates vary with the successional stages of decomposition (Gessner et al., 2010). However, the CSs with their relatively high proportion of cellulose may be less complex chemically than natural organic matter, and more equivalent to precursor carbon substrates found in WWTP influent. In our study, bacterial communities seemingly contributed more to cotton‐strip decomposition, and we identified *Flavobacterium* as a potential indicator taxon responding positively to WWTP inputs. Although widely distributed in freshwaters, *Flavobacterium* is considered a core genus in activated sludge systems where it can represent up to 60% of the viable flora (Gonzalez‐Martinez et al., 2016; Shewan & McMeekin, 1983) and it was a dominant taxon in effluent from a Chinese WWTP (Chen et al., 2019). Although *Flavobacterium* can degrade cellulose (Kim & Yu, 2020), they are more known as a potential pathogen to fish (Starliper & Schill, 2011), and it could be that taxa in the Cytophagaceae were also contributing to decomposition below the WWTPs (Kirchman, 2002).

Combining results from the two transplant experiments, we showed that WW effluent can enhance cotton breakdown via inoculation. However, we were unable to test whether this “ghost of inoculation past” was mediated more by increased colonization rates (mass effects; Heino, 2013) or growth rates of microbes in the presence of WW (priority effects; Vannette & Fukami, 2014). Bacterial analyses (16S rRNA) of water samples collected from our study sites suggested that indicator taxa like *Flavobacterium* (Flavobacteriaceae) were not more prevalent in the effluent (Mansfeldt et al., 2020), thus suggesting that priority effects may have been more important than mass effects. Mansfeldt et al. (2020) did show, however, that downstream bacterial assemblages were different with a noticeable increase in microbes associated with the human microbiome (e.g., *Ruminococcus*). While the export of microbes from WWTPs may help to stabilize stream ecosystem functioning in the face of increased stress from MPs, this connectivity also has the potential to increase the occurrence and persistence of pathogens and antibiotic resistance genes in receiving environments (Proia et al., 2016).

### 4.3 Wastewater inputs and carbon efflux

Understanding the effects of chemical pollution on stream and river CO_2_ emissions is essential for predicting how these dynamics will respond to future environmental change. Our results and previous studies suggest that stream ecosystem respiration typically increases due to inputs of WW (Aristi et al., 2015; Gücker, Brauns, & Pusch, 2006; Young, Matthaei, & Townsend, 2008). We estimated that WW inputs in small–medium streams and rivers could add 29.7 Gg C/year to the atmosphere at the Swiss national scale. This carbon efflux represents an additional 0.90 kg C m^−2^ year^−1^ , or an increase of 37.8%–52.6% based on the average 1.70–2.37 kg C m^−2^ year^−1^ for streams and rivers in the conterminous USA (Butman & Raymond, 2011; Butman et al., 2016), thus overlapping with our estimated median increase of 38.5%. In contrast, our median estimates of areal carbon efflux at upstream (22.8 kg C m^−2^ year^−1^) and WW‐impacted locations (33.0 kg C m^−2^ year^−1^) indicate that our second approach overestimated increases in carbon fluxes for Switzerland (i.e., 0.34 Tg C/year). Our areal estimates were high when compared to stream types that are considered significant sources of CO_2_ evasion: Swiss mountain streams (median 3.5 kg C m^−2^ year^−1^; −0.5 to 23.5 kg C m^−2^ year^−1^, 90% CI; Horgby et al., 2019) and Swedish boreal streams (range 1.5–6.4 kg C m^−2^ year^−1^; Wallin et al., 2013); but cf. Amazonian rivers during extreme floods (range 0.6–12.3 kg C m^−2^ day^−1^; Almeida, Pacheco, Barros, Rosi, & Roland, 2017). This discrepancy is unsurprising because the CS is organic matter, thus inflating the respiration values our estimates are based on, and helps justify our primary approach using the relative change in efflux as opposed to absolute values.

However, both our approaches for upscaling carbon efflux assume homogeneous WW‐impacts longitudinally downstream, which are unlikely due to landscape heterogeneity and uncertainties in organic carbon spiraling lengths (Aristi et al., 2015; Young & Huryn, 1999). Although our results from the first flumes experiment (Exp. 1) suggest that respiration and carbon efflux may have a nonlinear relationship with WW concentrations (Figure 4a), other researchers have suggested that ecosystem respiration increases monotonically with WW fractions (Pereda, Acuña, von Schiller, Sabater, & Elosegi, 2019). This means that streams with low or no WW dilution potential could have higher rates of carbon evasion. Likewise, our estimates do not account for seasonality, which influence organic‐matter inputs, flow rates, and temperature (Allen & Pavelsky, 2018; Caissie, 2006; Hotchkiss et al., 2015). Nonetheless, while there are caveats to recognize, our estimates suggest that inputs of WW to streams and rivers could be a significant source of atmospheric carbon.

### 4.4 Concluding remarks

We used a wide range of approaches to study the effects of WW and associated stressors on a fundamental stream ecosystem process (i.e., organic‐matter decomposition) using the CSA. Our study shows that WW‐born MPs have the potential to harm ecosystem processes as indicated by the CSA, but these impacts may be “masked” by nutrients, warming, and stress‐tolerant microbes. While we did find significant net positive effects of WW inputs on this functional indicator, the “slowing” of responses to warming across the temperature gradient indicates that the buffering capacity of microbial communities may be limited. This result, coupled with negative influences of fungicides on fungal richness, add to the growing concern about the threat this pesticide class poses to biodiversity and ecosystem functioning (Zubrod et al., 2019). Our findings point to the need for further experiments to manipulate abiotic and biotic drivers to better understand ecological limits and underlying mechanisms of change in freshwater ecosystems (Burdon et al., 2016).

Wastewater treatment plants are vitally important infrastructure for managing water resources (Schwarzenbach et al., 2010), and several developed countries are upgrading their facilities to help mitigate the growing threat from MPs (Stamm et al., 2016). Measuring ecological responses to upgraded infrastructure indicates the return of these investments and helps underscore the societal relevance of our research. Our results suggest that the subtle negative effects of MPs on microbial‐mediated decomposition could be ameliorated with tertiary treatment steps (e.g., ozonation) that remove MPs. Thus, decomposition rates measured by the CSA should increase post‐upgrade, signifying the potential for this functional indicator as a useful baseline to assess the efficacy of mitigation. However, such improvements may generate undesirable side effects. Our estimates suggest that WWTP inputs led to a median 39% increase in CO_2_ evasion rates. This efflux meant that in Switzerland there could be an additional 30,000 tons of carbon entering the atmosphere annually from WW‐impacted streams and rivers, with the potential to further increase with improved treatment that removes MPs.

In a broader sense, our findings demonstrate the value of the CSA as a standardized method to measure microbial communities and organic‐matter decomposition, helping to emphasize its utility as a functional indicator for ecosystem assessment (Colas et al., 2019; Tiegs et al., 2019). We found that the CSA was highly sensitive to a variety of environmental factors across field, flumes, and laboratory settings. Future research should describe microbial biodiversity in more detail to map structure to function, and further compare the relative performance of this functional indicator with natural leaf litter and other standardized organic‐matter surrogates. Integrating knowledge of structural changes in stream communities with functional consequences will help to predict the impacts of synthetic chemicals and other global change drivers. Overall, our study further highlights the need to manage for multiple stressors when considering human impacts on the environment. Finally, we conclude that further reconciling macroecological and ecotoxicological perspectives is required to develop the transdisciplinary approach needed to understand interactions between environmental stressors, biodiversity change, and ecosystem functioning.

## Supporting information

Supplementary Material

## Data Availability

Data will be made available via the Dryad Digital Repository (Burdon, Bai, et al., 2020).
