# Supplementary material for: Stream microbial communities and ecosystem functioning show complex responses to multiple stressors in wastewater
Source: Glob Chang Biol. 2020 Sep 3;26(11):6363–82. doi: 10.1111/gcb.15302 (PMC7692915; doi:10.1111/gcb.15302)
Supplement: Supplementary file 1 — Supplementary Material [file GCB-26-6363-s001.docx]

Supporting Information for:

**Stream microbial communities and ecosystem functioning show complex responses to multiple stressors in wastewater**

F.J. Burdon^1,2^, Y. Bai^3^, M. Reyes^1^, M. Tamminen^1,‡^, P. Staudacher^1^, S. Mangold^1§^, H. Singer^1^, K. Räsänen^1,5^, A. Joss^1^, S.C. Tiegs^4^, J. Jokela^1,5^, R.I.L. Eggen^1,6^, and C. Stamm^1^

1. Eawag, Swiss Federal Institute of Aquatic Science and Technology, Dübendorf, Switzerland
2. Department of Aquatic Sciences and Assessment, Swedish University of Agricultural Sciences, Uppsala, Sweden.
3. Research Center for Eco-Environmental Sciences, Chinese Academy of Sciences, Beijing, People's Republic of China
4. Department of Biological Sciences, Oakland University, Rochester MI, USA.
5. ETH Zurich, Institute of Integrative Biology, Swiss Federal Institute of Technology, Zurich, Switzerland
6. ETH Zürich, Institute of Biogeochemistry and Pollutant Dynamics, Zürich, Switzerland

^‡:^ Current address: Department of Biology, University of Turku, Turku, Finland

^§:^ Current address: Agroscope, Reckenholzstrasse 191, 8046 Zürich, Switzerland

Corresponding author: Dr. Francis J. Burdon

Department of Aquatic Sciences and Assessment,

Swedish University of Agricultural Sciences,

P.O. Box 7050, 75 007 Uppsala, Sweden

[francis.burdon@slu.se](mailto:francis.burdon@slu.se)

Phone +46 78 594 2321

# Introduction

The Supporting Information is structured in four parts:

1. Detailed information regarding the study sites and design (Appendix A)
2. Overview of the Maiandros flumes system (Appendix B)
3. Detailed descriptions of methods (Appendix C)
4. Additional results complementing the main text (Appendix D)

# Appendix A: Description of study sites

Twenty study sites located across Switzerland were selected to investigate WWTP impacts on receiving stream ecosystems (Burdon et al. 2016, Stamm et al. 2016). At each study site, we designated one downstream sampling location (D), and two upstream sampling locations (U1, U2; Stamm et al. 2016). Location D was selected so that discharged WW was completely mixed across the wetted channel. Location U1 was chosen as close to the discharge as possible, and U2 approximately equidistant to that between U1 and D.

**
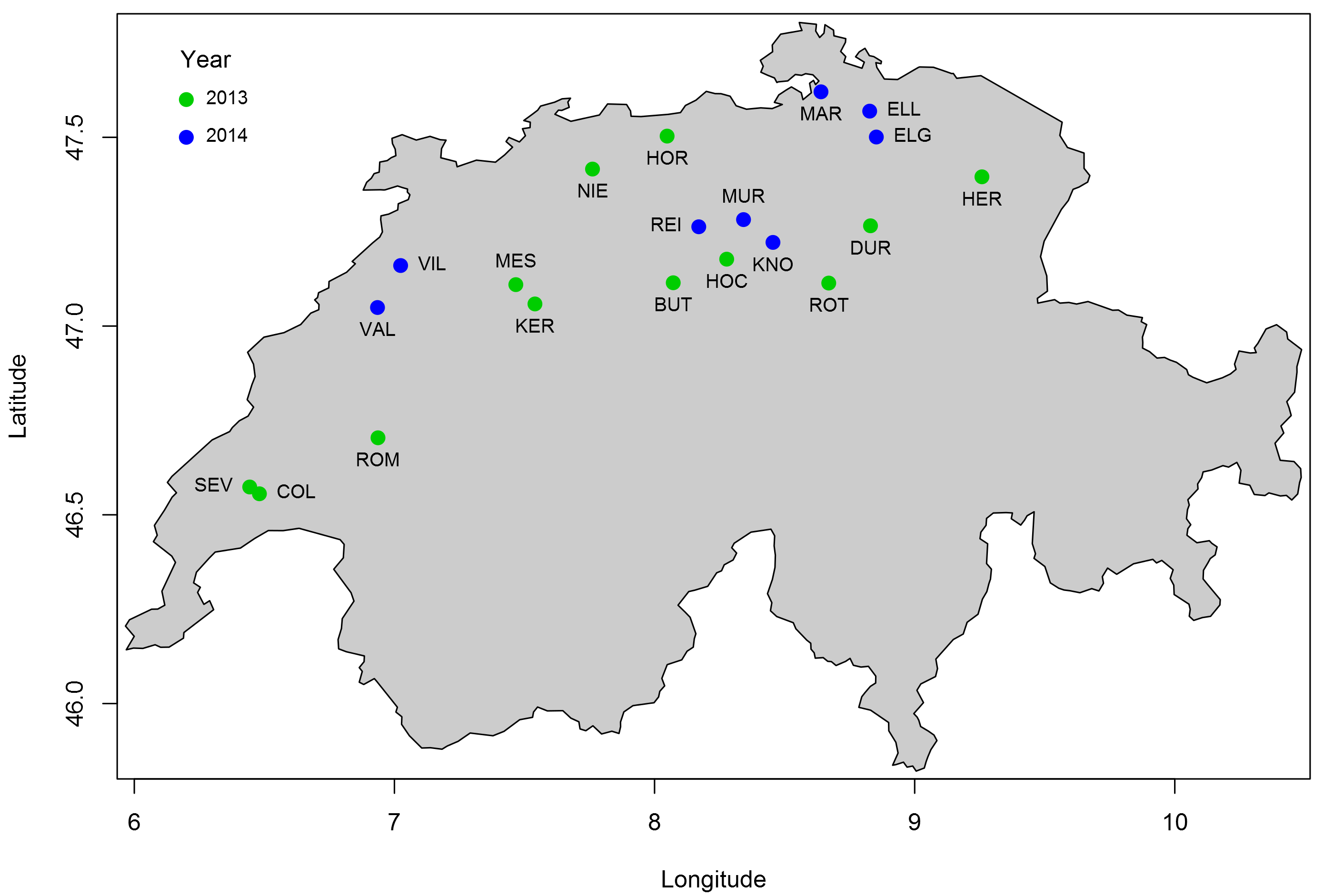
**

**Figure SA1** Map of study sites in Switzerland where the cotton-strip assay was deployed during the field experiment (12 sites in 2013, and 8 sites in 2014).

**Table SA1** Characteristics of the field study sites. Land use data refer to the hydrological catchment upstream of the respective wastewater treatment plant (WWTP). Q_347_: river discharge exceeded 95% of the time, Q_mean_: mean river discharge, %WW_Qmean: fraction of wastewater (WW) downstream of the WWTP assuming average WW discharge and Q_mean_, %WW_Q347: fraction of wastewater (WW) downstream of the WWTP assuming average WW discharge and Q_347_, CSA_field: field sites included in the CSA analyses, ARISA: field sites where microbial communities on the cotton strips were analysed using ARISA (C.2.4) and NGS (see Methods section, main text), Inoculation: CSA inoculated for laboratory experiment (see Methods section, main text).


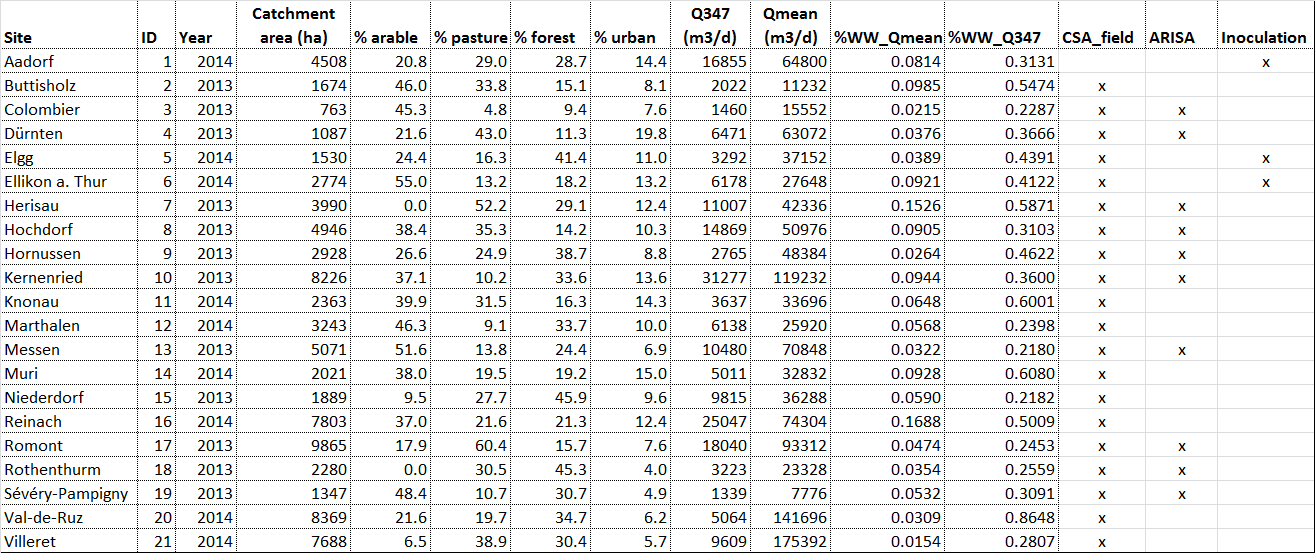


# Appendix B: Description of the Maiandros flume system

Maiandros enables contaminant concentrations (e.g., wastewater, micropollutants, nutrients) to be manipulated through dilution and/or dosing experiments (Stamm et al. 2016). Maiandros has a total of 16 channels (2.6 m circulation path length, 0.15 m channel width, 0.05 m water depth), configured in four experimental ‘blocks’. Channels are semi-recirculating, with water driven by paddle-wheels around a ‘race-track’ design (0.2 m/s flow speed, 10-12 min hydraulic residence time). We equipped all channels with temperature loggers (e.g., HOBO Pendant UA-002-64, Onset Computer Corporation, Bourne, MA, USA).


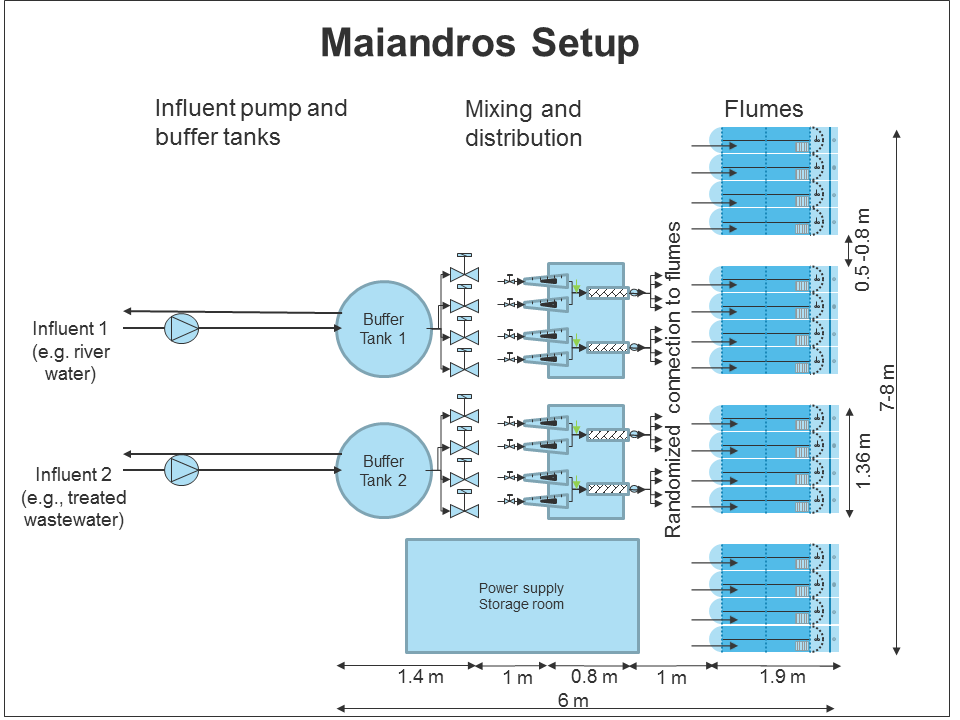


**Figure SB1** Layout of the Maiandros flume system.


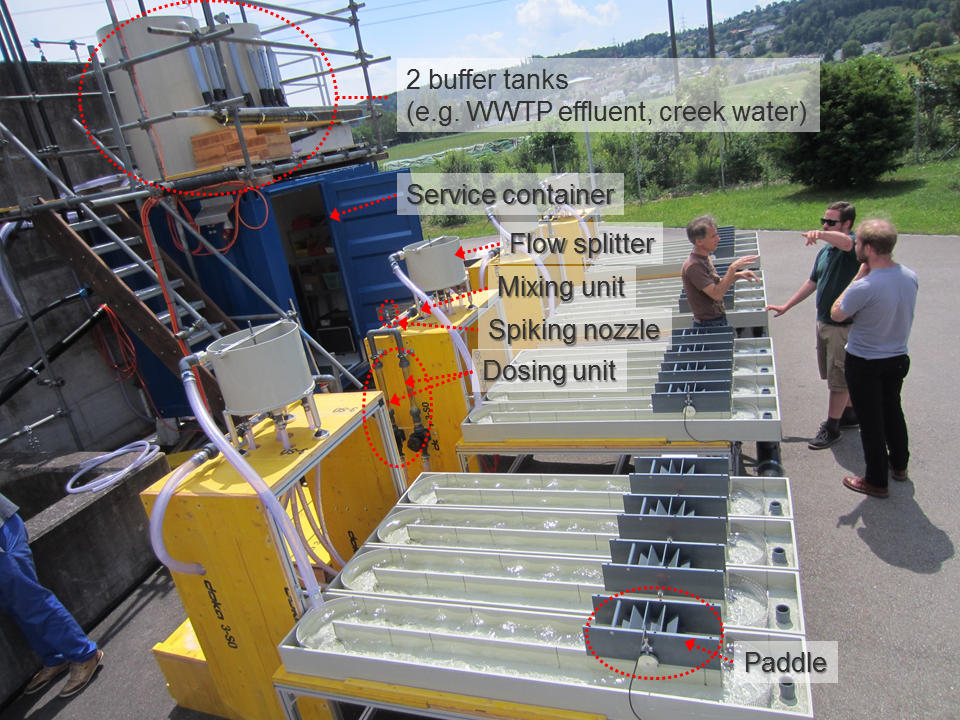


**Figure SB2** Picture of the Maiandros system after the initial set-up on the WWTP Bachwis.


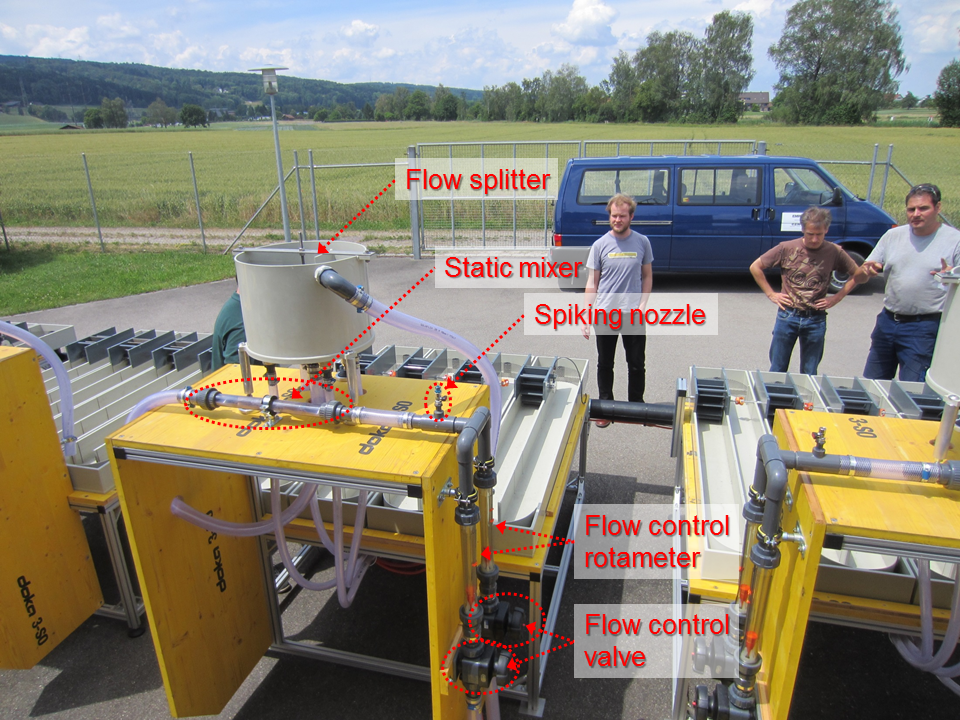


**Figure SB3** Details of the Maiandros distribution, mixing and dosing system after the initial set-up on the WWTP Bachwies.

# Appendix C: Detailed methods descriptions

## C.1 Cotton-strip assay (CSA)

### C.1.1 General description

The CSA quantifies the inherent capacity of ecosystems to process organic carbon, i.e., their decomposition potential, and integrates the effects of microbial community structure and environmental factors, including nutrient availability, temperature, and microbial activity. Consisting of >95% cellulose, the substrate is highly relevant in streams where carbon cycles are often dominated by inputs of leaf litter, of which cellulose is the key constituent.

We cut cotton strips (25 mm × 80 mm) from the same bolt of ‘Artists canvas’ fabric (Slocum et al. 2009). The “Artists canvas” fabric has been demonstrated to be a highly effective cotton material for stream biomonitoring purposes (Colas et al. 2019). The molar C:N of the cotton strip before incubation is 276 (*pers. comm*. Dr. Dave Costello, Kent State University, OH, USA). Before deployment in the field, a small incision was made in each strip near one end, and a plastic cable tie used to fix it to the lengths of nylon cord.

Retrieval rates of strips were excellent with 99.7% in 2013 and 97.4% in 2014. Five strips from sampling location U2 at a 2013 site (ROM) were excluded from our analyses because a recent flood had left the strips exposed on the bank at the time of retrieval. Only mass-loss data was recorded from the 2014 cotton strips.

### C.1.2 Cotton-strip respiration assay

We used the cotton-strip respiration assay described by Tiegs et al. (2013). At the time of collection, we carefully cleaned detached strips with water to remove sediment before inserting them individually into respiration chambers (50-mL flat-bottomed plastic centrifuge tubes). Each chamber was filled with stream water without air bubbles. Three blank chambers were filled with only stream water. We measured dissolved oxygen (DO; mg/L) in the stream at this time using a handheld probe (Hach HQ40d, Loveland, CO, USA) to establish initial DO concentrations. We incubated chambers in the dark at stream temperature for approximately 2-h. At the end of the assay DO was measured by placing the probe directly into the respiration chambers.

### C.1.3 Respiration of carbon dioxide

We wanted to estimate carbon dioxide production rates using the oxygen consumption rates measured using the cotton-strip respiration assay. First, we used a relationship between microbial consumption rates of dissolved oxygen and CO_2_ production in freshwaters to convert our respiration measurements into units of carbon dioxide. Following Berggren et al. (2012), we used Eq.SC1:

| ${TCO}_{2}=1.23C{{DO}_{2}}^{0.99}$ | (Eq.SC1) |
| --- | --- |

where *TCO_2_* is the rate of microbial-produced total carbon dioxide (μM day^-1^) and *CDO_2_* is the consumption rate of dissolved oxygen (μM day^-1^). We then used these values to express cotton strip respiration as rates of carbon dioxide production standardised for the remaining dry mass of the cotton strip (mg C g DM^-1^ hr^-1^). Rates were calculated for net, water (i.e., blanks), and cotton strip (i.e., net minus blanks) respiration. The approach following Berggren et al. (2012) compares favorably to assuming a respiratory quotient (RQ) of 1:1, an approach which has been previously used in the stream organic-matter processing literature when data for actual RQs are unavailable (e.g., Gulis and Suberkropp 2003, Ferreira and Chauvet 2011) .

### C.1.4 Carbon efflux

We wanted to use the rates of CO_2_ production from the CSA respiration assay to estimate carbon efflux. These estimates would use a standardized functional indicator (i.e., the CSA) to indicate how inputs of WW and experimental treatments alter CO_2_ evasion from receiving freshwater habitats in our study. This approach would be useful for upscaling our results to broader spatial and temporal scales. The flux of CO_2_ depends mainly on two factors: the concentration gradient between the surface water and the air and the gas exchange coefficient for CO_2_ at a given temperature (Wanninkhof 1992, Raymond et al. 1997). The concentration gradient is expressed as the difference between the actual concentration of CO_2_ in the water and the concentration that water would have, were it in equilibrium with the atmosphere (Raymond et al. 1997). The physical transfer or turbulent energy at the water-air interface should also be factored in (Wanninkhof 1992, Maclntyre et al. 1995). Thus, we aimed to estimate carbon efflux with the general equation (Eq.SC2):

| ${CO}_{2} Flux=k \times\left[ \left( {pCO}_{2} \times kH \right)- \left[ {CO}_{2} \right] \right]$ | (Eq.SC2) |
| --- | --- |

Where *k* is the gas transfer velocity for CO_2_, *pCO_2_* is the partial pressure of CO_2_ in the surface water, *kH* is Henry's constant for CO_2_ at a given temperature, and [*CO_2_*] is the concentration of CO_2_ in water at equilibrium with the overlying atmosphere. However, to estimate these parameters using our data required numerous incremental steps based on the literature. To assign a gas transfer velocity for study sites we used a statistical model (Eq.SC3; *R^2^* = 0.76, *P* < 0.001) from a meta-data analysis of measurements of gas exchange and the gas transfer velocity made by direct gas tracer releases (Raymond et al. 2012):

| $k_{600}=4725 \times\left( VS \right)^{0.86} \times Q^{-0.14} \times D^{0.66}$ | (Eq.SC3) |
| --- | --- |

where *k*_600_ (m d^-1^) is the gas exchange velocity based on stream velocity (*V*, m s^-1^), slope (*S*; unitless), depth (*D*, m) and discharge (*Q*, m^3^ s ^-1^). Methods for the collection of hydrogeomorphological data are decribed below (C.2.1) Following Jähne et al. (1987), we accounted for mean stream temperatures using *ScCO_2_*, the Schmidt number before normalization to 600 (Eq.SC4) which depends on the water temperature *T* (K).

| ${ScCO}_{2}=1841 \times{2.713}^{-0.0549(T-273.15)}$ | (Eq.SC4) |
| --- | --- |

The gas transfer velocity of CO_2_ was normalised to 20°C (*k*_600_) using a Schmidt number (*Sc*) for 20°C (Eq.SC5) following (Jähne et al., 1987). *Sc* is power dependent and for the more turbulent waters of streams we used an exponent of -0.5.

| ${kCO}_{2}=\frac{k_{600}}{{600}/{{{ScCO}_{2}}^{-0.5}}}$ | (Eq.SC5) |
| --- | --- |

We then calculated the gas exchange coefficient *k* (hr^-1^) based on the conditions of the respiration assay, which is conducted in a microcosm chamber (i.e., a 50 ml centrifuge tube). To do this, we adapted the equation provided by Demars and Manson (2013) as Eq.SC6:

| $k=\frac{{(kCO}_{2}\times1/{24}) \times A}{V}$ | (Eq.SC6) |
| --- | --- |

where *A* is the area of the respiration chamber (7.069 × 10^-4^ m^2^) and *V* the volume (5.5 × 10^-5^ m^3^), and *kCO_2_* (m d^-1^) is converted into m hr^-1^.

To estimate the partial pressure of dissolved *CO_2_* we first had to estimate the saturated concentration of carbon dioxide [*CO_2_*]. We first calculated the temperature-dependent solubility coefficient (*KO*) for CO_2_ using Eq.SC7 reported by Weiss (1974):

| $log\left[ KO \right]= -58.09+90.506 \times\left( \frac{100}{T} \right)+22.294 \times log\left( \frac{100}{T} \right)$ | (Eq.SC7) |
| --- | --- |

where *KO* is expressed as mol l^-1^ atm^-1^ and temperature *T* is in Kelvins (K). Following Henry´s law, *KO* was expressed as an inverse (i.e., thus equivalent to the Henry volatility defined via concentration *kH*). We then calculated an estimate of saturated CO_2_ concentrations using Henry´s Law (Eq.SC8)

| $\left[ {CO}_{2} \right]=\frac{P}{kH}$ | (Eq.SC8) |
| --- | --- |

where *P* is the partial pressure of CO_2_ in the atmosphere (3.95 × 10^-4^ atm in 2013; Blunden and Arndt 2014) and *kH* the temperature-corrected Henry’s Law constant for CO_2_ (based on Eq.SC7) expressed here as (L . atm) mol^-1^. Concentrations were expressed as mol l^-1^.

We considered that CO_2_ production (i.e., respiration) would be additive to saturated concentrations of carbon dioxide. Thus, the net rise in dissolved carbon dioxide was estimated as a combination of saturated *CO_2_* (aq) and hourly respiration on the cotton strip (uncorrected for the respiration in the water; Eq.SC9a). We separately estimated the rise in dissolved carbon dioxide from the blanks (stream water without a cotton strip) and the cotton strip (minus the blanks) using Eq.SC9b-c:

| $net.{CO}_{2}= net.{TCO}_{2}+ \left[ {CO}_{2} \right]$ | (Eq.SC9a) |
| --- | --- |
| $water.{CO}_{2}= water.{TCO}_{2}+ \left[ {CO}_{2} \right]$ | (Eq.SC9b) |
| $csa{.CO}_{2}= csa.{TCO}_{2}+ \left[ {CO}_{2} \right]$ | (Eq.SC9c) |

where the units of CO_2_ (*net*, *water*, and *csa*) were expressed as mol l^-1^ hr^-1^.

Following Henry´s law, *KO* was expressed as an inverse (i.e., thus equivalent to *kH*) and then multiplied by the hourly rise in dissolved carbon dioxide (mol l^-1^) using Eq.SC10a-c adapted from Weiss (1974).

| $net.p{CO}_{2}= kH\times net.{CO}_{2}$ | (Eq.SC10a) |
| --- | --- |
| $water.p{CO}_{2}= kH\times water.{CO}_{2}$ | (Eq.SC10b) |
| $csa.p{CO}_{2}= kH\times csa.{CO}_{2}$ | (Eq.SC10c) |

Using these parameters, we estimated CO_2_ gas flux as stated above (Eq.SC2) using Eq.SC11a-c:

| $net.{CO}_{2} Flux=k \times\left[ \left( net.{pCO}_{2} \times kH \right)- \left[ {CO}_{2} \right] \right]$ | (Eq.SC11a) |
| --- | --- |
| ${water.CO}_{2} Flux=k \times\left[ \left( water.{pCO}_{2} \times kH \right)- \left[ {CO}_{2} \right] \right]$ | (Eq.SC11b) |
| ${csa.CO}_{2} Flux=k \times\left[ \left( {csa.pCO}_{2} \times kH \right)- \left[ {CO}_{2} \right] \right]$ | (Eq.SC11c) |

where *k* is the gas exchange coefficient for CO_2_, *pCO_2_* is the partial pressure of CO_2_ in the surface water, *kH* is Henry's constant for CO_2_ at a given temperature, and [*CO_2_* ] is the concentration of CO_2_ in water at equilibrium with the overlying atmosphere. Flux was expressed as CO_2_ evasion (g C m^-2^ yr^-1^) and where relevant also standardized for remaining cotton strip mass (i.e., g C m^-2^ yr^-1^ per g dry mass).

### C.1.5 Carbon efflux from the Maiandros flumes system

Our calculations of carbon efflux from the flumes experiments followed the same procedure as above (Eq.SC1-11), but we did use specific dimensions and properties from the Maiandros flumes system to parameterize the model estimating *k*_600_ following Raymond et al. (2012). We used this model so the estimates of carbon efflux from the Maiandros flumes system were as similar as possible to those estimated from the field survey. Thus, for the respiration data from the flumes experiments, we used Eq.SC12 (see also Eq.SC3 above) with constant parameters based off the measurements reported by Mangold (2015):

| $k_{600}=4725 \times\left( VS \right)^{0.86} \times Q^{-0.14} \times D^{0.66}$ | (Eq.SC12) |
| --- | --- |

where *k*_600_ (m d^-1^) is the gas exchange velocity based on stream velocity (*V*, m s^-1^), slope (*S*; unitless), depth (*D*, m) and discharge (*Q*, m^3^ s ^-1^). For our calculations we used *V* = 0.09 m s^-1^ (0.05 – 0.15 m s^-1^ range), *S* = 0.001, *Q* = 2.778 × 10^-4^ m3 s^-1^, and *D* = 0.085 m.

**C.1.6 Potential influence of WW on carbon efflux from Swiss streams and rivers**

*Relative change approach* − We used our estimates of carbon efflux to predict the potential impact of WW inputs to streams and rivers on carbon budgets at the Swiss national scale. For a top-down approach based on the relative change in carbon efflux due to WW inputs, we used the estimate of 1.8 ± 0.25 Pg C yr^-1^ from streams and rivers globally (Raymond et al. 2013) as a starting point. We expected that only a small fraction of this global contribution would be prone to wastewater impacts in Switzerland, so we accounted for the land surface area of small-medium sized streams and rivers affected by inputs of WW in Switzerland (Strahler stream order 1-6; see below for further details). We used this size range because it matched closely with the sizes of our study sites (Burdon et al. 2019). To estimate the regional impact of WW we used the median % increase in carbon efflux (kg C m^-2^ yr^-1^) at wastewater-impacted sampling locations in the field survey (38.5%; -18.8 – 314.3%, 95% CI). Our estimates of carbon efflux were based on respiration rates from the cotton strips incubated in the field (see Sections C.1.3 and C.1.4 above). Thus, we used Eq.SC13 to calculate an estimate of the potential increase in carbon emissions from small-medium wastewater-impacted streams and rivers in Switzerland:

| $\Delta CE_{ww}^{1}=GCE\times\left( {{RCH}_{ww}}/{GSR} \right)\times\Delta RCE_{ww}$ | (Eq.SC13) |
| --- | --- |

where $\Delta CE_{ww}^{1}$ is the predicted increase in carbon emissions from wastewater-impacted streams and rivers across Switzerland, *GCE* is the global estimate for CO_2_ outgassing by streams and rivers annually (1800 ± 250 Tg C yr^-1^; Raymond et al. 2013), ${RCH}_{ww}$ is the land surface area for small−medium sized streams and rivers impacted by inputs of wastewater in Switzerland (33.12 km^2^), *GSR* is the estimated global land cover for streams and rivers (773,000 ± 79,000 km^2^; Allen and Pavelsky 2018), and $\Delta RCE_{ww}$ is our median estimate of the increase in carbon efflux from streams and rivers due to inputs of wastewater (38.5%). $\Delta CE_{ww}^{1}$ did not differ considerably when$\Delta RCE_{ww}$ (38.4%) was based on mass-corrected values (i.e., kg C m^-2^ yr^-1^ g DM^-1^).

Our approach in Eq.SC13 yielded an estimate of 29.7 Gg C yr^-1^ (-21.6 – 249.6 Gg C yr^-1^, 95% CI). This figure compared favourably when using the estimate of Butman et al. (2016) for carbon efflux from streams and rivers across the conterminous USA (69 Tg C yr^-1^; range: 36–110 Tg C yr^-1^) as $GCE$ and a total conterminous US stream and river surface area of 40,600 km^2^ (Butman and Raymond 2011) for $GSR$, resulting in $\Delta CE_{ww}^{1}=$21.6 Gg C yr^-1^ (-23.0−185.3 Gg C yr^-1^).

*Absolute areal approach* − For a bottom-up approach we used our estimates of the absolute areal efflux of carbon at sampling locations (kg C m^-2^ yr^-1^) from the field survey in Eq.SC14:

| $\Delta CE_{ww}^{2}= {RCH}_{ww}\times\left( ACE_{D}- ACE_{U} \right)$ | (Eq.SC14) |
| --- | --- |

where $\Delta CE_{ww}^{2}$ is the predicted increase in carbon emissions from wastewater-impacted streams and rivers, ${RCH}_{ww}$ is the land area of small to medium sized streams and rivers impacted by inputs of wastewater in Switzerland (33.12 km^2^), $ACE_{D}$ is the median estimated total carbon flux from sampling location D below WW inputs in our field study (kg C m^-2^ yr^-1^), and $ACE_{U}$ is the median estimated total carbon flux from location U1 above the WW inputs (kg C m^-2^ yr^-1^).

*Area of rivers and streams affected by wastewater in Switzerland* − We calculated the land surface area of small-medium sized Swiss streams and rivers (Strahler orders 1-6) impacted by inputs of wastewater as 33.12 km^2^. The size of streams and rivers for different Strahler orders followed Munz et al. (2012). We used data for stream lengths and the fraction affected by WW as reported in Strahm et al. (2013). Stream width data was obtained at https://www.bafu.admin.ch/dam/bafu/de/dokumente/hydrologie/fachinfo-daten/auswertungen_zu_dengewaesserbreiten.pdf.download.pdf/auswertungen_zu_dengewaesserbreiten.pdf (Date accessed 28.6.2020).

### C.1.7 Temperature dependency of cotton-strip respiration and decomposition

As reported in the Main Text, we assessed the temperature dependency of cotton-strip respiration and decomposition (ecosystem functions, EF). We expressed the natural logarithm (ln) of the EF as a function of standardized temperature (1/kBT-1/kBT x̅) which centers the inverse temperature data around zero, to make the intercept of the model (ln[EF(T x̅)]) equal to the EF rate at standardized temperature, T x̅ (here T x̅ = 6.748°C = 279.9K). According to Perkins et al. (2012), this correction greatly reduces the correlation between the slope and the intercept and makes the intercept biologically more meaningful (i.e., gives information on metabolic rates at a standardized temperature, as opposed to inﬁnite temperature).

## C.2 Field survey methods

### C.2.1 Hydrogeomorphology

We recorded water depths and ﬂow velocities (FlowTracker Handheld ADV, Sontek/YSI, Inc., San Diego, CA, USA) at 10 equidistant points across three transects at sampling locations (also measuring wetted channel width) during base flow conditions in Autumn 2013 and 2014 (see Burdon et al. 2016 for further details). We calculated slopes from site location data in ArcGIS (Pro 2.3, Esri, Redlands, CA, USA).

### C.2.2 Water quality

We collected separate water samples for general water chemistry and MPs in 1-L glass bottles. Samples were stored on ice before refrigeration. We kept samples for general water chemistry at 4°C in the laboratory; analyses were performed within 24 h using standard methods described for the Swiss National River Monitoring and Survey Programme (NADUF; www.bafu.admin.ch/wasser/13462/14737/15108/15109). We froze samples for analyzing organic MPs at -20°C until processing; concentrations were determined using liquid chromatography–high resolution mass spectrometry (LC–HRMS) according to the procedure described in Munz et al. (2016). We filtered, acidified, and stored samples in plastic centrifuge tubes for HM analysis at the time of collection. We quantified concentrations of HMs using a high-resolution inductively coupled plasma mass spectrometer (HR-ICP-MS; Element2, Thermo, Switzerland). See Meylan et al. (2003) for method details.

We calculated wastewater quantity as the proportion of treated effluent in the receiving stream:

| $\%WW=\left. \frac{Q_{ww}}{Q_{347}} \right.\times100$ | (1) |
| --- | --- |

where Q_347_ is the stream discharge that is reached or exceeded 347 days per year averaged over 10 years (equivalent to 95% of the time) and Q_WW_ is the mean discharge of the WWTP (Table SA1).

**Table SC1** The general water quality parameters measured during the field campaigns 2013 and 2014.

| **Parameter group** | **Individual parameters** |
| --- | --- |
| General (summary) parameters | Alkalinity , electrical conductivity, H_4_SiO_4_, pH, total hardness, total suspended sediments (TSS) |
| Nutrients | DOC, NH_4_^+^,NO_2_^-^, NO_3_^-^, PO_4_^3-^, TOC, total N, total P |
| Major cations | Na^+^, K^+^, Ca^2+^, Mg^2+^ |
| Major anions | Cl^-^, SO_4_^2-^ |
| Heavy metals | Ag, Cd, Co, Cr, Cu, Fe, Mn, Ni, Pb, Zn |

**Table SC2** Sampling and analytics for micropollutants (after Munz et al. 2016; note that site numbers vary between the two publications because less sites are considered here). The site numbers correspond to Table SA1. For the individual organic micropollutants see Munz et al. (2016).

| **Year** | **Timepoints** | | **Sites** | | **Organic substances analysed** | **Analytics** |
| --- | --- | --- | --- | --- | --- | --- |
| **2013** | June 2013 | 12.06.2013 |  | 3, 17, 19 | 389 | Offline SPE;  LC-HRMS |
|  |  | 17.06.2013 |  | 10, 13, 15 |  |  |
|  |  | 19.06.2013 |  | 2, 8, 9, 18 |  |  |
|  |  | 24.06.2013 |  | 4, 7 |  |  |
|  | February 2014 | 04.02.2014 |  | 3, 17, 19 | 57 | Online SPE;  LC-HRMS |
|  |  | 05.02.2014 |  | 9, 10, 13, 15 |  |  |
|  |  | 18.02.2014 |  | 4, 7, 18 |  |  |
|  |  | 20.02.2014 |  | 2, 8 |  |  |
| **2014** | March 2014: | 11.03.2014 |  | 1, 5, 6, 12 | 57 +10 heavy metals | Online SPE;  LC-HRMS  HR-lCP-MS for heavy metals |
|  |  | 12.03.2014 |  | 20, 21 |  |  |
|  |  | 19.03.2014 |  | 11, 14, 16 |  |  |
|  | May 2014: | 05.05.2014 |  | 1, 5, 6, 12 | up, down: 389 effluent: 57 +10 heavy metals |  |
|  |  | 19.05.2014 |  | 11, 14, 16 |  |  |
|  |  | 12.05.2014 |  | 20, 21 |  |  |
|  | July 2014 | 30.06.2014 |  | 5, 6, 12 | 57 +10 heavy metals |  |
|  |  | 01.07.2014 |  | 20, 21 |  |  |
|  |  | 05.08.2014 |  | 1, 16 |  |  |
|  |  | 18.08.2014 |  | 11, 14 |  |  |
|  | September 2014 | 15.09.2014 |  | 1, 5, 6, 12 | 57 +10 heavy metals |  |
|  |  | 17.09.2014 |  | 20, 21 |  |  |
|  |  | 23.09.2014 |  | 11, 14, 16 |  |  |
|  |  |  |  |  |  |  |
|  | November 2014 | 05.11.2014 |  | 1, 5, 6, 12 | 57 +10 heavy metals |  |
|  |  | 26.11.2014 |  | 11, 14, 16 |  |  |
|  |  | 02.12.2014 |  | 20, 21 |  |  |
|  |  |  |  |  |  |  |
|  | January 2015 | 19.01.2015 |  | 20, 21 | 57 +10 heavy metals |  |
|  |  | 20.01.2015 |  | 14, 16 |  |  |
|  |  | 21.01.2015 |  | 1, 5, 6, 12 |  |  |
|  |  | 29.01.2015 |  | 11 |  |  |

### C.2.3 Toxic Units

We evaluated the toxic stress at each sampling location using the Toxic Unit (TU) approach which assumes toxic effects are additive (Sprague 1971). We calculated the TUs for each substance *i* (MPs and HMs) at each location and time point by dividing the measured concentrations *C_obs_* by their EC50 values (Eq. 2):

| ${TU}_{i}=\frac{C_{obs\_i}}{{EC}_{50\_i}}$ | (2) |
| --- | --- |

We calculated mixture toxicity by summing up all TUs at each location and time point (Eq. 3):

| $sumTU=\left. \sum_{i} {TU}_{i} \right.$ | (3) |
| --- | --- |

Further details are provided in Munz et al. (2016) and Burdon et al. (2019).

### C.2.4 DNA analyses

We extracted and sequenced an additional sample from location D at one stream (Sévery) as a technical replicate for our next-generation sequencing (NGS) samples. In addition to analyzing communities using NGS, we also used automated ribosomal intergenic spacer analysis (ARISA) as our initial microbial community fingerprinting method. PCR amplification of the bacterial 16S-ITS-23S rRNA gene from the total DNA was performed with the primer pairs 1406F and 23SR as described by Yannarell et al. (2003). Fungal ARISA targeted the 18S-ITS1-5.8S-ITS2-28S regions of fungal nuclear ribosomal DNA using the primer pairs of 2234C and 3126T (Yannarell et al. 2011). The 1406F (bacteria) and 2234C (fungi) primers were labeled with 6-carboxyfluorescein at the 5’ end. PCR primer pairs and thermal programs are listed in Table SC3. The PCR products were analyzed using an ABI PRISM 3700 DNA analyzer (Applied Biosystems, Foster City, CA) in Genescan mode at SinoGenoMax (Beijing, China). ARISA electropherograms were inspected with the Peak Scanner™ Software v1.0 (ThermoFisher). The minimum noise threshold was set at 50 fluorescent units of peak height after normalization of the sum of total fluorescence in each profile.

**Table SC3** PCR primer pairs and thermal programs used in this study.

| Microbial indicators | Sequence (5’-3’) of primer pairs | Thermal program | Reference |
| --- | --- | --- | --- |
| 16S rRNA sequencing for bacteria | 515F: GTGCCAGCMGCCGCGG  907R: CCGTCAATTCMTTTRAGTT | 2 min at 95°C, 25 cycles of 30 s at 95°C, 30 s at 55°C, and 45 s at 72°C, finally 10 min at 72°C | Tuan et al. (2014) |
| ITS1 sequencing for fungi | ITS1-F: CTTGGTCATTTAGAGGAAGTAA  ITS1-R: GCTGCGTTCTTCATCGATGC | 3 min at 95°C, 30 cycles of 30 s at 95°C, 45 s at 55°C, and 45 s at 72°C, finally 10 min at 72°C | Frykman et al. (2015) |
| Bacterial ARISA | 1406F: TGYACACACCGCCCGT  23SR: GGGTTBCCCCATTCRG | 2 min at 94˚C, 30 cycles of 35 s at 94˚C, 45 s at 55˚C for, 2 min at 72 ˚C, and a final extension at 72˚C for 2 min | (Yannarell et al. 2003, Bürgmann et al. 2011, Yannarell et al. 2011) |
| Fungal ARISA | 2234C: GTTTCCGTAGGTGAACCTGC  3126T: ATATGCTTAAGTTCAGCGGGT | 2 min at 94˚C, 30 cycles of 35 s at 94˚C, 45 s at 55˚C for, 2 min at 72 ˚C, and a final extension at 72˚C for 2 min | Yannarell et al. (2011) |

**Table SC4** Information on Next Generation Sequencing (NGS) data including the numbers of sequences in each library and sequences removed by quality filtering. Accession numbers for sequences (16S rRNA and ITS1) supplied to the National Center for Biotechnology Information (NCBI) Sequence Read Archive (SRA) are provided.

| **NGS** | **Property** | **Numbers** | **NCBI SRA** |
| --- | --- | --- | --- |
| 16S | valid_copy.fq | 412446 | PRJNA602393 |
|  | filtered.fa | 334028 |  |
|  | uniques.fa | 92993 |  |
|  | otus.fa | 1012 |  |
| ITS1 | seqs.fq | 451000 | PRJNA602412 |
|  | filtered.fa | 448447 |  |
|  | uniques.fa | 73328 |  |
|  | otus.fa | 2201 |  |

**Table SC5** OTU read counts across different samples.

| **Site name** | **Read counts (bacteria)** | **Read counts (fungi)** |
| --- | --- | --- |
| Colombier downstream | 10575 | 5959 |
| Colombier upstream | 22463 | 12145 |
| Duerten downstream | 12353 | 20088 |
| Duerten upstream | 20105 | 32744 |
| Herisau downstream | 23825 | 15061 |
| Herisau upstream | 22644 | 11968 |
| Hochdorf downstream | 14505 | 26068 |
| Hochdorf upstream | 10027 | 26535 |
| Hornussen downstream | 12772 | 33483 |
| Hornussen upstream | 24246 | 20858 |
| Kerneried downstream | 17532 | 42409 |
| Kerneried upstream | 20511 | 28322 |
| Messen downstream | 23477 | 16145 |
| Messen upstream | 18449 | 36145 |
| Romont downstream | 20001 | 14025 |
| Romont upstream | 16695 | 17352 |
| Rothenthurm downstream | 11061 | 10352 |
| Rothenthurm upstream | 13391 | 13110 |
| Severy downstream | 21507 | 24647 |
| Severy upstream | 14804 | 34287 |

## C.3 Description of experimental conditions

### C.3.1 Overview of Maiandros flumes experiments

**Table SC6** Dates of Maiandros flumes experiments where the cotton-strip assay (CSA) was applied (for CSA dates see Table 1, MT).

| Experiment | Treatment abbreviation | Treatment | Start date | End date | Experiment duration  (days) | CSA duration  (days) |
| --- | --- | --- | --- | --- | --- | --- |
| Exp.1 | 0% WW  15% WW  50% WW  85% WW | River water (control)  River mixed with treated WW (15%)  River + WW (50%)  River + WW (85%) | 04.08.2014 | 10.09.2014 | 37 | 13 |
| Exp.2 | River  Nutrients  MPs  MPs + Nutrients | River water (control)  River dosed with N and P  River + MP-mix.1  River + MP-mix.1 + N and P | 17.10.2014 | 21.11.2014 | 35 | 29 |
| Exp.3 | River  Control  MPs  MPs + Nutrients | River water (control)  River dosed with methanol (technical control)  River + MP-mix.2  River + MP-mix.2 + N and P | 13.04.2015 | 28.05.2015 | 45 | 35 |
| Exp.4 | River  50% WW | River water (control)  River water mixed with treated WW (50%) | 21.10.2015 | 10.11.2015 | 21 | 21 |

### C.3.1 Experiment 1

#### Assignment of treatments

**Table SC7** Distribution of the four treatments across the 16 Maiandros flumes during Exp.1. Control (C): river Glatt water, the remaining treatments (T) consists of a mix of Glatt water with a given percentage of treated wastewater (WW).

| Block | Channel | WW | Treatment |
| --- | --- | --- | --- |
| 1 | 1 | 15% | T |
| 1 | 2 | 0% | C |
| 1 | 3 | 85% | T |
| 1 | 4 | 50% | T |
| 2 | 5 | 85% | T |
| 2 | 6 | 15% | T |
| 2 | 7 | 50% | T |
| 2 | 8 | 0% | C |
| 3 | 9 | 50% | T |
| 3 | 10 | 0% | C |
| 3 | 11 | 15% | T |
| 3 | 12 | 85% | T |
| 4 | 13 | 0% | C |
| 4 | 14 | 85% | T |
| 4 | 15 | 50% | T |
| 4 | 16 | 15% | T |

#### Experimental conditions

During Exp.1, the pumps providing water to the buffer tanks did not always work properly. There were 3 periods of stagnant water in the flumes (Fig.SC1). Despite these problems, the flumes for each of the respective treatments were filled with water all of the time.


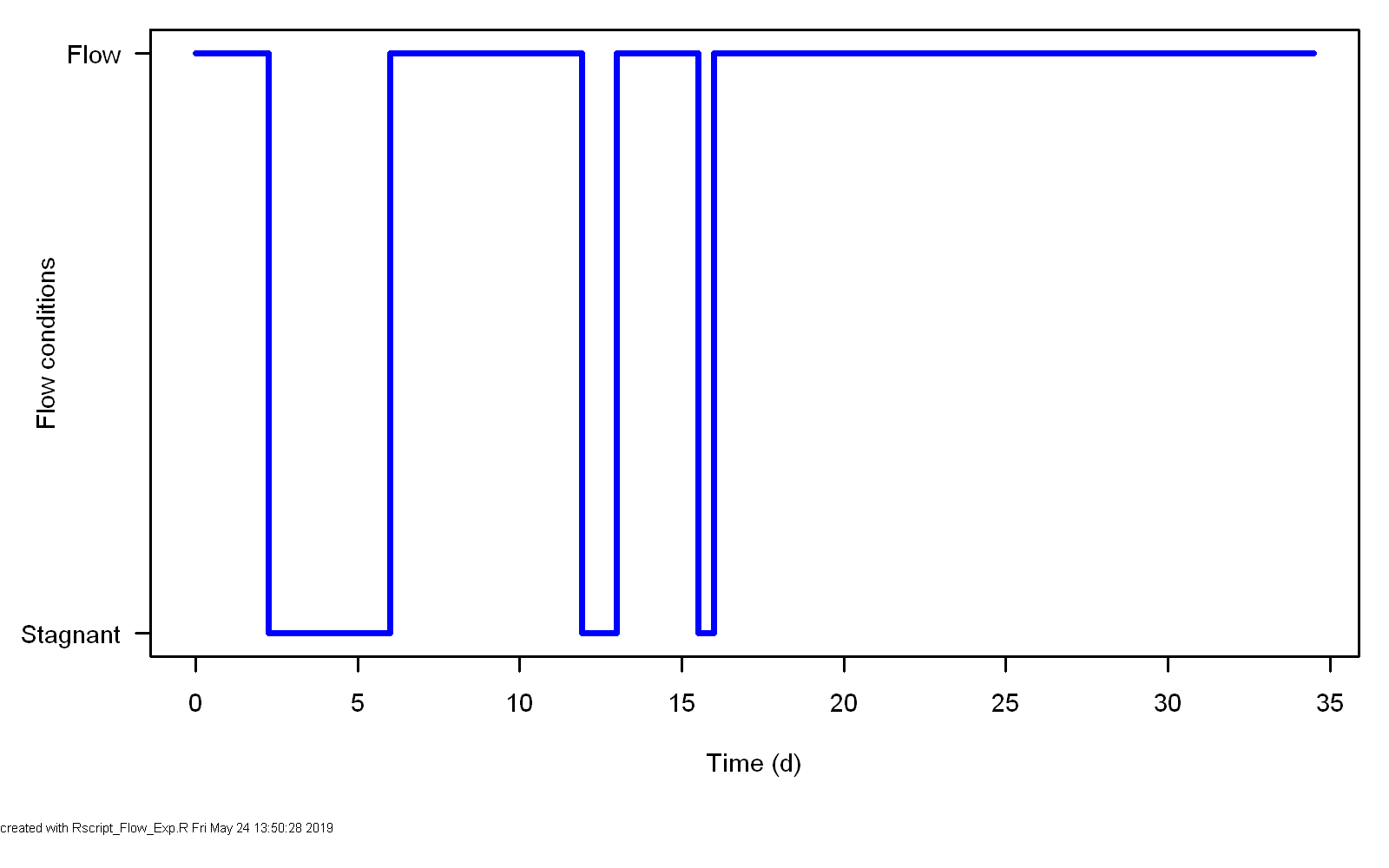


**Figure SC1** Flow conditions during Exp.1: *Flow* indicates flow-through conditions, while *Stagnant* represents periods of pump failures during which the water was stagnant in the flumes.

### C.3.2 Experiment 2

#### Assignment of treatments

**Table SC8** Distribution of the four treatments across the 16 Maiandros flumes during Exp.2. Control: river Glatt water, Nutrients: dosing of N and P, MPs: dosing of selected micropollutants, Nutrients + MPs: dosing of the same levels of nutrients and MPs as in the respective individual treatments.


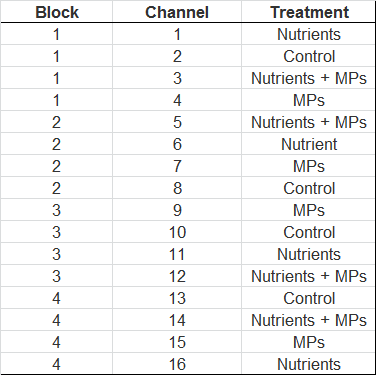


#### Experimental conditions

The flow rates had to be adjusted daily as they regularly decreased within 24 hours. Sometimes flow rates decreased by 40-60% within a day without an obvious cause (Fig.SC2).

Additionally, two Glatt pump failures caused complete interruptions of flows during the second half of the experiment (Fig.SC2). Due to the buffer tanks (BFs), the flow into the channels did not stop immediately, but slowly decreased as the hydrostatic pressure was reduced in the BF.

After the pump breakdowns the dosing was turned off, but not immediately. A decrease in flow of Glatt water combined with a stable pump rate of the HPLC pumps meant increased concentrations of the spiked compounds in the respective treatments. This could be easily detected by conductivity measurements. During the hydraulic target state the nutrient salts in the N:P treatments caused a conductivity increase of 3 to 4 μS cm^-1^ compared to the Glatt control treatment. After the Glatt pump failures with flow interruption, conductivity increases of 20 to 30 μS cm^-1^ were measured in the N:P treatments. Hypothetically, this was 7-8 times higher concentrations of the dosed substances than during the hydraulic target state.

After the Glatt pump failures, a new pump was installed which was not equipped with a filter on the uptake hose, meaning coarse organic matter (e.g., leaves) were pumped into the BT. After <3 days the screen in the BT was completely blocked allowing leaves to enter the flumes system and block the valves. This lead to highly-reduced flow rates and hence increased dosing levels.


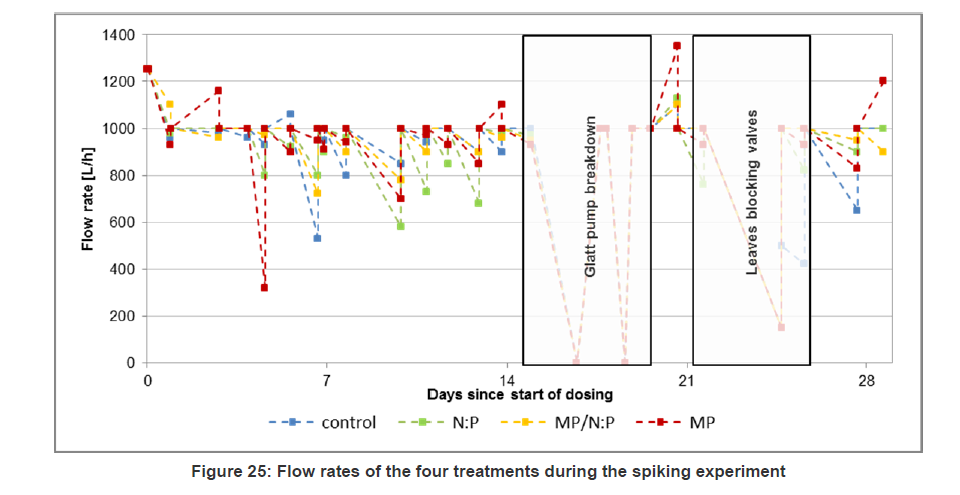


**Figure SC2** Flow rates through the distribution units of the four treatments over the course of Exp.2.

#### Dosing

We stored stock solutions at 4°C and continuously dosed at a rate of 0.65 ml/min for the entire experiment duration(s) using peristaltic pumps.

The compounds used for dosing and their target concentrations are listed in Table SC9. Two stock solutions were necessary for providing dosing of two treatments (MPs only and MPs + nutrients). The composition of these two solutions agreed well within the analytical uncertainty (Fig.SC3).


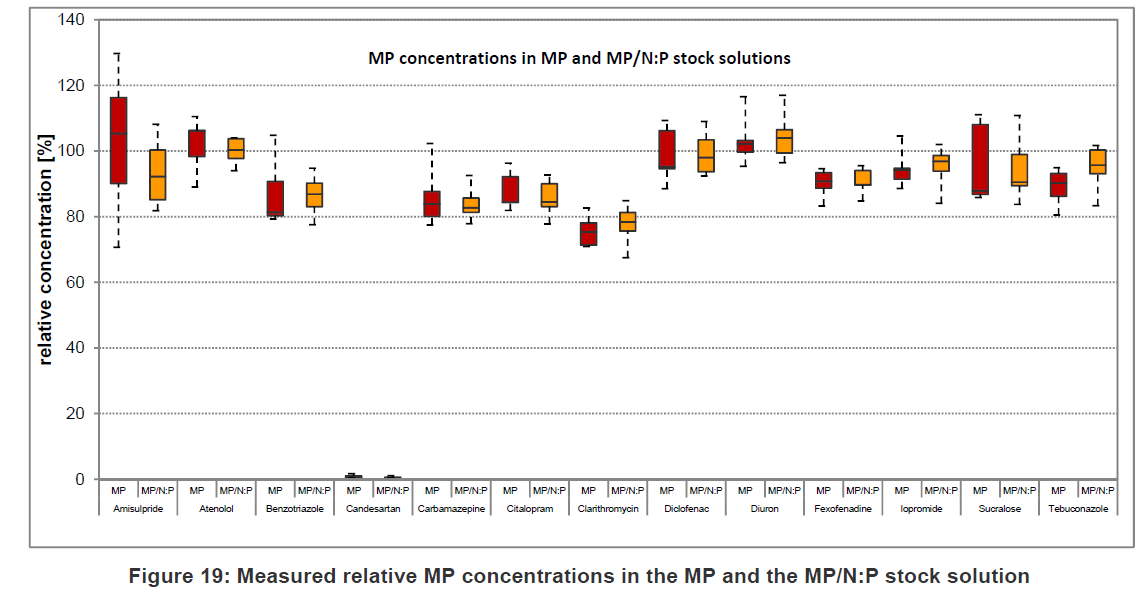


**Figure SC3** Comparison of relative concentrations of 13 out of 17 organic MPs in the stock solutions for the MP and the MP+nutrient treatment. Diazinone, β-estradiol, and metformin were excluded from the comparison for analytical reasons.

The stock solutions used for dosing in Exp.2 were prepared in a 1:1 water:methanol (MeOH) mixture to dissolve substances with poor solubility like diazinon or diclofenac. One reason for this high MeOH ratio is the high quantities required and the handling of the stock solutions. They were prepared in volumes of 10 L each which meant that supporting methods like ultrasonic baths could not help because they are designed for smaller volumes. In addition, a high ratio of methanol prevents the organic MP from hydrolysis.

One advantage of the methanol in the stock solution at high concentrations is its anti-freeze properties. The temperatures at Fällanden can easily fall below 0 °C overnight in November, potentially causing flow interruptions within the thin capillary from the HPLC pump. With solutions containing 50% MeOH, the dosing was frost-proof in the observed temperature range.

In Exp.2 the MeOH concentrations in the flumes were approximately 5.76 mg/L. The no-observed-effect-concentration (NOEC) of MeOH to freshwater ecosystems has been reported as 23.75 mg/L for chronic exposure (Kaviraj et al. 2004), meaning the dosed MeOH concentrations were unlikely to have caused toxic effects. However, MeOH is a labile carbon source. The 5.76 mg/L MeOH corresponded to an increased DOC of around 6 mg/L. This likely contributed to the growth of microbial biofilms in the flumes which affected the CSA.

The stock solutions were prepared in a 10 L beaker on a magnetic stirrer. For the combined treatment with MPs and nutrients, the nutrient salts were dissolved in nanopure water first, then the MeOH was added and at the very end the MP standards were pipetted. The ready-made solution was filled into a canister which was transported to the plant, where the solution was filled into the receiver tank. For the N:P or MP only treatment the unnecessary steps were skipped.

In the combined MP+N:P treatment it was impossible to add zinc because it precipitated with phosphate. Even in low concentrations, zinc and phosphate cannot be in solution together at a moderate pH. For the aimed concentrations in the stock solution pH would have had to be adjusted to a value below 2 according to the program Visual MINTEQ. For this treatment we used an additional dosing system only for the zinc.

**Table SC9** List of the dosed compounds (micropollutants, nutrients) and their concentration in the stock solution used for dosing and the respective target concentrations in the flumes.


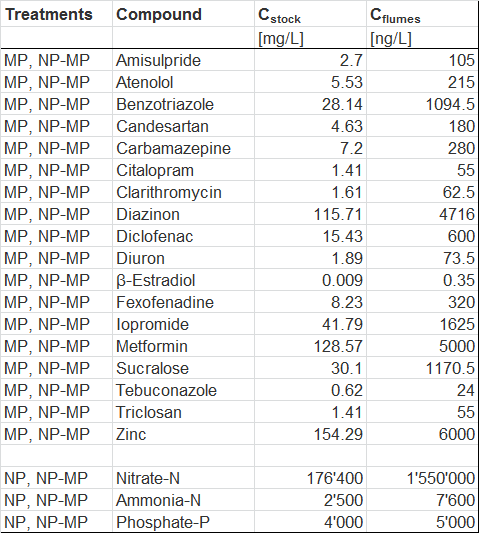


#### Sampling protocols (Exp.1&2)

Because only two water sources were used in Exp.1, sampling was limited to one sample for Glatt upstream water and one WW sample for HMs and MPs, which were time-consuming to analyse. The Glatt upstream sample was taken from the BT in contrast to the WW sample, which we took from a 24-hour mixed sample provided by the WWTP. To calculate back the dilutions which were actually achieved in the channels we took samples for general water chemistry from the four different treatments directly from one channel per treatments and from the two BTs.

**Table SC10** Sampling schemes for water quality observations for the Maiandros experiments Exp.1 and Exp.2. AUA: abbreviation for samples of general water chemistry, BT Glatt upstream: buffer tank for the Glatt river water, BT WW: buffer tank for the treated wastewater, WW_24h, mixed_: Mixed, time-proportional sample from the WWTP outlet.


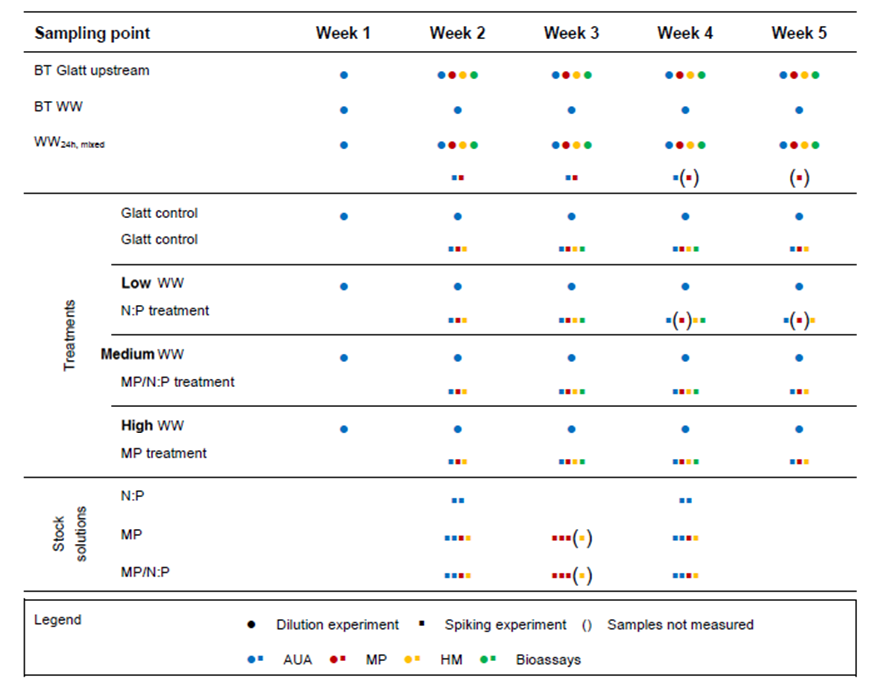


### C.3.3 Experiment 3

#### Assignment of treatments

**Table SC11** Distribution of the four treatments across the 16 Maiandros flumes during Exp.3. Control: river Glatt water, MeOH: technical control corresponding to the Glatt river water dosed with the same level of methanol as in the MP treatment, MP: Glatt water dosed with 17 selected micropollutants, MP+N:P: same as MP with the additional dosing of nutrients (N, P).


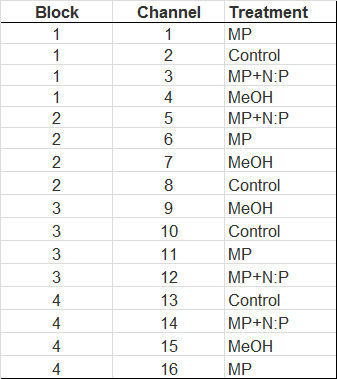


#### Experimental conditions

During Exp.3, flow was stable and varied little around the target flow rate (see Fig.SC4). The same holds true for temperature, dissolved oxygen and conductivity. All of these quantities varied very little among the 16 channels as seen from the small coefficients of variation (Fig.SC5).

Nevertheless, a few technical issues occurred that are listed below (Table SC12).

**Table SC12** List of technical problems that occurred during Exp. 3.


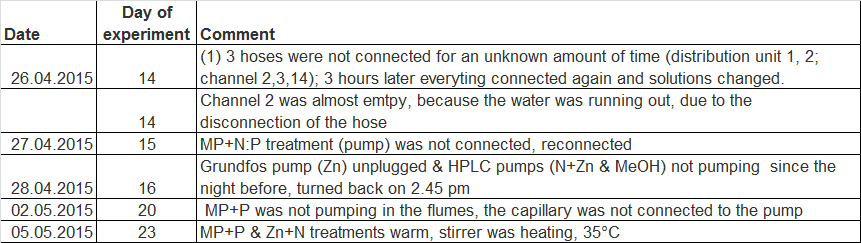


#### Dosing

The stock solutions used for dosing in Exp.3 were prepared in an 8:10000 water to methanol (MeOH) mixture. This greatly reduced the MeOH concentrations in the flumes compared to Exp.2. The DOC-equivalents of methanol in Exp. 3 corresponded to approximately 0.04 mg/L. This dosing had a minimal effect on the background DOC values of about 3 mg/L (see Fig.SD21).


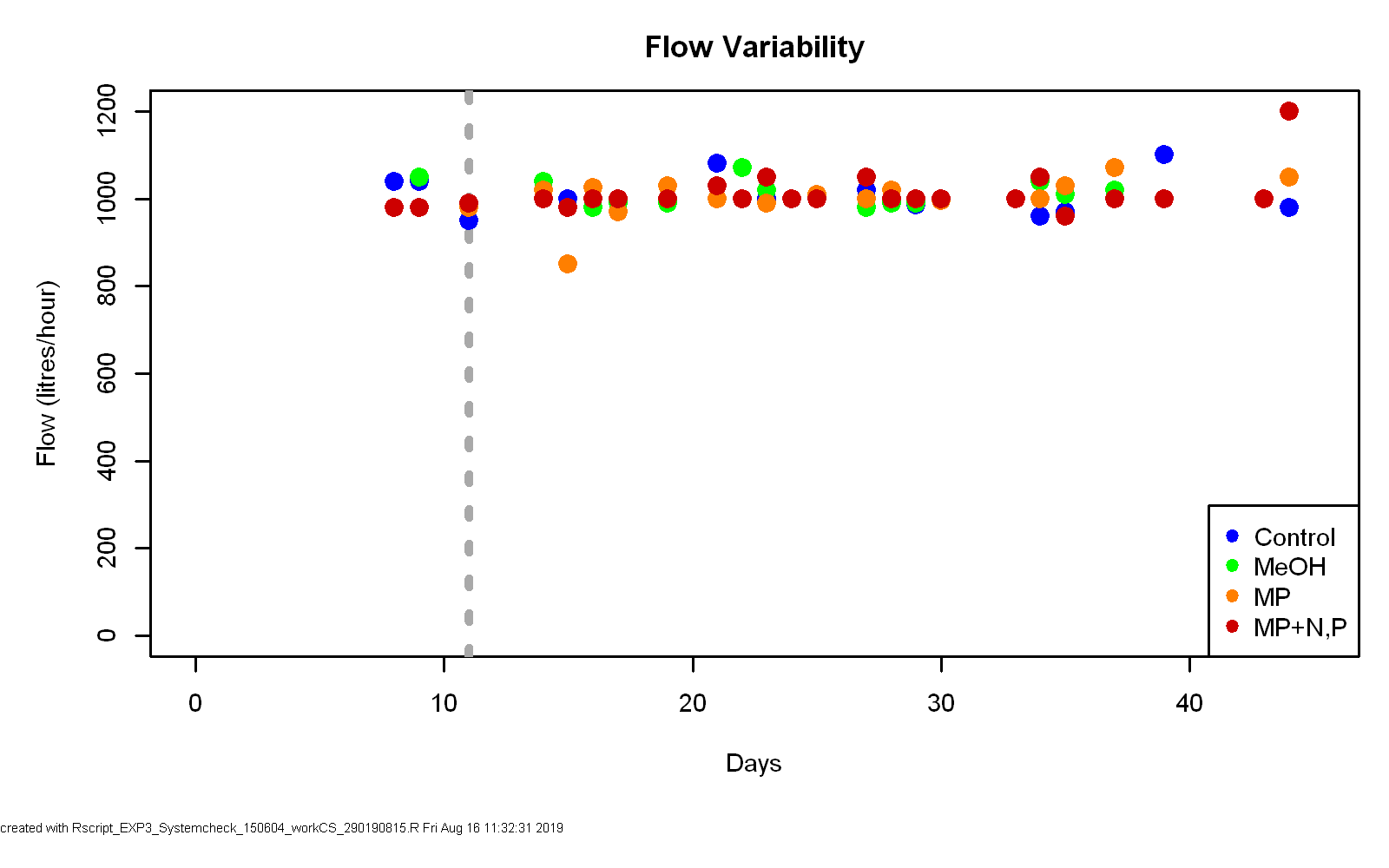


**Figure SC4** Overview about the flow conditions during Exp.3. The dashed vertical line indicates the start of dosing.


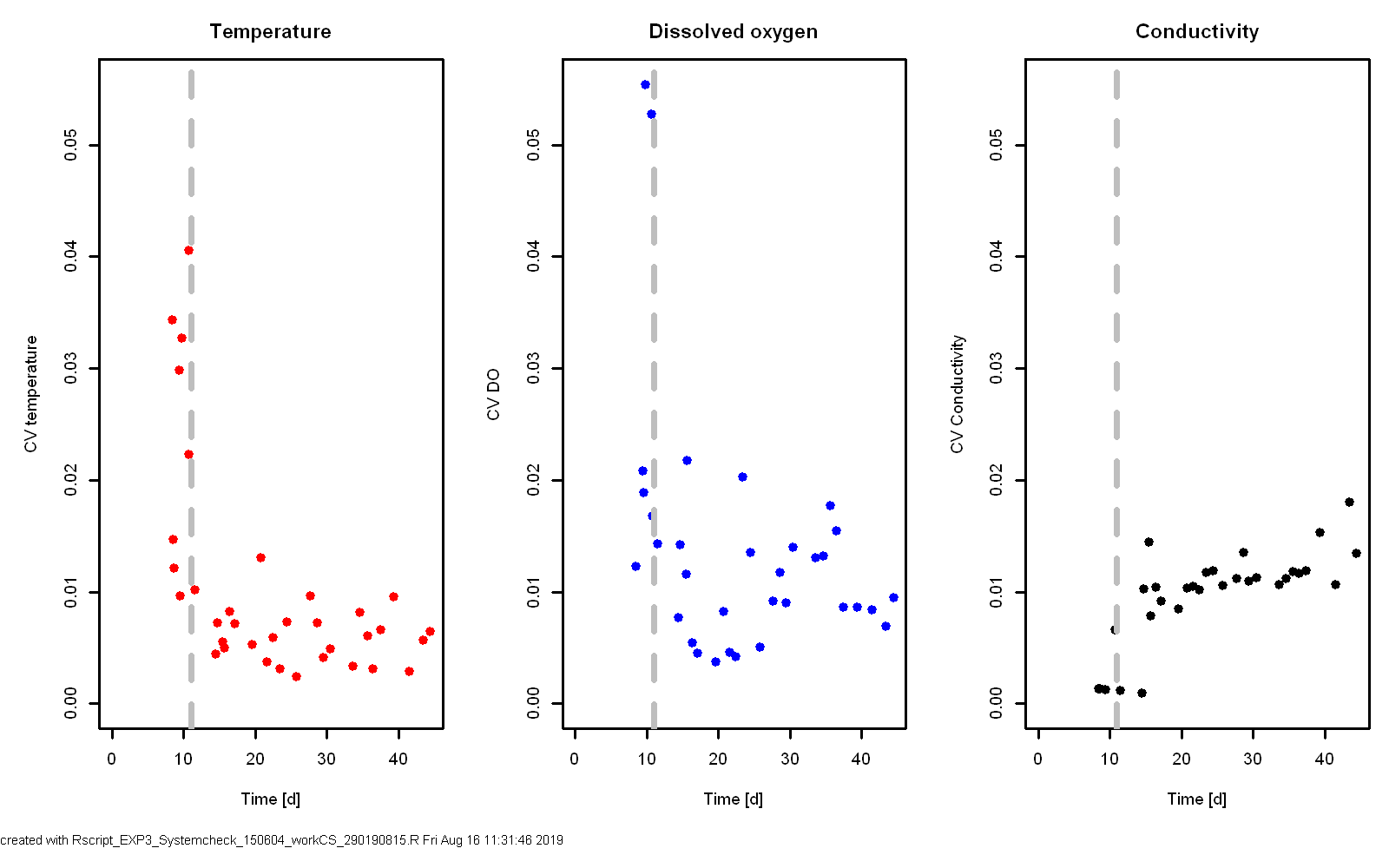


**Figure SC5** Coefficients of variation of water temperature, dissolved oxygen, and electrical conductivity between the 16 channels during Exp.3. The dashed vertical lines indicate the start of dosing.

### C.3.4 Experiment 4

For the membrane-based mesocosms we used reconstituted cellulose (RC) dialysis membranes (Spectra/Por, Spectrum Europe, Breda, Netherlands), with a molecular weight cut-off of 3.5 kDa. We made the bags from extruded tubes (34 mm diameter) sealed by two nylon clips (Fig.SC6). Before the experiment, we hydrated membrane bags by rinsing with MQ water three times (approx. 30 min each rinse).

The cellulose membranes allow efficient exchange of compounds with molecular weight <3.5 kDa between the internal and external environment. These compounds include mineral nutrients and gases essential for microbial growth, while allowing the products of metabolism to equilibrate with the external environment (Pomati and Nizzetto 2013). RC membrane are highly hydrophilic, a property that strongly reduces interactions with hydrophobic components in the water (e.g., cell membranes, dissolved organic carbon, hydrophobic chemicals), thus limiting the growth of biofilms (Pomati and Nizzetto 2013).


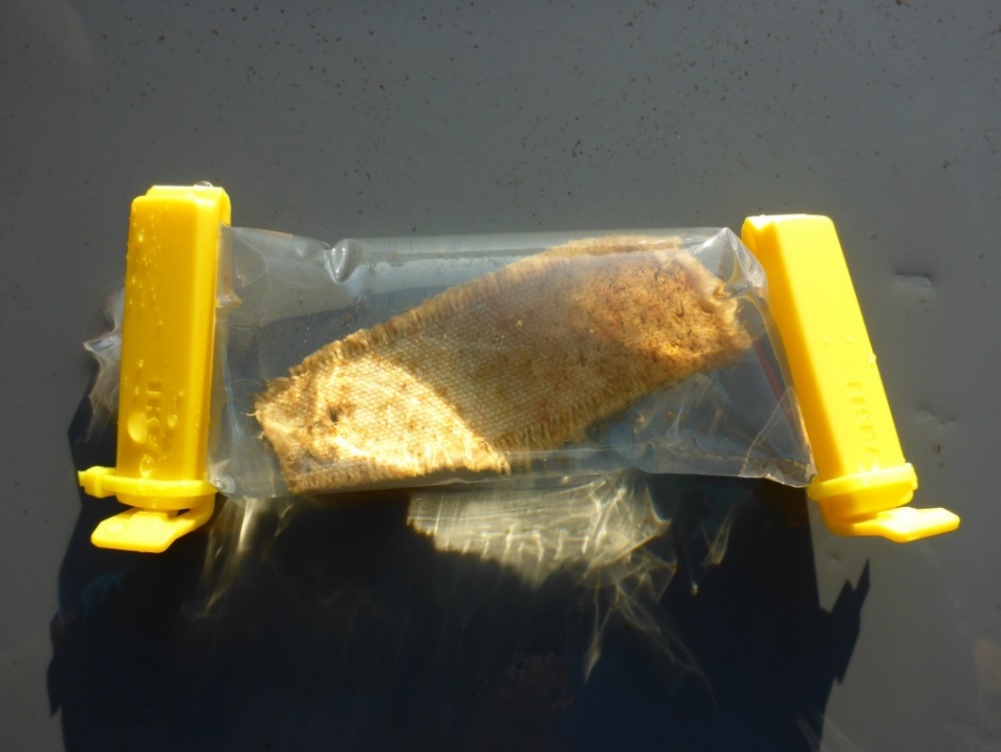


**Figure SC6** One of the membrane-based mesocosms used in the cotton-strip transplant experiment (Exp.4).

### C.3.5 Laboratory experiment

We used plastic cups in the laboratory experiment. New 185 ml plastic cups were filled with MQ water and exposed to natural UV for five days, then rinsed thoroughly three times and dried prior to the experiment. The plastic cups were arranged in each tray following a stratified-random approach (i.e., so treatments were evenly spread across trays). We covered each block of cups with boxing to help minimize evaporation.

From each sampling location, we filtered (GF/F, 47 mm Ø, 0.7 μm pore size, Whatman plc, Maidstone, UK) and then sterilized 2-l of stream water with UV light. Following Canonica et al. (2008), UV disinfection used a Haereus Noble light model TNN 15/32 low-pressure mercury arc lamp (nominal power 15 W, main emission line in the UV-C, λ=254 nm).

## C.4 Data analysis

### C.4.1 Microbial community analyses

*Diversity* - To avoid discarding 16S rRNA data following correction for singletons (Chiu and Chao 2016), we used sample-size-based rarefaction and extrapolation (R/E) sampling curves for species richness and Shannon diversity that was extrapolated to 25000 OTUs, guided by an estimated asymptotic species diversity relationship using the iNEXT R package (Hsieh et al. 2016). The R/E curves for species richness are shown in Fig.SC7 below for each sample. Sample coverage values (0.986–0.997) for 16S rRNA samples indicated that sequencing depths were sufficient for reliable community analysis. Although we did not use rarefaction for our analyses using ITS data, we did check sample coverage (0.9854–0.9968) which suggested that the ITS data was adequate.


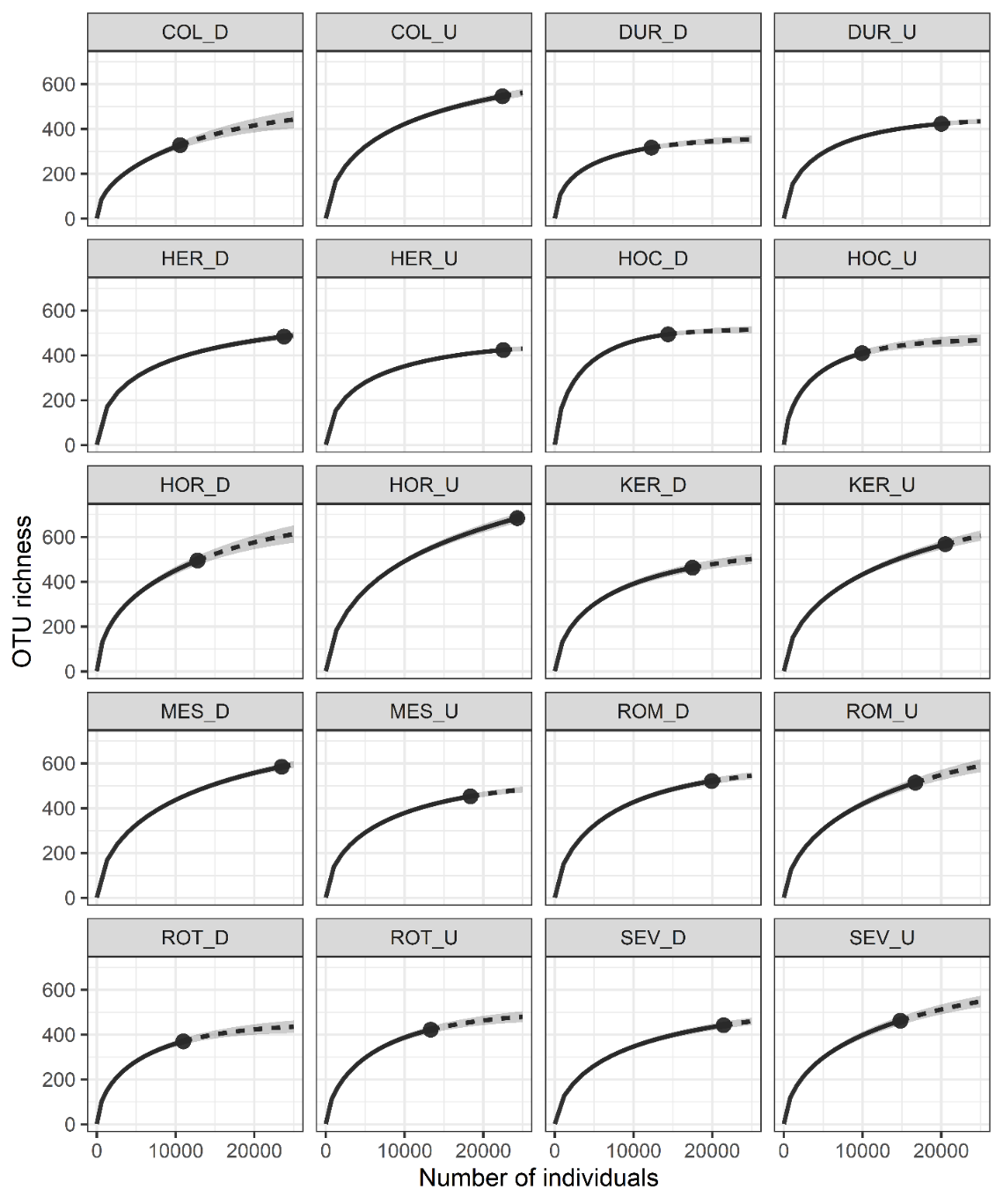


**Figure SC7** Sample-size-based R/E curves for bacterial OTU richness extrapolated to 25000 individuals. 95% confidence intervals are shown in grey.

*Mixed models –* As described in the Main Text, we used linear mixed effects models to test our *a priori* hypothesis that inputs of wastewater influence microbial communities, making use of our study design with sampling locations above and below WW inputs in streams across Switzerland. We also tested additional models to investigate what environmental factors significantly explain variation in our chosen microbial α and β diversity indicators (Table 2, Main Text). For predictor variables, we used the same environmental predictors used in the structural equation models: % WW and % arable land in the upstream catchment, concentrations of dissolved inorganic nitrogen and reactive phosphorus, and toxic units of fungicides and non-fungicides. We also included mean stream temperatures (°C). Sampling location was included in all initial models and “Site” was the random effect. To help normalize data and improve homoscedasticity, we log-transformed chemical and temperature data, and logit-transformed proportion data (e.g., % land cover of arable cropping and % WW). We standardized predictors using the ‘decostand’ function in R (i.e., centred on the column means and scaled by unit variance). Scaling makes regression coefficients of predictor variables measured in different units comparable and permits their use as measures of effect size (Schielzeth 2010). Potential input variables were selected with a forward-selection procedure using the the ‘forward.sel’ function in the “packfor” R function (v 0.0-8). Candidate models were fitted with restricted maximum likelihood (REML) and progressively reduced using likelihood ratio testing (LRT) as a weight of evidence approach. Our LRTs used the ‘anova’ function in the R package “lmerTest” with Satterthwaite's approximation to degrees of freedom. LRTs have been shown to be anti-conservative and sensitive to sample size, with Type 1 error rates increasing with decreasing sample size, but Satterthwaite's approximation has been found to mitigate this problem (Luke 2017). Our objective was to determine the significant environmental predictors and not to generate models for forecasting. We tested raw counts of fungi (ITS) OTUs using a generalised linear mixed model with the ‘glmer’ function assuming a Poisson distribution (log-link function); iterations of the model for fungal richness included random intercepts and random slopes models compared using Bayesian Information Criterion (BIC) scores. All other models relied on the ‘lmer’ and ‘blmer’ functions. We computed LME models with the R packages “lmer4” (v. 1.1-21) or “blme” (v. 1.0-4). Parameter estimates, 95% confidence intervals, p-values, and marginal and conditional *R^2^* values are reported in Tables SD7−8.

*Constrained ordination and variation partitioning –* Similar to Burdon et al. (2019), we used constrained ordination techniques to test environmental and spatial influences on microbial community composition (Table SD9). The data was Hellinger-transformed and used the Euclidean-distance metric (Legendre and Gallagher 2001). We tested presence-absence (occupancy) data for bacterial and fungal communities and relative abundance data for bacteria (16S rRNA). For predictor variables, we used the same environmental predictors used in the structural equation models: % WW and % arable land in the upstream catchment, concentrations of dissolved inorganic nitrogen and reactive phosphorus, and toxic units of fungicides and non-fungicides. We also included mean stream temperatures (°C). To help normalize data and improve homoscedasticity, we log-transformed chemical and temperature data, and logit-transformed proportion data (e.g., % land cover of arable cropping and % WW). Data was then standardized using the ‘decostand’ function in R (i.e., centred on the column means and scaled by unit variance).

To select model predictors and avoid over-parametrising models, we used three approaches:

1. In our first approach, we used a forward-selection procedure to select a subset of explanatory variables following the method recommended by Blanchet et al. (2008). This selection procedure is performed in two steps to control for the probability of a Type I error and overestimation of the explained variance. To prevent overestimation of the explained variance, the forward selection has to be carried out with two stopping criteria: (i) the usual alpha significance level (α = 0.05) and (ii) the adjusted coefficient of multiple determination (*R^2^_adj_*) calculated using all potential explanatory variables. We used forward selection for both environmental and spatial predictors using the the ‘forward.sel’ function in the “packfor” R function (v 0.0-8).
2. Our second approach (“positive variation”) involved first individually testing each environmental predictor with our microbial community data. Only predictors that explained a positive fraction of total variation in the community matrix were retained for our final models. This is because negative adjusted *R*^2^ values correspond to cases where the explanatory variables explain less variation than random normal variables would, and thus are interpreted as zeros (Legendre 2008). Our positive variation approach was useful for selecting meaningful parameters when the forward-selection procedure did not indicate them as significant explanatory variables.
3. The positive variation approach allowed us to use a third approach (i.e., combined) to test environmental predictors selected with our positive variation approach with spatial predictors selected using forward selection.

For some of the positive variation models, we combined environmental predictors according to *a priori* hypotheses (i.e., to test the independent influence of MPs) (Burdon et al. 2019). As a final test to ensure that models were not over-parameterised, we used the ‘vif.cca’ function to obtain variance inflation factors. In our combined and positive variation models for bacterial presence-absence data, arable land cover was excluded because it had a VIF score >4 and was correlated with dissolved inorganic nitrogen.

Spatial structuring of communities using Cartesian coordinates was assessed using Principal Coordinates of Neighbours Matrices (PCNM) analysis (Borcard and Legendre 2002). PCNM descriptors (or axes) represent a spectral decomposition of the spatial relationships among the study sites (Borcard and Legendre 2002). This can be important where processes structure communities at different spatial scales (Borcard et al. 2004). PCNM geographic functions are a type of ‘distance-based eigenvector maps’ (DBEMs), which belong to a general class called ‘Moran's eigenvector maps’ (MEMs) (Dray et al. 2006). Only one PCNM axis (PCNM5) was selected in our forward selection approach for all community data matrices.

For these analyses, we used the “vegan” R package (v. 2.5-6) with functions ‘pcnm’, ‘rda’, and ‘varpart’. The significance of each independent variation component was permutation-tested using 1000 randomizations (Peres-Neto et al. 2003). Results were visualised using the “venneuler” R package (Table SD9). We also visualised the results of partial redundancy analyses (for bacterial and fungal occupancy data) using our combined modelling approach after conditioning out the influence of the spatial predictor (PCNM5) – see Fig.SD6.

*Microbes and ecosystem processes* - We also used Hellinger-transformed community data with redundancy analysis (RDA) models to test the association of ecosystem processes (tensile strength loss individually and in conjunction with mass loss and respiration with microbial community composition. The significance of tensile-strength loss was permutation-tested using 1000 randomizations (Peres-Neto et al. 2003) and is reported in the Main Text.

*Beta-diversity partitioning -* Following Baselga (2010), we sought to disentangle the contribution of spatial turnover (species replacements) and nestedness (species losses) to β-diversity patterns. This procedure uses additive partitioning to separate the components of spatial turnover and nestedness that underlie total β-diversity (Baselga 2010). Pairwise estimates of dissimilarity between sampling locations were obtained using ARISA occupancy data. Sørenson´s, Simpson´s (turnover), and nestedness measures (i.e., indices) were calculated for each site using the “betapart” package in R (Baselga and Orme 2012). For the ARISA data, differences in pairwise measures of dissimilarity between the downstream site (D-U1) and the upstream controls (U1-U2) were then tested using LMM (i.e., with “Site” as a random effect).

### C.4.2 Structural equation modelling (SEM)

The SEM testing the response of mass-loss rates (*k*_TD_) at all 20 sites included two ultimate pressures (% WW and % arable land in the upstream catchment), and four proximate stressors (concentrations of dissolved inorganic nitrogen and reactive phosphorus, and toxic units of fungicides and non-fungicides). Sampling location was considered as a fixed categorical predictor. Data was standardized using the ‘decostand’ function in R (i.e., centred on the column means and scaled by unit variance).To avoid over-specifying the SEMs and to identify important predictors explaining variation in temperature-corrected rates of cotton-strip tensile-strength loss, respiration, and mass loss, we used a forward-selection procedure with the ‘forward.sel’ function in the “packfor” R function.

### C.4.3 Linear mixed-effects modelling (LME)

For the laboratory experiment results, the initial model testing respiration included mean temperature and assay duration (time) as covariates, but a likelihood ratio test indicated that temperature did not have a significant influence in the model so we discarded it from further statistical tests.

The “blmer” function in R uses a partial Bayesian method to produce maximum *a posteriori* (MAP) estimates using regularizing priors to force the estimated random-effects variance-covariance matrices away from singularity (Chung et al. 2013). The “blmer” function does not provide *P*-values so we used likelihood ratio tests to test for statistical significance.

# Appendix D: Additional results

## D.1 Field survey results

### D.1.1 Concentrations of MPs and nutrients

**Table SD1** Average concentrations (μg/L) of different classes of MPs at the upstream and downstream locations across all 24 EcoImpact sites. Locations D were impacted by a WWTP discharge. Other: includes household chemicals. Only concentrations > LOQ have been considered.

| **MP class** | **EcoImpact 2013** | | | **EcoImpact 2014** | | |
| --- | --- | --- | --- | --- | --- | --- |
|  | **U1** | **Effluent** | **D** | **U1** | **Effluent** | **D** |
| Fungicide | 0.006 | 0.038 | 0.020 | 0.014 | 0.043 | 0.015 |
| Herbicide | 0.017 | 0.020 | 0.030 | 0.011 | 0.047 | 0.020 |
| Insecticide | 0.006 | 0.011 | 0.013 | 0.003 | 0.020 | 0.007 |
| Other | 0.033 | 1.721 | 0.119 | 0.020 | 1.765 | 0.282 |
| Pharmaceutical | 0.016 | 0.570 | 0.038 | 0.007 | 0.431 | 0.072 |
| Metals | na | na | na | 1.046 | 3.971 | 1.704 |

**Table SD2** Maximum concentrations (μg/L) of different classes of MPs at the upstream and downstream locations across all 24 EcoImpact sites. Locations D were impacted by a WWTP discharge. Other: includes household chemicals. Only concentrations > LOQ have been considered.

| **MP class** | **EcoImpact 2013** | | | **EcoImpact 2014** | | |
| --- | --- | --- | --- | --- | --- | --- |
|  | **U1** | **Effluent** | **D** | **U1** | **Effluent** | **D** |
| Fungicide | 0.11 | 0.33 | 0.73 | 0.55 | 4.40 | 1.80 |
| Herbicide | 0.43 | 0.13 | 3.00 | 0.41 | 0.96 | 2.40 |
| Insecticide | 0.02 | 0.03 | 0.13 | 0.02 | 0.44 | 0.11 |
| Other | 0.48 | 8.00 | 1.10 | 0.52 | 10.00 | 3.50 |
| Pharmaceutical | 0.36 | 10.00 | 0.83 | 0.43 | 8.00 | 2.00 |
| Metals | na | na | na | 25.81 | 122.6 | 59.07 |

**Table SD3** Concentrations of nutrients at the upstream (U1, U2) and downstream (D) locations across all 24 EcoImpact sites. Locations D were impacted by a WWTP discharge. DIN, dissolved inorganic nitrogen; SRP, soluble reactive phosphorus.

| **Nutrient** | **Unit** | **Statistic** | **All years** | | | **2013** | | | **2014** | | | | |  |
| --- | --- | --- | --- | --- | --- | --- | --- | --- | --- | --- | --- | --- | --- | --- |
|  |  |  | **U2** | **U1** | **D** | **U2** | **U1** | **D** | **U2** | **U1** | | **D** | |  |
| DIN | mg/l | Mean | 3.0 | 3.1 | 4.8 | 3.2 | 3.2 | 4.7 | 2.9 | | 2.9 | | 4.9 | |
|  |  | Median | 3.1 | 3.1 | 5.0 | 3.2 | 3.3 | 4.4 | 2.9 | | 2.9 | | 5.1 | |
|  |  | Max. | 6.4 | 6.5 | 7.7 | 6.4 | 6.5 | 7.7 | 4.7 | | 4.7 | | 6.8 | |
|  |  | Min. | 0.8 | 0.9 | 1.1 | 0.8 | 0.9 | 1.1 | 0.9 | | 0.9 | | 2.7 | |
| SRP | μg/l | Mean | 17.5 | 18.1 | 78.1 | 22.3 | 21.7 | 64.0 | 12.7 | | 14.5 | | 92.2 | |
|  |  | Median | 14.6 | 15.4 | 49.1 | 18.1 | 19.1 | 44.0 | 7.6 | | 10.1 | | 59.5 | |
|  |  | Max. | 50.9 | 48.1 | 322.8 | 50.9 | 48.1 | 205.7 | 41.7 | | 46.2 | | 322.8 | |
|  |  | Min. | 4.2 | 5.0 | 21.0 | 6.5 | 5.0 | 21.0 | 4.2 | | 5.7 | | 28.3 | |

### D.1.2 Cotton-strip NGS

We described bacterial communities on cotton strips using 16S rRNA gene sequences. We obtained 92,993 sequences belonging to 998 operational taxonomic units (OTUs). Of these OTUs, 477 belonged to Proteobacteria, 280 to Bacteroidetes, 40 to Planctomycetes 35 to Acidobacteria, two to Nitrospirae, one to Deinococcus-Thermus, and 75 to unknown bacteria. The dominant orders were Cytophagales, Sphingobacteriales, Rhizobiales, and Flavobacteriales, whereas the common known bacteria genera were *Flavobacterium*, *Pseudomonas* and *Opitutus*. We described fungal communities on cotton strips with ITS1 gene data. We obtained 73,328 sequences belonging to 2201 OTUs. Of these OTUs, 579 belonged to Ascomycota, 306 to Basidiomycota, 10 to Chytridiomycota, and 1241 unknown fungi. The dominant orders were Pleosporales and Tremellales, whereas the common known fungal genera were *Mortierella* and *Dioszegia*. We show changes in composition among sampling locations in Figs.SD2-3.

Table SD4 reports results from our PERMANOVA models using the ‘adonis’ function in R. In our unconstrained ordinations, bacterial community dispersion did not differ between sampling locations for presence-absence (“betadisper”, *F*_1,18_ = 3.45, *P* = 0.09) or relative-abundance data (*F*_1,18_ = 0.93, *P* = 0.37). Fungal community dispersion did not differ significantly between sampling location (*F_1,18_* = 2.67, *P* = 0.14).

Tables SD7−8 report results from linear mixed models showing statistically significant influences of sampling location and environmental predictors on microbial α and β diversity indicators.

Similar to our approach in Burdon et al. (2019) with macroinvertebrate data, here we used constrained ordination techniques to test environmental and spatial influences on microbial community composition (Table SD9). Our modelling approach is described in full at C.4.1, but briefly here we used three approaches to specify models. Our first approach involved a forward-selection procedure recommended by Blanchet et al. (2008) to select both environmental and spatial predictors, whereas another approach involved selecting environmental predictors that explained positive variation in our community response matrices. We used a combined approach where the environmental predictors selected with positive variation were tested with the spatial predictor (PCNM5) selected by the Blanchet et al. (2008) approach.

We found evidence that TUs of non-fungicides significantly explained 1.54% variation in bacterial (16S rRNA) community composition by presence-absence (PA) after accounting for spatial location (Table SD9). Our positive variation approach included other environmental predictors (DIN, SRP, and %WW) in addition to non-fungicides (Fig.SD6), but their independent contribution was not significant in a combined model (Table SD9). We unable to discern a significant independent influence of non-fungicides from these environmental predictors.

Only the spatial predictor significantly explained variation in bacterial (16S rRNA) community composition by relative abundance (RA; Table SD9), but TUs of non-fungicides were again selected with our positive variation approach despite not having significant influence on bacterial relative abundances.

For fungal (16S rRNA) community composition by presence-absence (PA), we found that only the spatial predictor was selected by forward selection, but its independent contribution to variation explained was not significant when tested with the environmental predictors selected by positive variation (mean water temperatures and TUs of fungicides and non-fungicides; Fig. SD4). These two groups of MPs explained equivalent amounts of fungal community variation (~0.8%), so we combined them to show that synthetic chemicals explained more variation than temperature, although this independent influence was not significant (Table SD9). All three rda models for fungi were significant, suggesting that forward-selection may be overly conservative.

**Table SD4** Results from PERMANOVA models testing the marginal influence of location (U1, D) on microbial community composition after accounting for the upstream land cover by arable cropping.

| Community | Data | Predictor | Df | SS | MS | pseudo*-F* | *R^2^* | *P* |
| --- | --- | --- | --- | --- | --- | --- | --- | --- |
| Bacteria | PA | Arable cropping | 1 | 0.053 | 0.053 | 1.050 | 0.055 | 0.127 |
| (16S rRNA) |  | Location | 1 | 0.056 | 0.056 | 1.109 | 0.058 | **0.040** |
|  |  | Residuals | 17 | 0.852 | 0.050 | 0.887 |  |  |
|  |  | Total | 19 | 0.960 | 1.000 |  |  |  |
|  | RA | Arable cropping | 1 | 0.041 | 0.041 | 0.668 | 0.036 | 0.365 |
|  |  | Location | 1 | 0.051 | 0.051 | 0.828 | 0.045 | **0.025** |
|  |  | Residuals | 17 | 1.054 | 0.062 | 0.919 |  |  |
|  |  | Total | 19 | 1.146 | 1.000 |  |  |  |
| Fungi | PA | Arable cropping | 1 | 0.127 | 0.127 | 0.795 | 0.042 | 0.510 |
| (ITS) |  | Location | 1 | 0.157 | 0.157 | 0.981 | 0.052 | **0.018** |
|  |  | Residuals | 17 | 2.722 | 0.160 | 0.905 |  |  |
|  |  | Total | 19 | 3.006 | 1.000 |  |  |  |

**
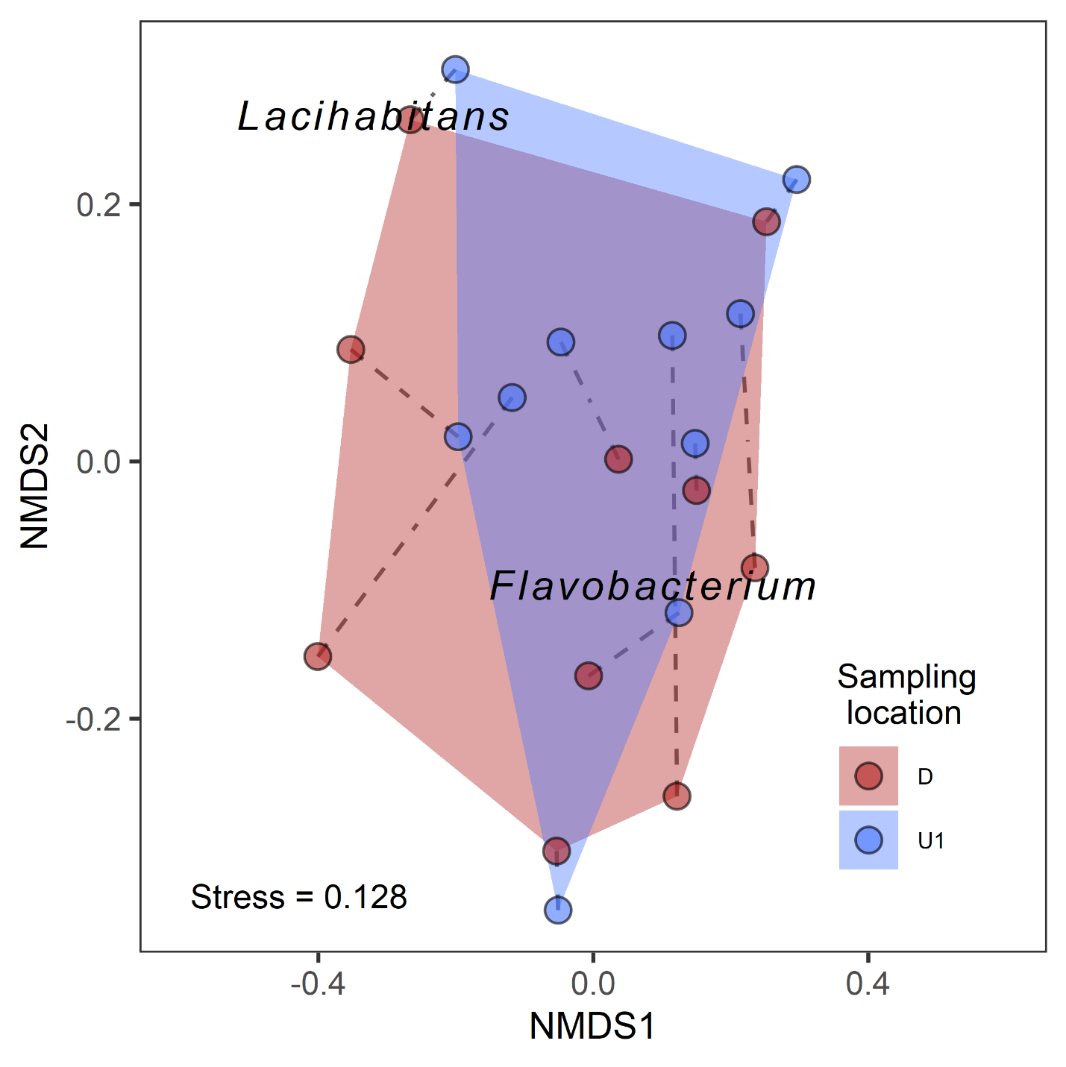
**

**Figure SD1** Non-metric multidimensional scaling (NMDS) analyses showing differences in cotton-strip microbial community composition characterised by next-generation sequencing (NGS) for relative counts of bacterial OTUs (based on 16S rRNA gene data). Communities were sampled from cotton-strips assayed at locations upstream (U1) and downstream (D) of wastewater inputs at ten study sites sampled in 2013. Two examples of potential indicator taxa are shown (see Table SD5 below for more information). Dashed lines indicate site pairs, and convex hulls overall differences between sampling locations.

**Figure SD2** Stacked bar chart showing bacterial community composition (based on 16S rRNA gene data) by Order from cotton strips in 10 streams in Switzerland with sampling locations below (Downstream) and above (Upstream) of WWTPs.

**
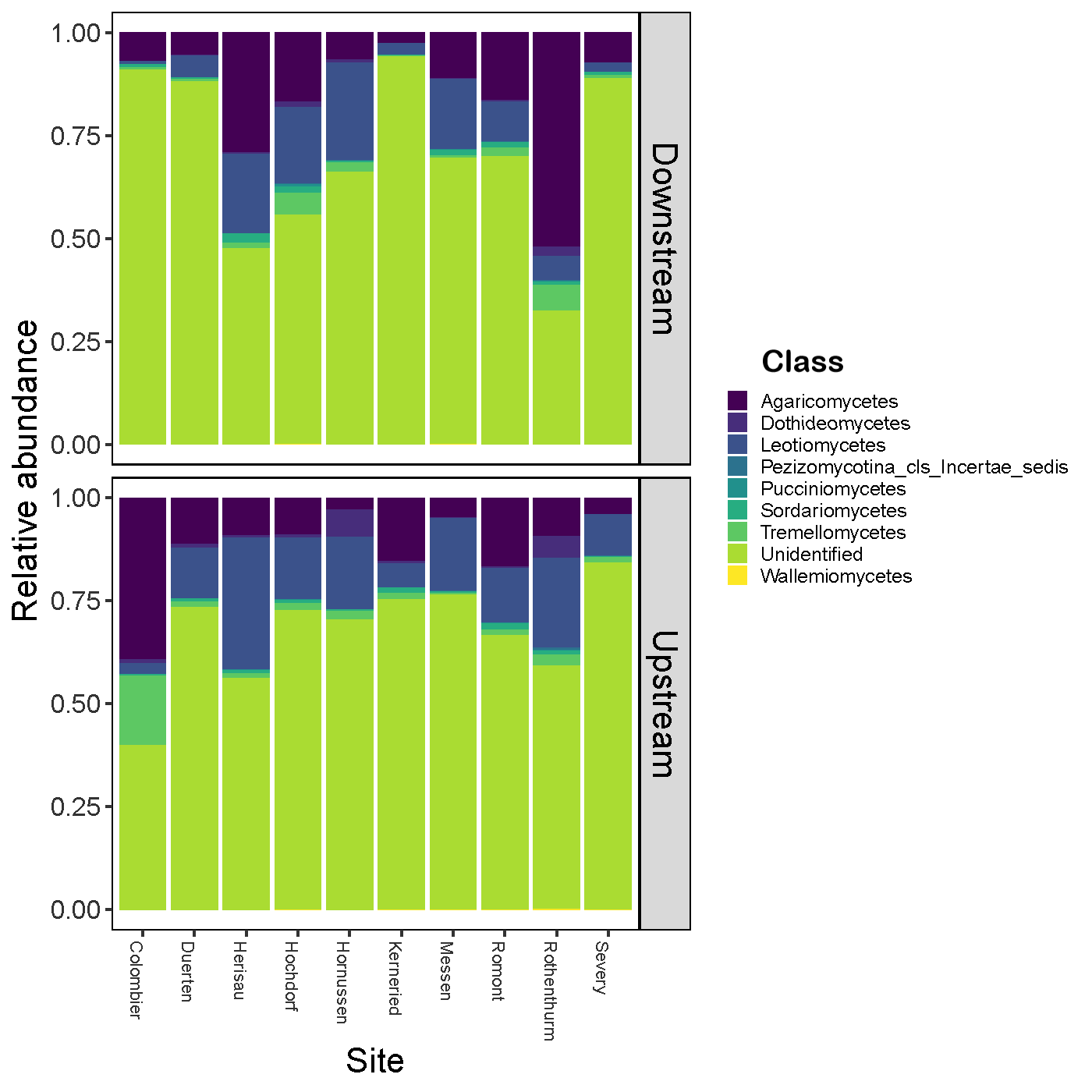
**

**Figure SD3** Stacked bar chart showing fungal community composition (based on ITS1 gene data) by Class from cotton strips in 10 streams in Switzerland with sampling locations below (Downstream) and above (Upstream) of WWTPs.

Table SD5 Results from DESeq2 analysis of bacterial assemblages (based on 16S rRNA gene data) from cotton strips incubated in 10 streams in Switzerland with sampling locations below (Downstream) and above (Upstream) of WWTPs. Mentioned in the Main Text as potential indicator taxa were OTU31 (Flavobacteriaceae: *Flavobacterium*) and OTU130 (Cytophagaceae: *Lacihabitans*). Please note that sampling location D is contrasted with U1 as the reference level. Taxa are ranked by the column ‘log2FoldChange’ which is the effect size estimate (Love et al. 2014). ‘lfcSE’, standard error estimate for the log2FoldChange estimate; ‘stat’ & , Wald statistic and p-value; ‘padj’, Benjamini-Hochberg (BH) adjustment estimates the FDR (false discovery rate).

Table SD6 Results from DESeq2 analysis of fungal assemblages (based on ITS1 gene data) from cotton strips incubated in 10 streams in Switzerland with sampling locations below (Downstream, D) and above (Upstream, U1) of WWTPs. Mentioned in the Main Text as potential indicator taxa were OTU114 (Trichosporonaceae: Unidentified) and OTU335 (Bulleribasidiaceae: *Vishniacozyma*), which were determined looking at occupancy data and *not* these results [because the ITS1 region is highly variable in length and copy number per cell meaning significant bias to some fungal taxa and groups (Lindahl et al. 2013)]. Please note that sampling location U1 is contrasted with D as the reference level, but we remind the reader that these results should be treated with *extreme caution*. Taxa are ranked by the column ‘log2FoldChange’ which is the effect size estimate (Love et al. 2014). ‘lfcSE’, standard error estimate for the log2FoldChange estimate; ‘stat’ & , Wald statistic and p-value; ‘padj’, Benjamini-Hochberg (BH) adjustment estimates the FDR (false discovery rate).

**Table SD7** Significant environmental predictors associated with biodiversity indicators (α-diversity) from NGS (OTUs) of bacterial and fungal communities on cotton strips. Linear mixed models with random intercepts for Site were fitted with restricted maximum likelihood (REML) and reduced using likelihood ratio tests (except Fungal richness; see below). Values for the fixed intercept are only reported where no predictor was significant (i.e., holding the fixed effect constant). IRR, Incident rate ratio (for fungal richness), Diversity, Shannon diversity index (*H*); Evenness, Pielou´s *J'*; Dominance, Berger-Parker index; Rareness, Fisher´s alpha.

| Community | Indicator | Predictor | Estimates / IRR | 95% CI | *P* | Marginal *R^2^* | Conditional *R^2^* |
| --- | --- | --- | --- | --- | --- | --- | --- |
| Bacteria | Richness | Non-fungicides | -89.94 | -144.7 – -35.2 | **0.001** | 0.355 | 0.645 |
| (16S rRNA) |  | Nitrogen | 79.34 | 12.4 – 146.3 | **0.020** |  |  |
|  | Diversity | Intercept | 47.64 | 43.10 – 52.18 | **<0.001** | 0.000 | 0.344 |
|  | Evenness | Location (U1) | 0.033 | 0.002 – 0.064 | **0.035** | 0.389 | 0.784 |
|  |  | Non-fungicides | 0.032 | 0.013 – 0.051 | **0.001** |  |  |
|  |  | Temperature | 0.027 | 0.006 – 0.049 | **0.013** |  |  |
|  | Dominance | Location (U1) | -0.253 | -0.439 –0.061 | **0.011** | 0.288 | 0.441 |
|  |  | Non-fungicides | -0.139 | -0.234 – 0.040 | **0.012** |  |  |
|  | Rareness | Intercept | 4.48 | 4.38 – 4.57 | **<0.001** | 0.000 | 0.300 |
| Fungi (ITS1) | Richness^1^† | Location (U1) | 1.22 | 1.17 – 1.26 | **<0.001** | 0.173 | 0.965 |
|  | Richness^2^ | Fungicides | 0.96 | 0.93 – 0.99 | **0.022** | 0.032 | 0.954 |
|  | Richness^3^ | Location (U1) | 2.19 | 1.79 – 2.68 | **<0.001** | 0.092 | 1.000 |
|  |  | Fungicides | 0.49 | 0.08 – 3.15 | 0.454 |  |  |
|  | Richness^4^ | Location (U1) × Fungicides | 0.92 | 0.88 – 0.96 | **<0.001** | 0.068 | 0.954 |
|  |  | Location (D) × Fungicides | 1.01 | 0.97 – 1.05 | 0.582 |  |  |

† Fungi richness was tested with four generalised linear mixed models (GLMM), each assuming a Poisson distribution for count data. ^2^The slope parameter estimate for the relationship between fungal richness and fungicide TUs in this GLMM is -0.037 (the Pearson correlation coefficient is -0.37), demonstrating the general negative influence of this MP class on fungal richness (Fig.SD5). ^3^Here fungal richness was tested with a random slopes GLMM using Location (U1,D) and Fungicide TUs as the fixed effects. There was markedly more support for this model than the random intercepts model (ΔBIC = 71.4), hence this model is presented here. ^4^In EDA and model iterations there appeared to be an ecologically-relevant interaction between Location and Fungicides TUs. This pattern (Fig.SD4) was confirmed by the fourth fungal richness GLMM.

**Table SD8** Significant environmental predictors associated with biodiversity indicators (β-diversity) from next-generation sequencing (OTUs) of bacterial and fungal communities on cotton strips. Linear mixed models were fitted with restricted maximum likelihood (REML) and reduced using likelihood ratio tests. Values for the intercept are only reported where no predictor was significant (holding the fixed effect constant). NMDS, non-metric multidimensional scaling (site scores for axes 1−2); RA, relative abundance (relative counts); PA, presence-absence (occupancy).

| Community | Indicator | Predictor | Estimates | 95% CI | *P* | Marginal *R^2^* | Conditional *R^2^* |
| --- | --- | --- | --- | --- | --- | --- | --- |
| Bacteria (16S rRNA) | NMDS1 RA | Intercept | 0.000 | -0.125 – 0.125 | 1.000 | 0.000 | 0.846 |
|  | NMDS2 RA | Temperature | -0.155 | -0.213 – -0.097 | **<0.001** | 0.665 | 0.817 |
|  |  | Non-fungicides | -0.059 | -0.101 – -0.017 | **0.006** |  |  |
|  | NMDS1 PA | Nitrogen | 0.044 | 0.009 – 0.092 | **0.012** | 0.159 | 0.383 |
|  | NMDS2 PA | Location (U1) | 0.043 | 0.002 – 0.083 | **0.038** | 0.678 | 0.754 |
|  |  | Temperature | -0.062 | -0.085 – -0.038 | **<0.001** |  |  |
|  |  | Nitrogen | 0.034 | 0.009 – 0.059 | **0.007** |  |  |
| Fungi (ITS1) | NMDS1 PA | Arable cropping | 0.088 | 0.024 – 0.152 | **0.032** | 0.244 | 0.498 |
|  | NMDS2 PA | Nitrogen | -0.099 | -0.173 – 0.024 | **0.023** | 0.235 | 0.433 |


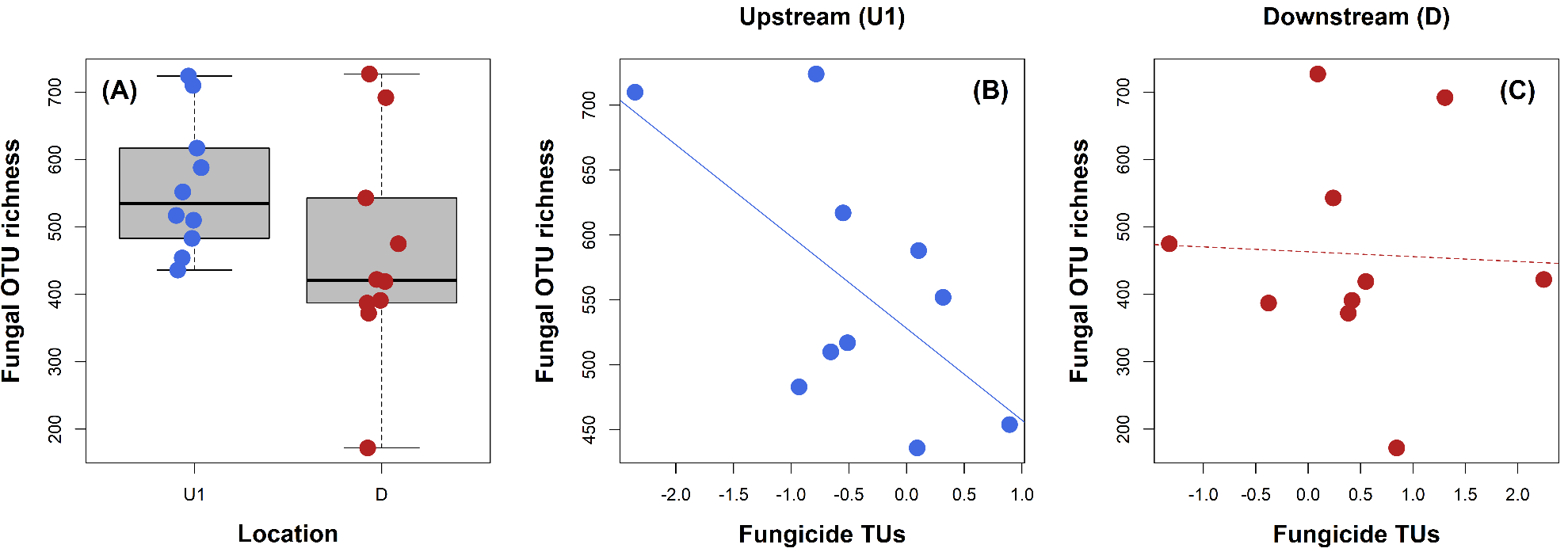


**Figure SD4** Fungal OTU richness on cotton strips (A) declined (*P* < 0.001) at sampling locations downstream (D, red) of WWTPs when compared with reference (unimpacted) locations upstream (U1, blue). (B) We found evidence for a negative relationship between fungicides TUs and fungal OTU richness at upstream sites (*P* < 0.001), but the relationship between fungicides TUs and fungal OTU richness at downstream sites was more equivocal and statistically non-significant (Table SD7), although the range of values were considerably higher than at upstream sites, meaning a threshold may have been crossed for sensitive species. Note that fungicides TUs are log-transformed and standardised.


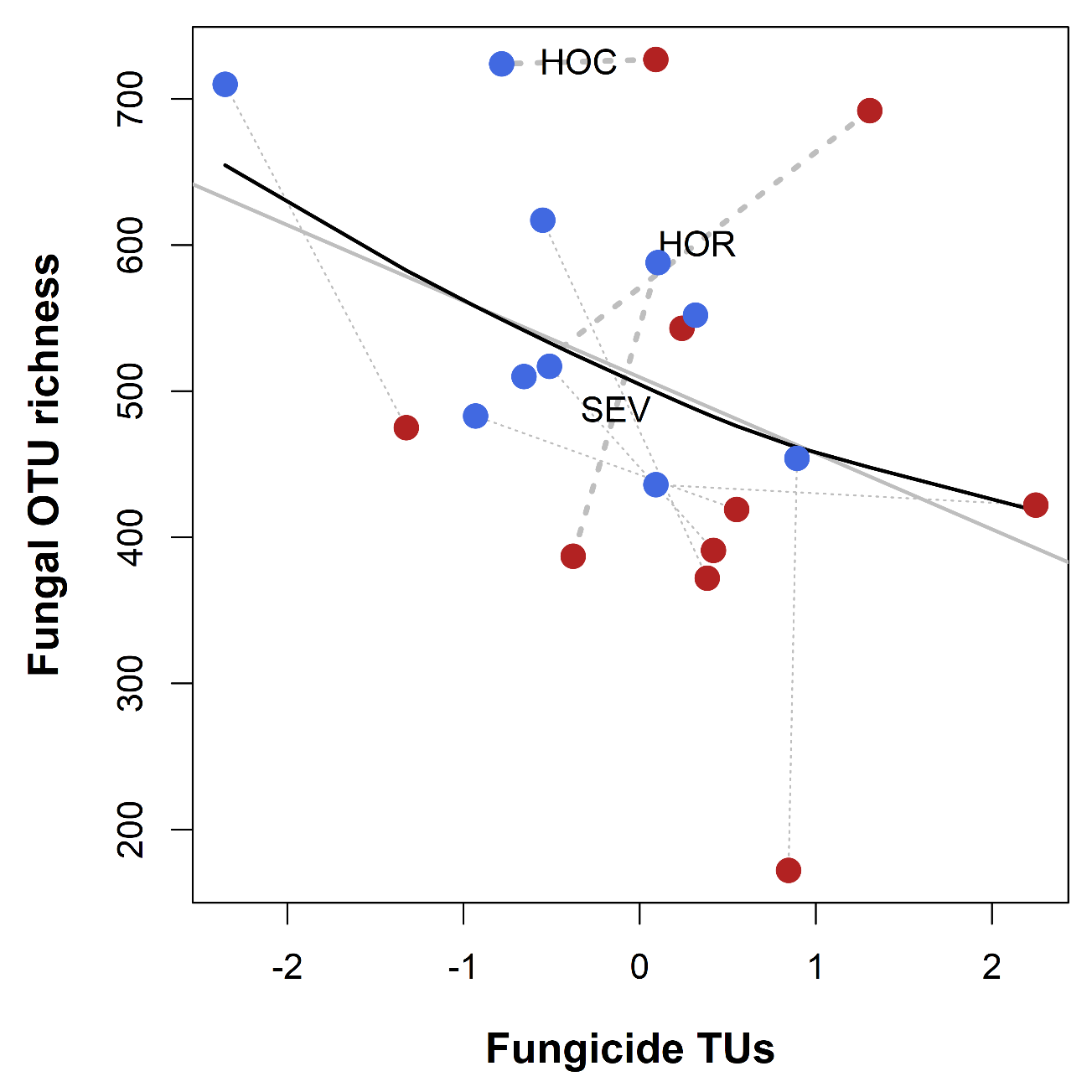


**Figure SD5** Fungal OTU richness showed an overall negative relationship (*P* < 0.05) with increasing toxic units (TUs) of fungicides at study locations upstream (blue) and downstream (red) of WWTPs (Table SD7). However, there was considerable context-dependency in responses at the site level, and three sites in particular (HOC, HOR, and SEV) had atypical responses between sampling locations, making the location effect difficult to discern from the increasing toxicity of MPs. Fitting a GLMM with random slopes (and sampling location as a control variable) accounted for these atypical influences and resulted in a negative parameter estimate for fungicide TUs (-0.708), albeit non-signficant at *α* = 0.05 (Table SD7) because of a stronger negative influence of sampling location D below the WWTPs. We also saw evidence for the relationship to be non-linear (smoothing funtion in black), with loss in fungal OTU richness slowing with increasing fungicide toxicity. We hypothesise that this may be because as toxicity increases sensitive species are lost, leaving behind only tolerant species (other hypotheses include ecological/evolutionary ‘rescue’ from the WWTP microbiome, e.g., HOR above). Some of the noise in the relationship can be attributed to measurement errors in fungal richness and fungicides TUs, and the fact that non-fungicide TUs from WW inputs could potentially add cumulative toxicity effects at some sites (e.g., SEV). Note that fungicides TUs are log-transformed and standardised.

**Table SD9** Results from redundancy analysis and variation partitioning models of microbial communities (see also D.1.2 above). Full methods description are given at C.1.4. Percentage of explained variation (*R^2^_ad_*_j_) are shown for each predictor; ^*^ indicates where the independent contribution is significant (*P* < 0.05).

|  | Model selection | | |
| --- | --- | --- | --- |
|  | Blanchard et al. (2008)  (spatial and environmental) | Combined approach  (spatial and environmental) | Positive variation  (environmental) |
| Bacteria PA  (16S rRNA) | 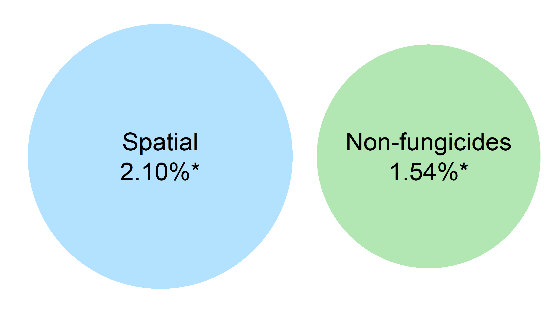 | 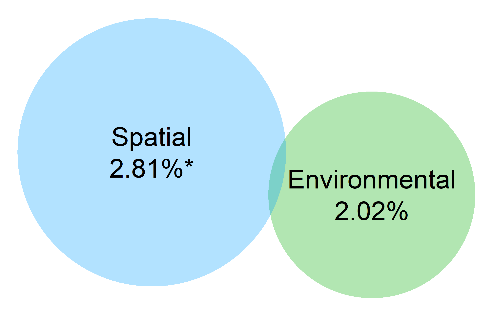 | 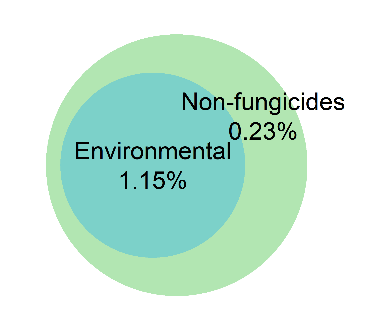 |
| Bacteria RA  (16S rRNA) | 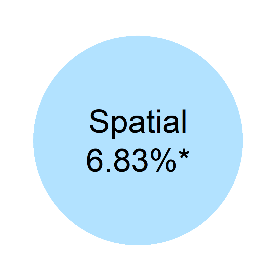 | 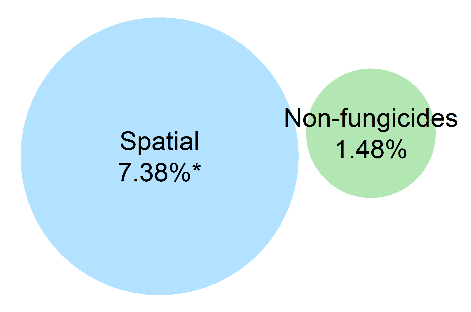 | 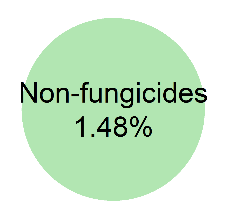 |
| Fungi PA (ITS) | 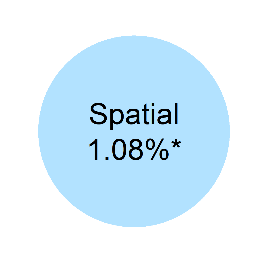 | 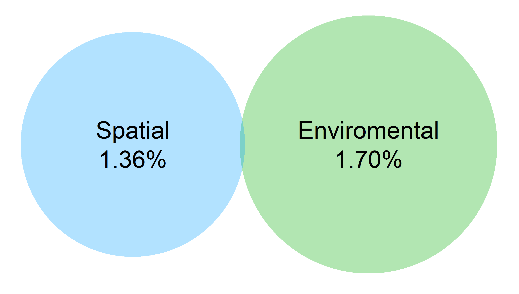 | 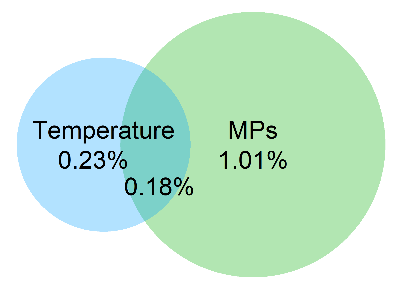 |

**
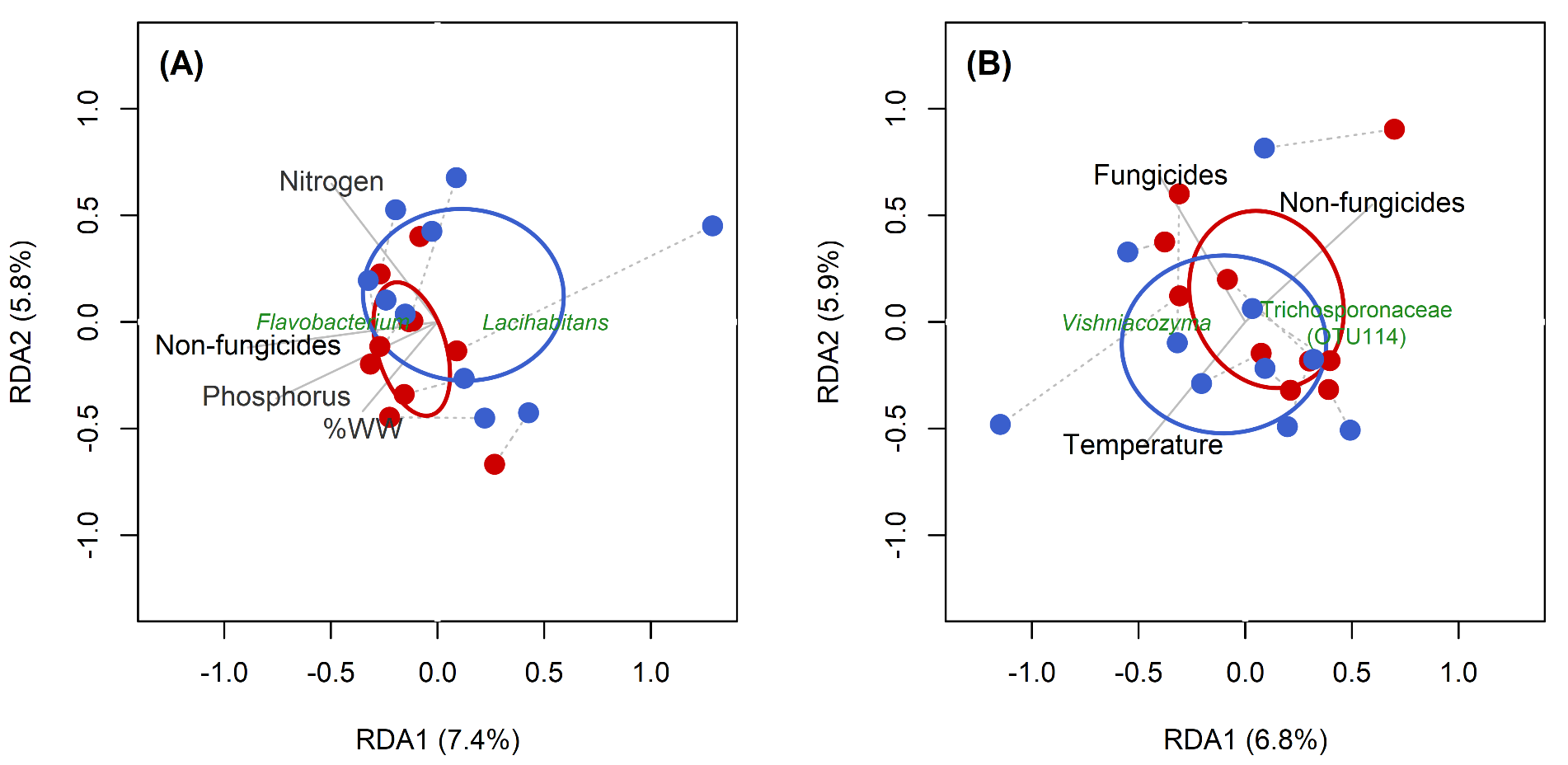
**

**Figure SD6** Partial redundancy analysis testing the influence of environmental predictors on A) bacterial (16S rRNA) and B) fungal (ITS1) community composition using occupancy (presence-absence) data with our ‘combined’ model selection procedure (Table SD9). The model for A) bacterial communities includes four environmental predictors (%WW, concentrations of dissolved inorganic nitrogen and soluble reactive phosphorus, and TUs of non-fungicide MPs), but only non-fungicides MPs had a significant influence on community composition. In contrast, three environmental predictors (TUs of fungicide and non-fungicide MPs, and mean water temperatures) jointly had a significant influence on B) fungal community composition, but we were unable to detect significant independent influences of these predictors. Both models ‘partial’ (condition) out the influence of spatial location (PCNM5). Downstream sampling locations are shown in red, upstream locations in blue. Potential indicator taxa are shown in green (rescaled for clarity). Standard dispersion ellipses represent 95% confidence intervals.

**Figure SD7** Redundancy analysis testing the correlation of temperature-corrected ecosystem functioning (mass loss, tensile-strength loss, and respiration) measured with the cotton strip assay and bacterial community composition (based on 16S rRNA data). Indicated are bacterial taxa strongly responding to the constrained ordination.

**
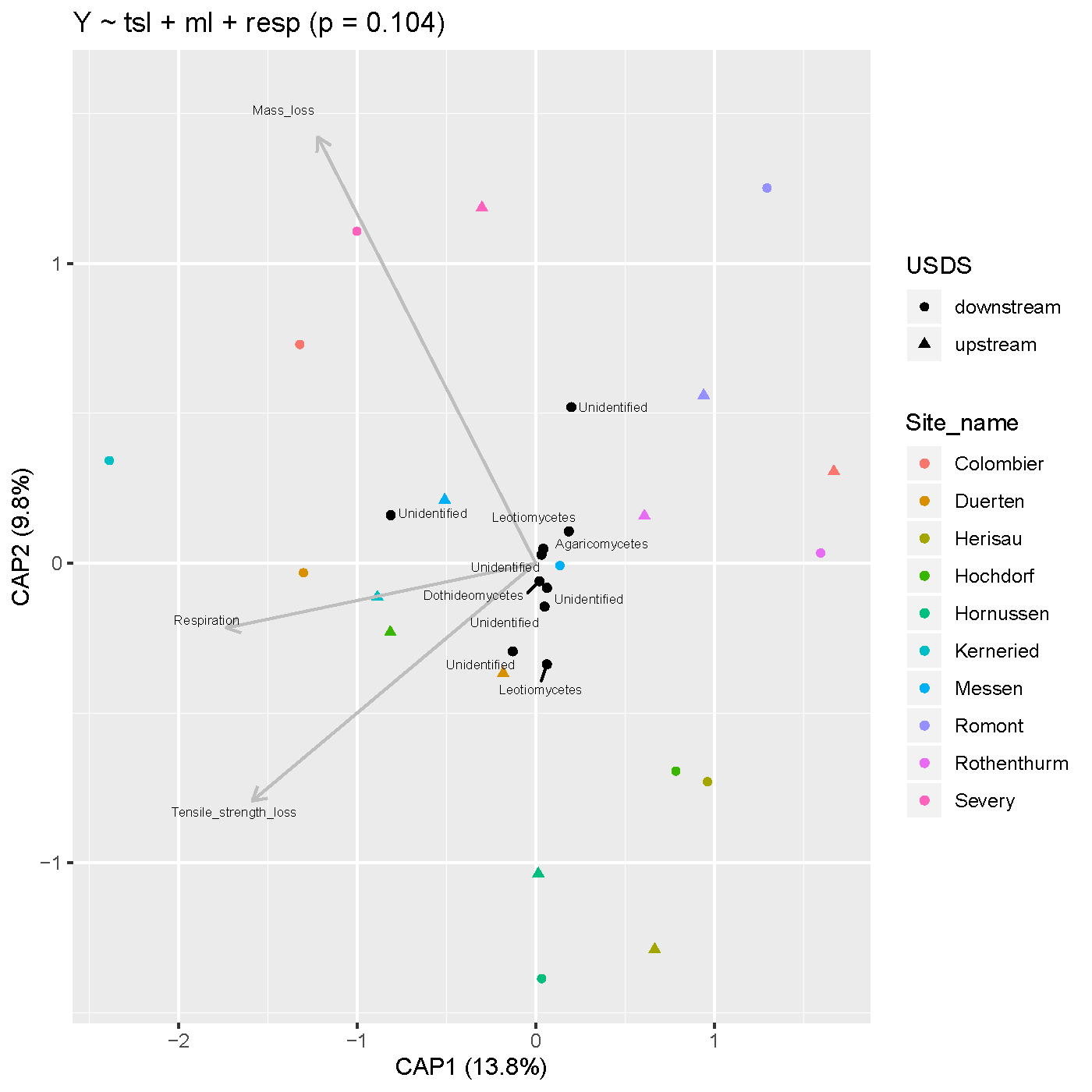
**

**Figure SD8** Redundancy analysis testing the correlation of temperature-corrected ecosystem functioning (mass loss, tensile-strength loss, and respiration) measured with the cotton strip assay and fungal community composition (based on ITS1 gene data). Indicated are fungal taxa strongly responding to the constrained ordination.

### D.1.3 Cotton-strip ARISA

Despite the changes observed in bacterial communities using NGS, the ARISA bacterial community data (Fig.SD9A) did not show significant differences in turnover (*t* = -1.79, *P* = 0.076), nestedness (t = 0.001, *P* = 0.994), or overall dissimilarity (*t* = -1.93, *P* = 0.056). The ARISA fungi community data (Fig.SD9B) showed significant increases in turnover (*t* = -2.75, *P* < 0.01) and overall dissimilarity (*t* = -3.84, *P* < 0.001) at downstream locations, but not in nestedness (*t* = 0.388, *P* = 0.698).


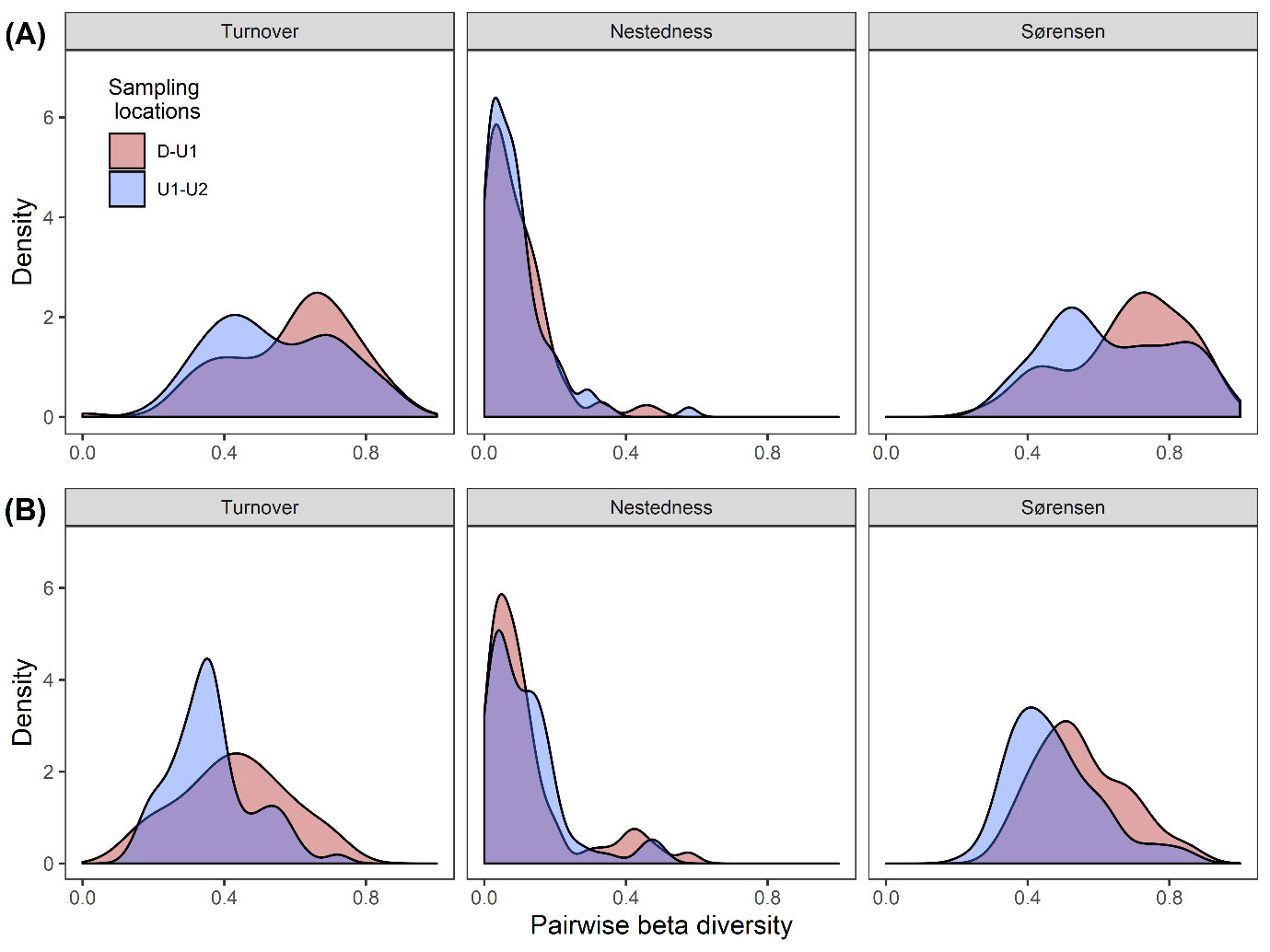


**Figure SD9** Density plots shows changes in beta-diversity of cotton-strip microbial communities characterised using ARISA DNA-fingerprinting for (A) bacteria and (B) fungi. Communities were described from cotton-strips assayed at locations upstream (U1, U2) and downstream (D) of wastewater inputs at ten study sites sampled in 2013.

### D.1.4 Ecosystem functioning based on the cotton-strip assay


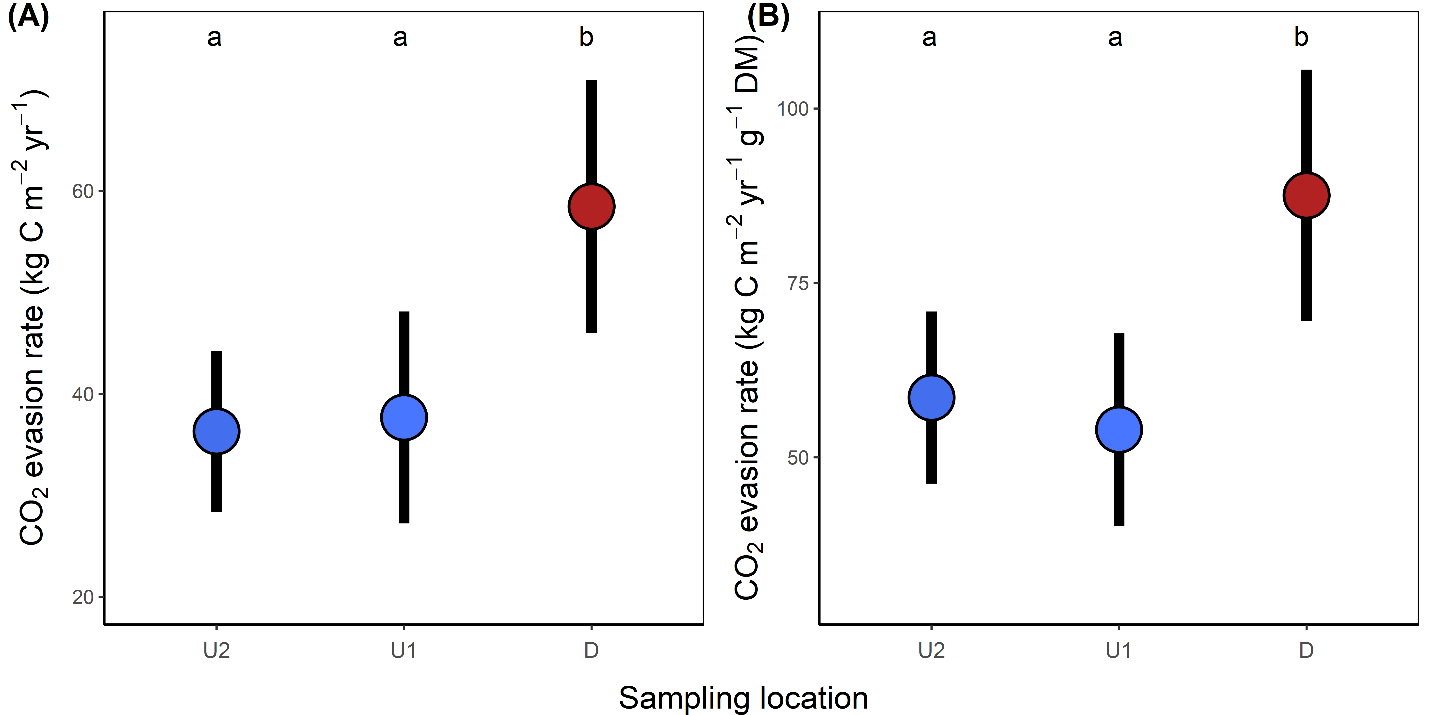


**Figure SD10** Mean estimated efflux of carbon dioxide (± 95% CI) as measured by the cotton-strip assay (CSA) at 12 Swiss streams. Sampling locations were above (U2, U1) and below (D) WWTPs. Panel A show mean annual rates of carbon efflux (kg C m^-2^ yr^-1^ g DM^-1^), whereas Panel B shows mean rates corrected for remaining mass of the cotton strip (kg C m^-2^ yr^-1^ g DM^-1^). Temperature effects on evasion rates have been accounted for in both estimates.

**
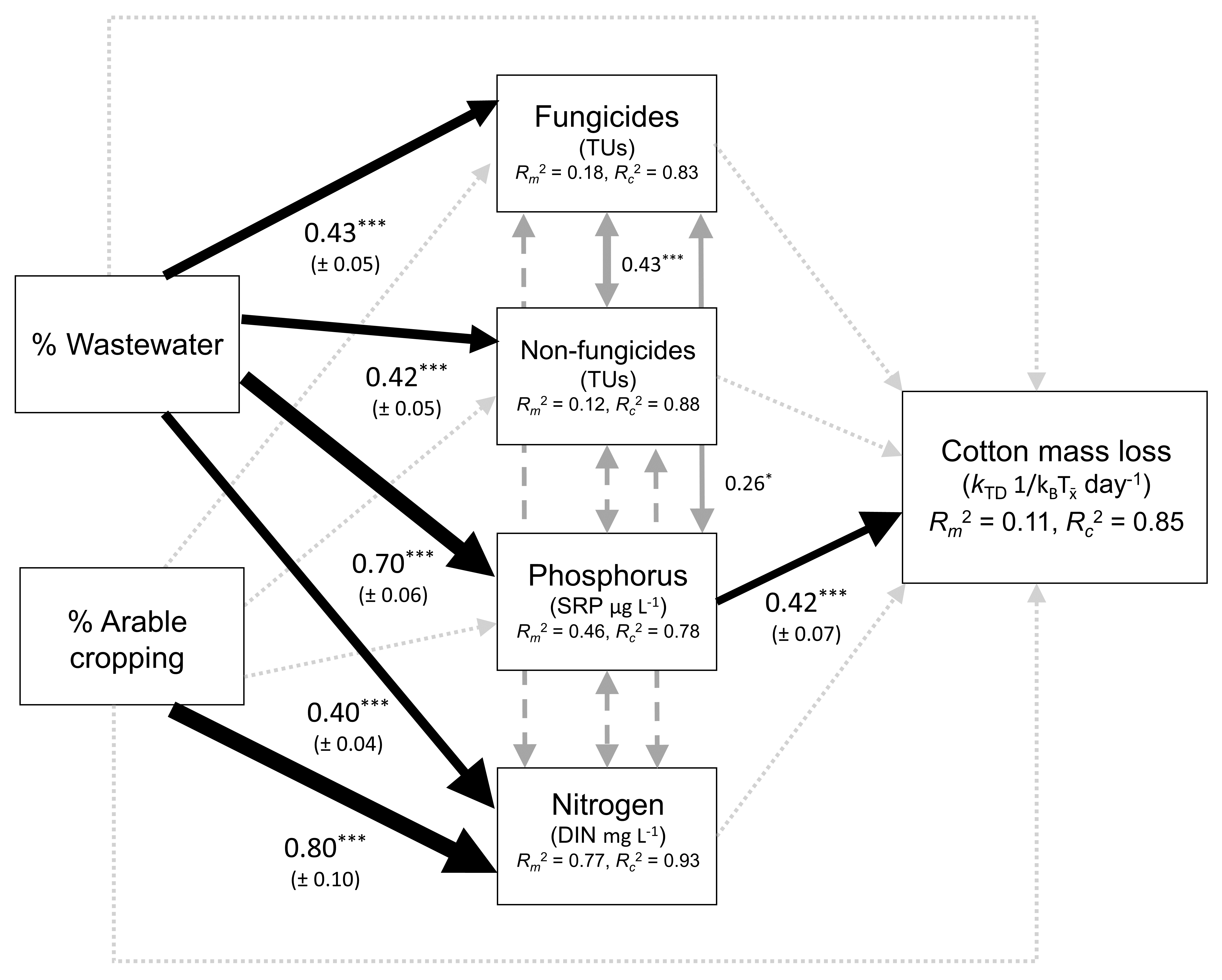
**

**Figure SD11** Piecewise structural equation model showing the influence of arable cropping (% upstream catchment), inputs of wastewater (% discharge), and associated stressors (MPs, nutrients) on temperature-corrected rates of mass loss as measured by the cotton-strip assay (CSA) at 20 streams in Switzerland with sampling locations above (U2,U1) and below (D) WWTPs. A random effect term accounts for the non-independence of “Site” nested in “Year”. Solid black lines indicate significant positive influences; scaled to the strength of the relationship. Solid gray lines indicate significant correlated errors, dashed grey lines non-significant correlated errors. Dotted gray lines indicate non-significant paths tested and discarded during model selection. The global model included a term for sampling location (U2 vs U1, D) for each endogenous variable, but these were discarded during model selection. Standardised values for path coefficients (± 1 S.E.) are indicated. Marginal *R^2^* values indicate the goodness of fit for endogenous variables excluding variance explained by the random effects. Conditional *R^2^* values indicate both fixed and random variance. Fisher´s *C* = 14.3, *P* = 0.574, 16 *d.f.*, ΔBIC = 59.4.

^*^ *P* < 0.05; ^***^ *P* < 0.001

#### Biodiversity and cotton tensile loss

Although there were no biodiversity-mediated influences on tensile-strength loss in Fig.3b, MT, an alternative model with less support (ΔBIC = -0.869) suggested a strong association between bacterial community composition (β-diversity) and tensile-strength loss indirectly affected by WW via changes in phosphorus concentrations (Fig.SD12).


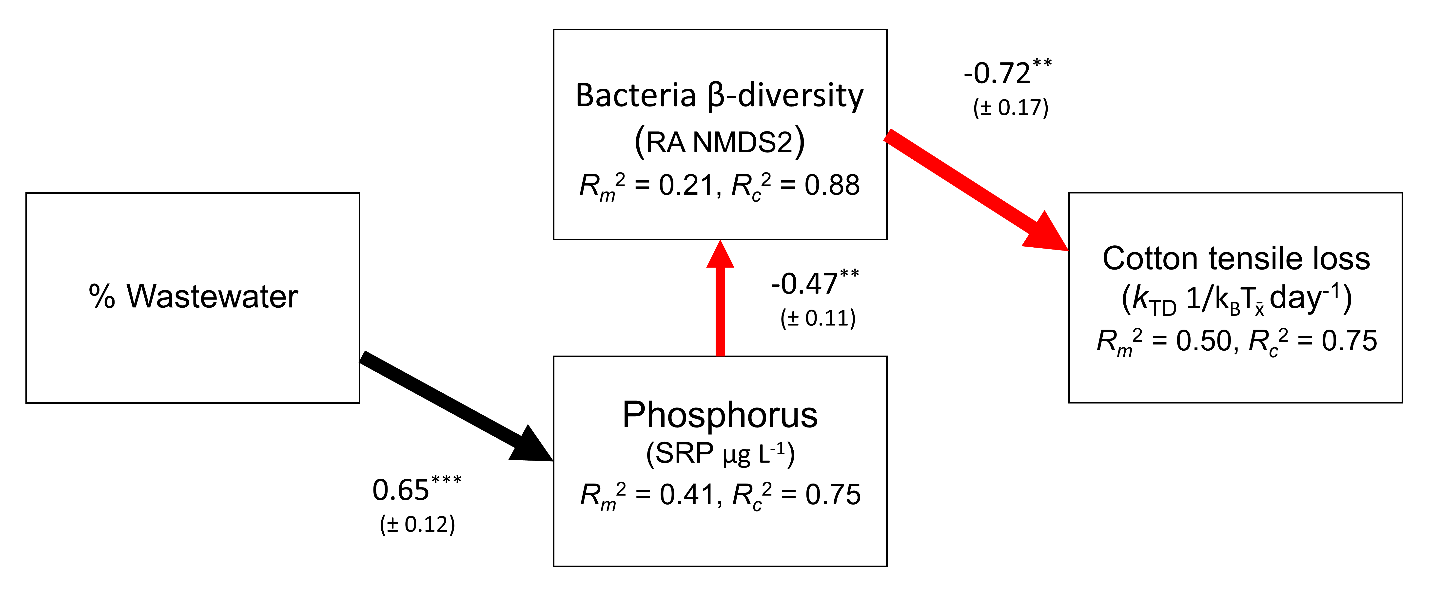


**Figure SD12** Alternate piecewise structural equation model showing the influence of inputs of wastewater on a temperature-corrected indicator of ecosystem functioning (tensile-strength loss) as measured by the cotton-strip assay (CSA). Mean values are used from ten 2013 study sites with sampling located above (U1) and below (D) wastewater inputs. A random effect term accounts for the non-independence of site. Solid black lines indicate significant positive influences; red significant negative influences; both are scaled to the strength of the relationship. Standardised values for path coefficients (± 1 S.E.) are indicated. Marginal *R^2^* values indicate the goodness of fit for endogenous variables excluding variance explained by the random effects. Conditional *R^2^* values indicate both fixed and random variance. Fisher´s *C* = 6.832, *P* = 0.337, 6 *d.f.*, ΔBIC = -0.869 (i.e., a larger BIC score than the best fitting model Fig.3b, MT).

^**^ *P* < 0.01; ^***^ *P* < 0.001

## D.2 Experimental results

### D.2.1 Experiment 1

#### Water quality

Mixing conditions:

The nominal fractions of WW mixed into the river water were 0% (control), 20% WW (low), 50% WW (medium), and 90% WW (high). To determine the real fractions of WW (*f_WW_*), grab water samples were taken weekly in the buffer tanks containing the river water (*C_river_*) and the WWTP effluent (*C_WW_*) and in the distributing units (*C_treat_*) from where the water specific for a treatment was distributed to the flumes. These concentration measurements were used to calculate the *f_WW_* as follows:

| $f_{WW}=\frac{C_{treat}-C_{river}}{C_{WW}-C_{river}}$ | (Eq.SD1) |
| --- | --- |

The *f_WW_*-values were calculated based on measured electrical conductivity, Na^+^, and Cl^-^ concentrations (Table SC1). For each sampling time, these data were very consistent. The average across all sampling dates was taken as the empirical value for each treatment.

*Nutrient levels:* The same grab samples from the distribution units as mentioned above were used to determine the nutrient levels in the different treatments (Table SC10).

**Table SD10**: Observed fractions of wastewater (WW) in the different treatments calculated from measured water chemistry parameters during Exp.1.


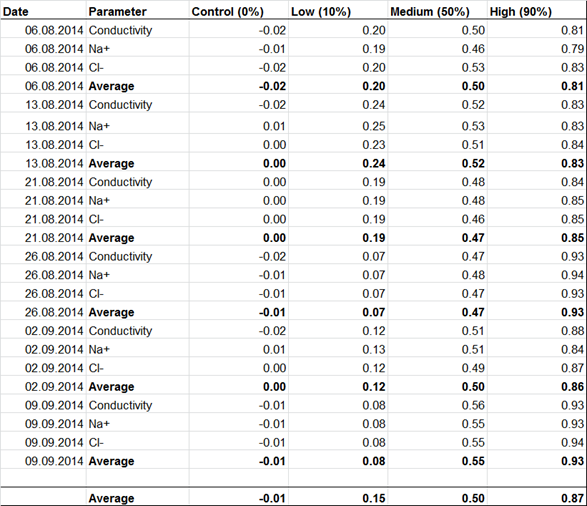

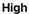

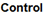

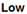

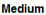


**Table SD11**. Observed nutrient levels in the different treatments during Exp.1.


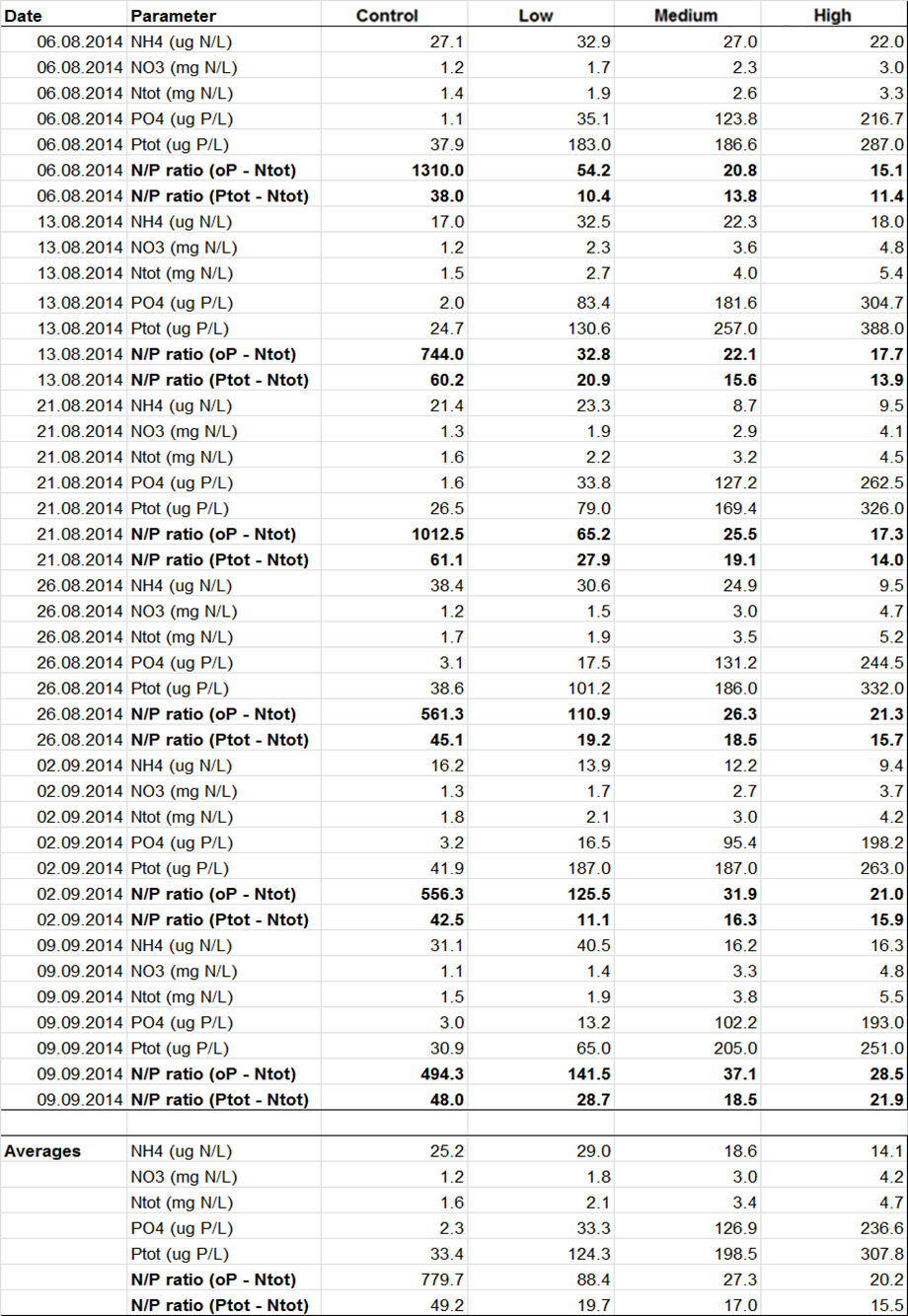


Comparison between expected and observed concentrations:

Based on the nutrient concentration in the river water and the effluent from the WWTP on the one hand and the fraction of WW determined from a conservative tracer (such as Cl or electrical conductivity) on the other hand, it is possible to calculate the expected nutrient concentrations for the four treatment. Comparing these expected values to measured values revealed some systematic patterns (see Fig.SD13). For ammonia and phosphate, the expected concentrations were mostly larger than the measured values, while for nitrate the opposite was the case (except for the 80% WW treatment). These data suggest that nutrients were taken up (e.g. phosphate) and/or transformed (probably the case for N-forms) in the flumes despite the short residence times of a few minutes.


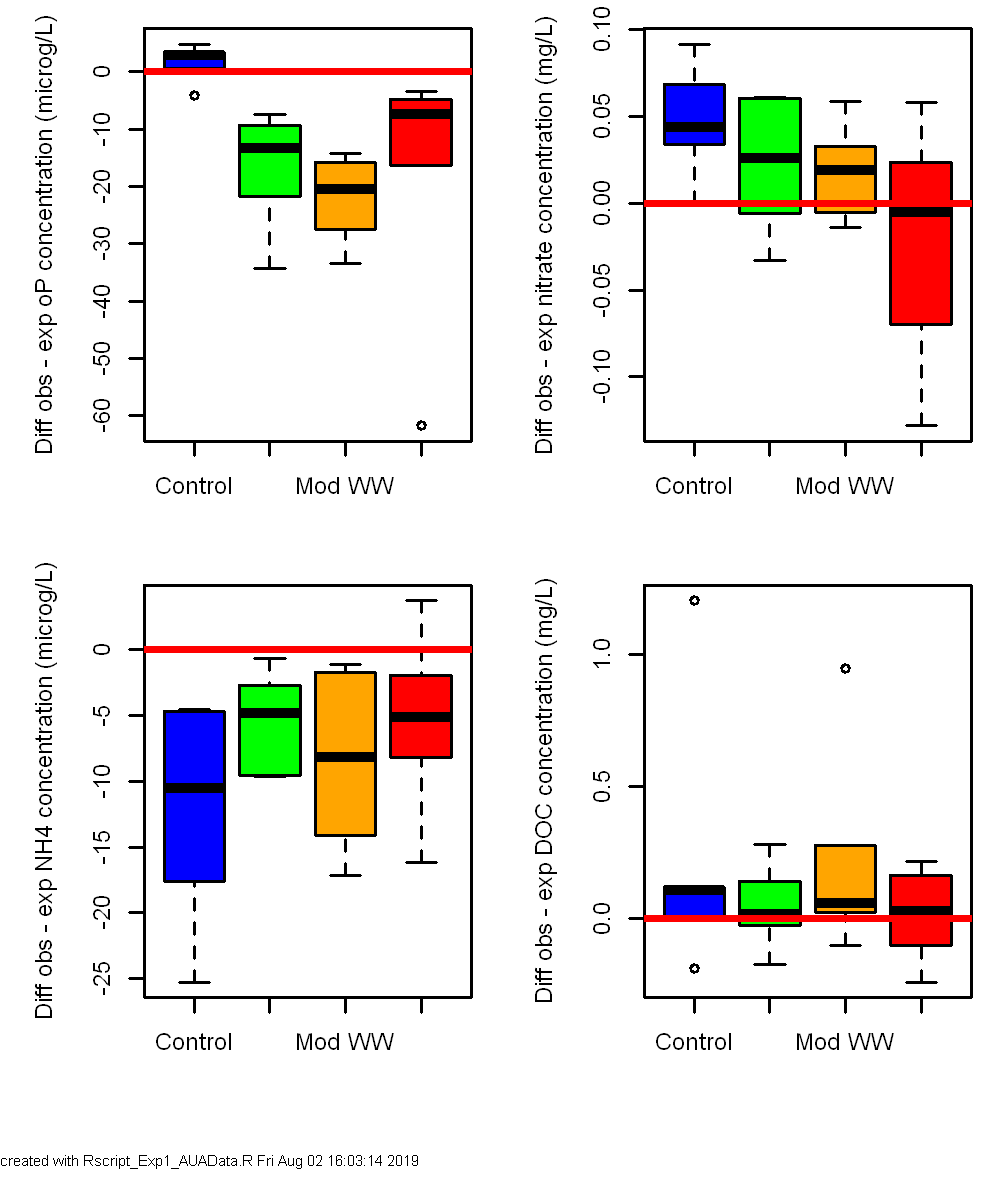


**Figure SD13** Differences between observed and expected nutrient concentrations (SRP, NH4^+^, NO3^-^, DOC) for the four treatment (blue, river Glatt control; green, low WW-level; orange, moderate WW-level; red, high WW-level) during Exp.1.

#### Micropollutants


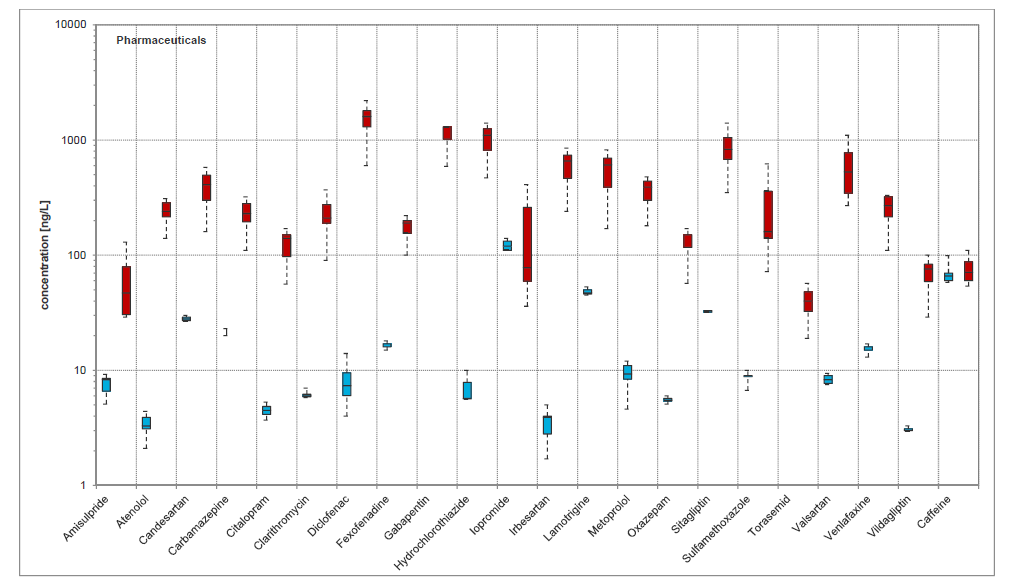


**Figure SD14** Concentration levels of pharmaceuticals in the two source water for the dilution experiment (Exp.1). Red, treated WW; blue, river Glatt.


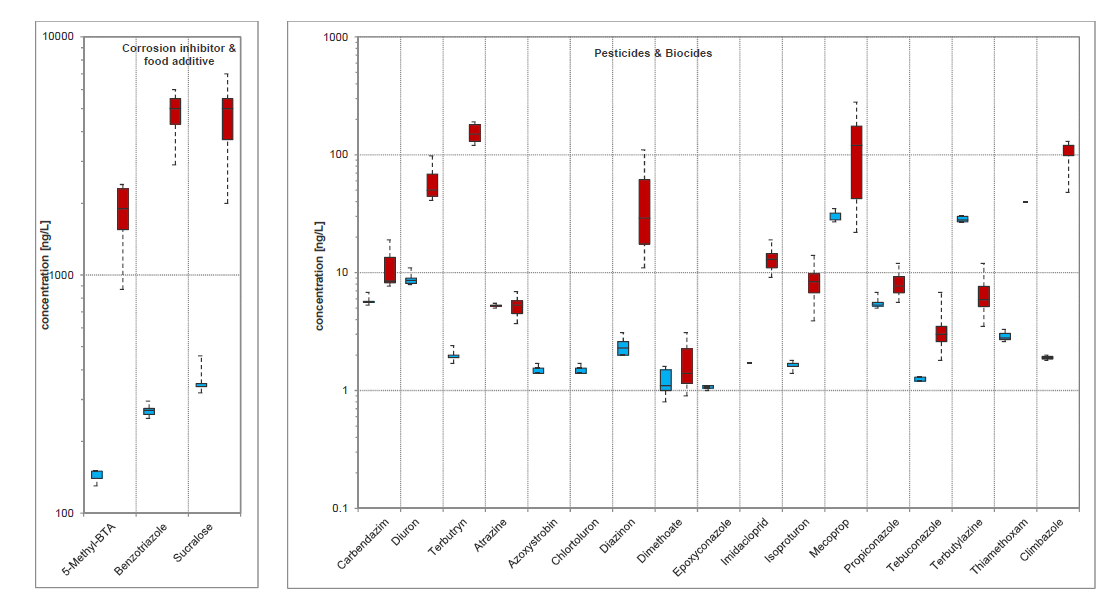


**Figure SD15** Concentration levels of pesticides and corrosion inhibitors in the two source water for the dilution experiment (Exp.1). Red, treated WW; blue, river Glatt.

### D.2.2 Experiment 2

#### Cotton strips

Our field results (e.g., Fig.3, MT) indicated a positive influence of WW-borne nutrients on cotton decomposition. To test whether nutrients ‘mask’ the effects of MPs, we conducted two flumes experiments (Exp.2 & Exp.3). Exp.2 showed that our mixture of MPs had a significantly negative effect on cotton breakdown measured by microbial respiration (*F_3,54_* = 88.5, *P* < 0.001; Fig.D14A), tensile-strength loss (*Χ^2^_3_* = 129, *P* < 0.001; Fig.D14B), and mass loss (*Χ^2^_3_* = 104, *P* < 0.001). There were no significant differences in respiration between the nutrients only and MPs & nutrients treatments. Tensile-strength and mass loss responses indicated that MPs could reduce the positive influence of nutrients when in combination, but not as much as the MPs only treatment. There was a consistent negative effect of the carrier used to keep the MPs and nutrients in solution, because all treatments showed depressed responses relative to the river water control.


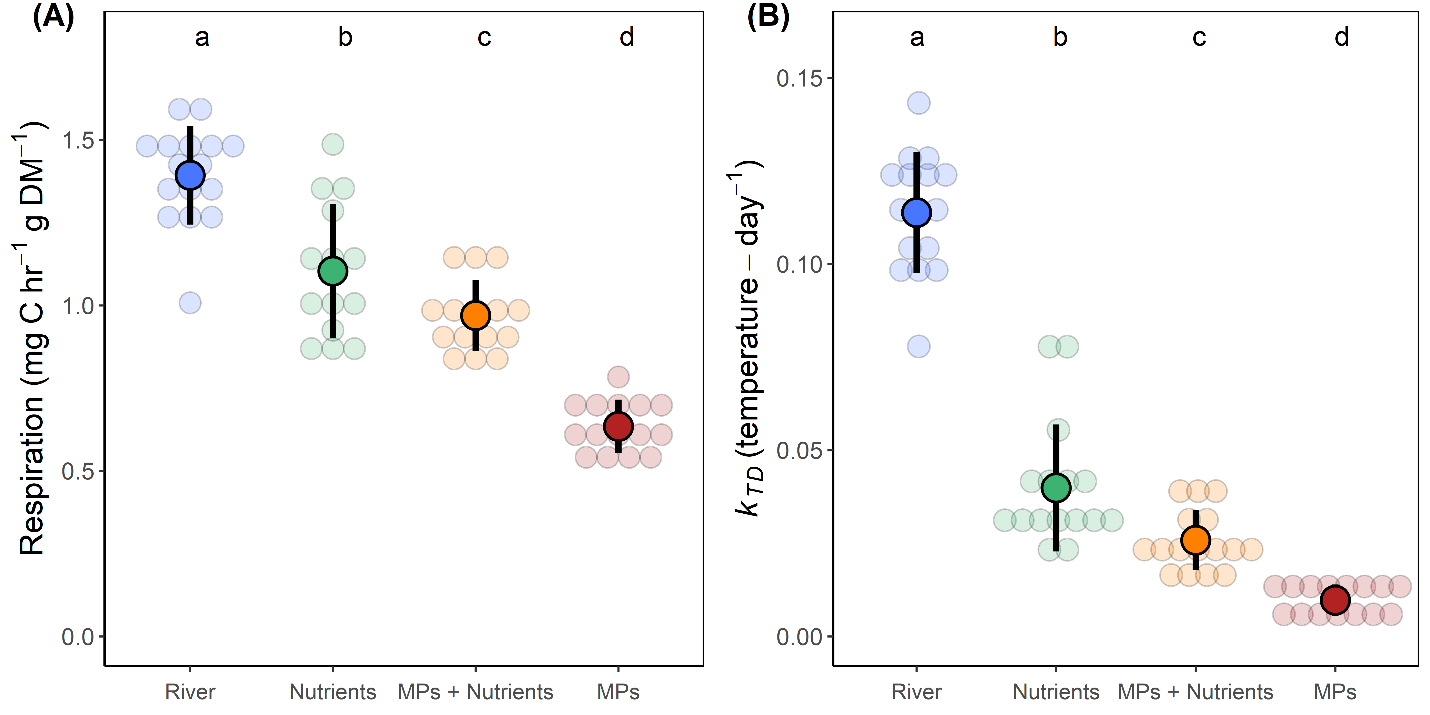


**Figure SD16** Mean (± 1 S.D) rates of cotton-strip A) respiration and B) tensile-strength loss from the first ‘Dosing’ experiment (Exp.2) in the Maiandros flumes system.

#### Water quality

Comparing the nutrient concentrations in the different treatments revealed patterns that suggest that nutrients were quickly metabolised during the passage through the flumes. These patterns were dependent on the treatment. For example, SRP concentrations were consistently lower than the river control indicating that the dosing of MPs induced additional P-consumption in the flumes (Fig.SD17).


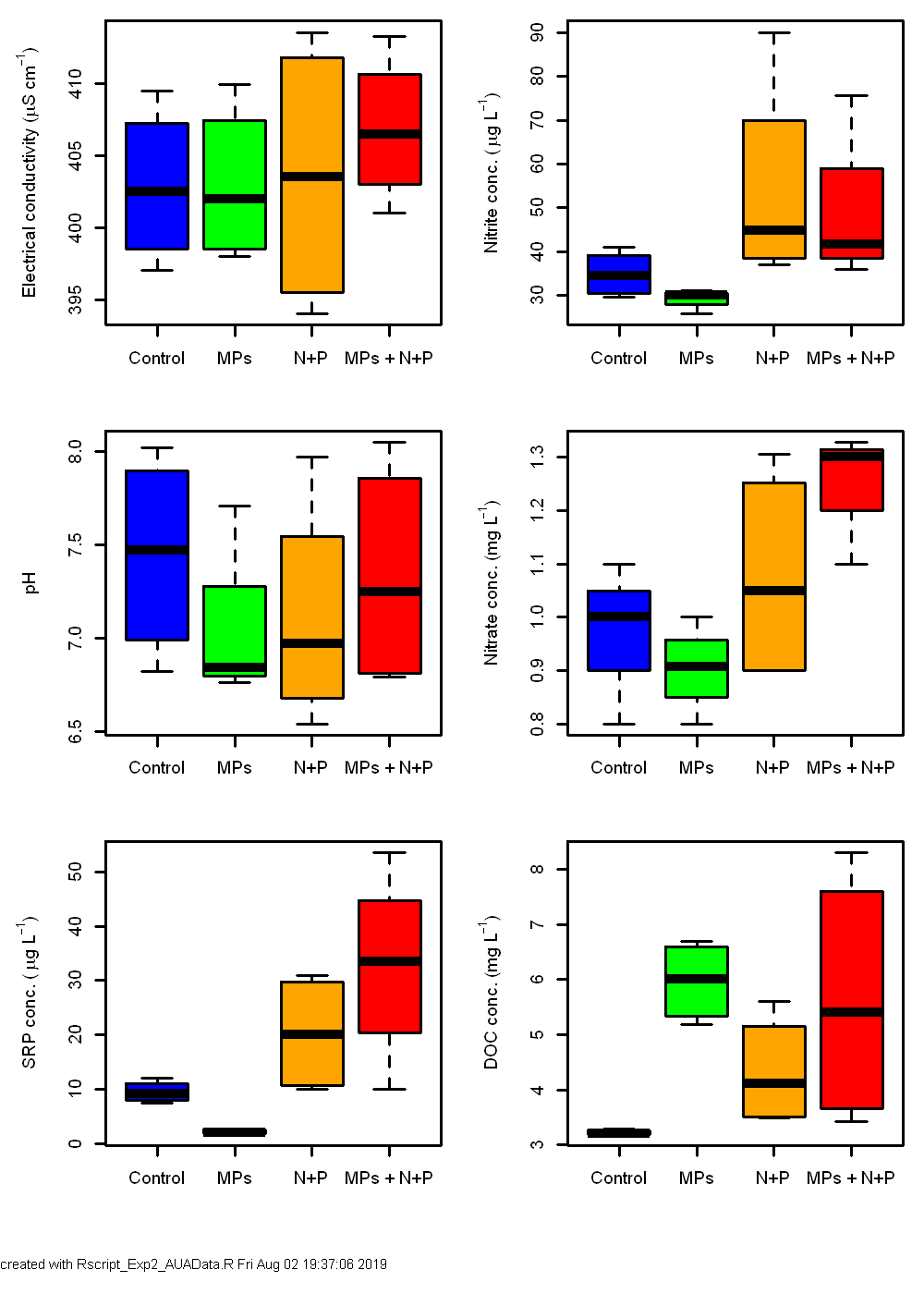


**Figure SD17** Water quality as a function of the four treatments across the 16 channels.

#### Micropollutants

The relative MP concentrations differed between the MP and the MP+N&P treatments (Fig.SD18) despite very similar concentrations in the stock solutions (Fig.SC3, Appendix C). This was most likely due to different pump rates, because the MP treatment achieved only about 90% of the target rate. The rest of the deviations may be due to undetected leaks or by slightly higher inflows of water in the MP treatment.


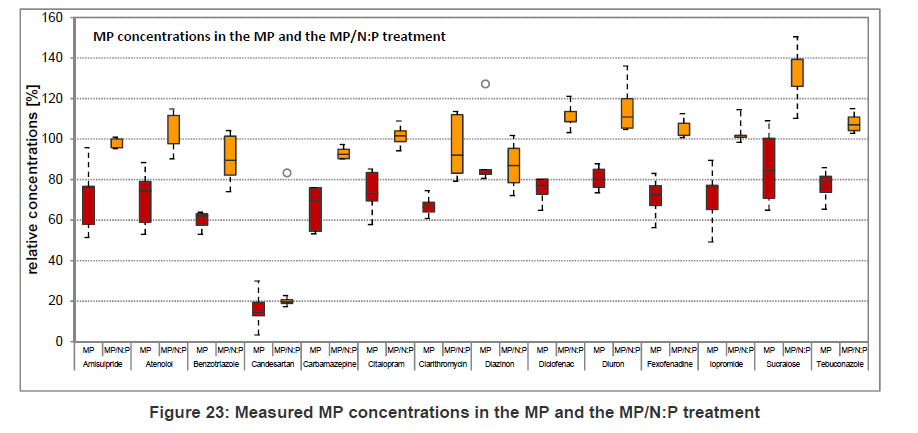


**Figure SD18** Comparison of concentrations (relative to the target concentrations) of 13 out of 17 organic MPs across the 16 channels for the MP and the MP+Nutrient treatment (Exp.2). Diazinone, β-estradiol, and metformin were excluded in the comparison for analytical reasons.

### D.2.3 Experiment 3

#### Water quality

The water quality in the different treatments differed according to the expectations and the dosing. Nutrients levels did not differ between the treatments except for the nutrient dosing treatment (MP+N&P; Fig.SD19-21).


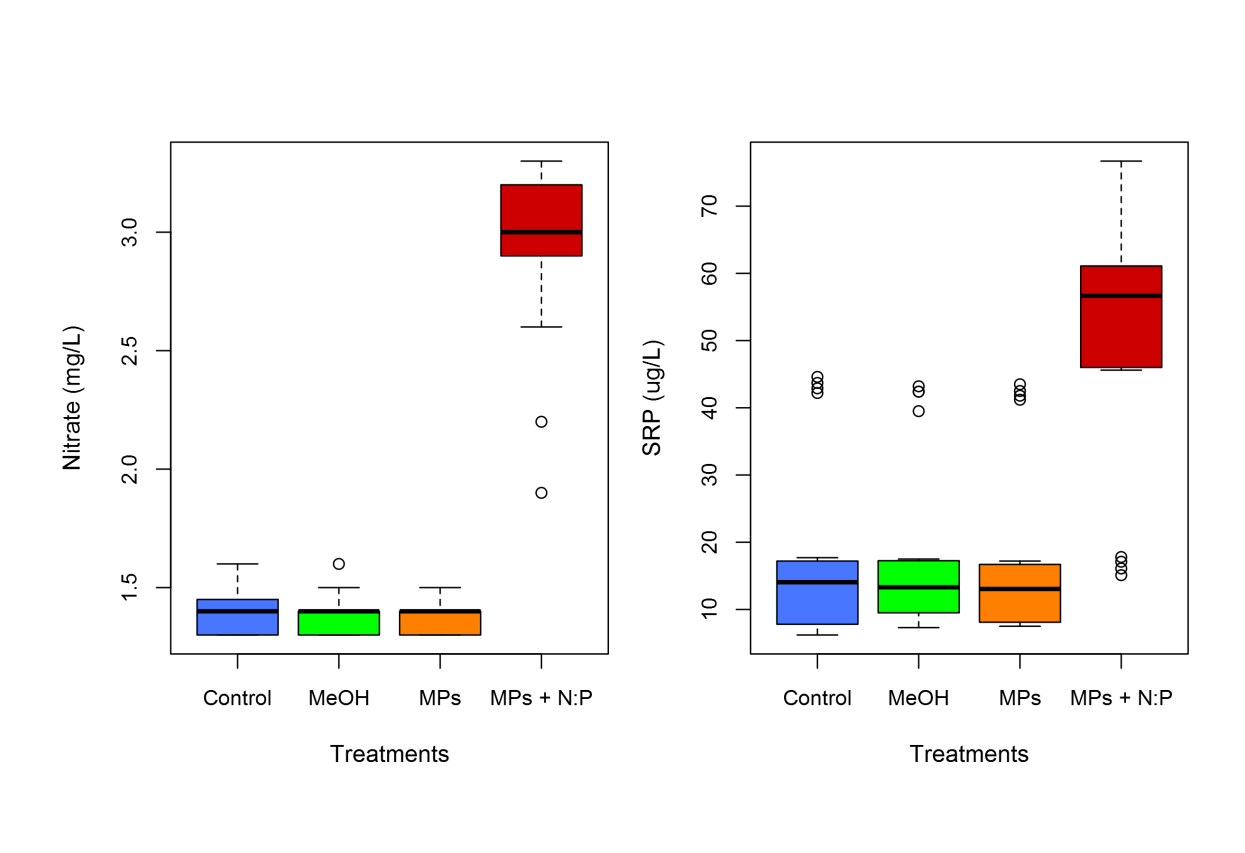


**Figure SD19** Comparison of nitrate and soluble-reactive P levels across the four treatments in Exp.3.


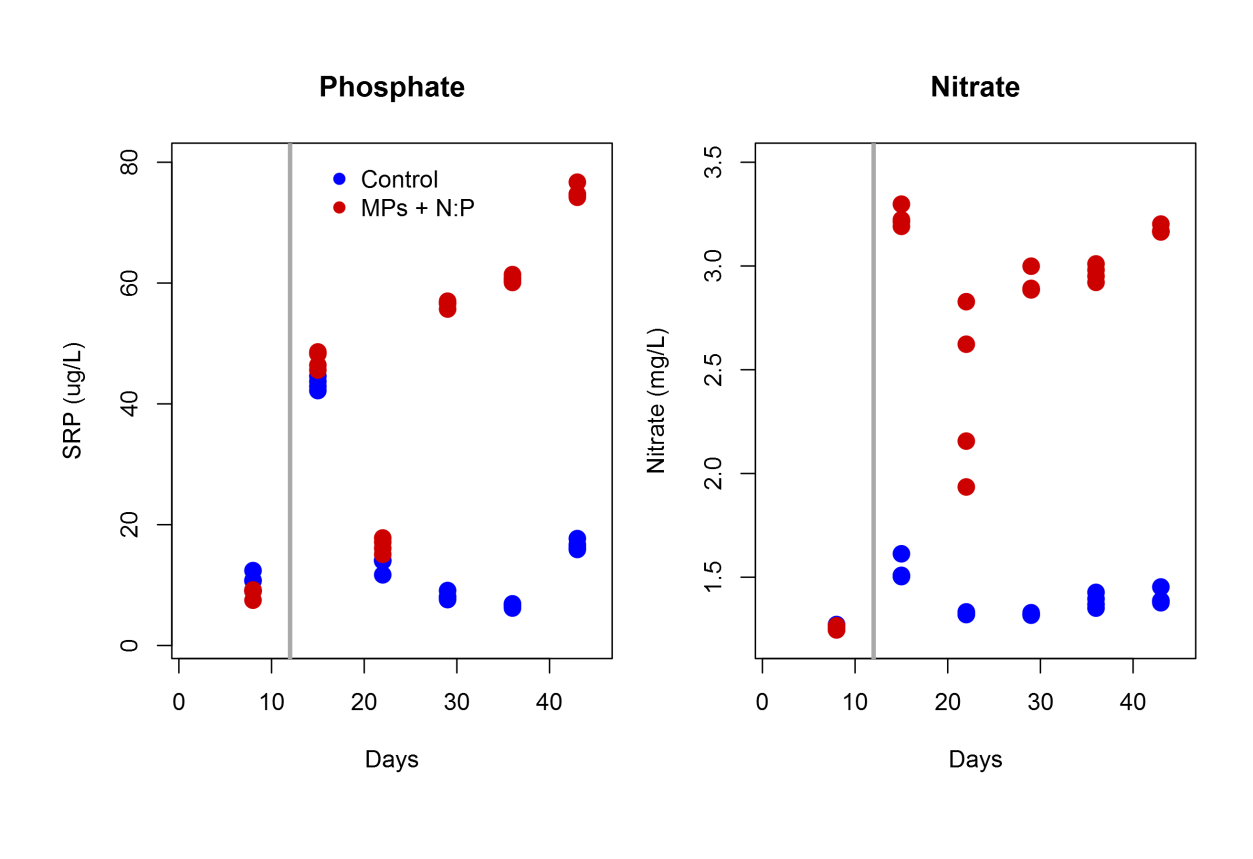


**Figure SD20** Nutrient levels in the control and the MP+N&P treatment during Exp.3. The grey vertical line indicates the start of dosing.


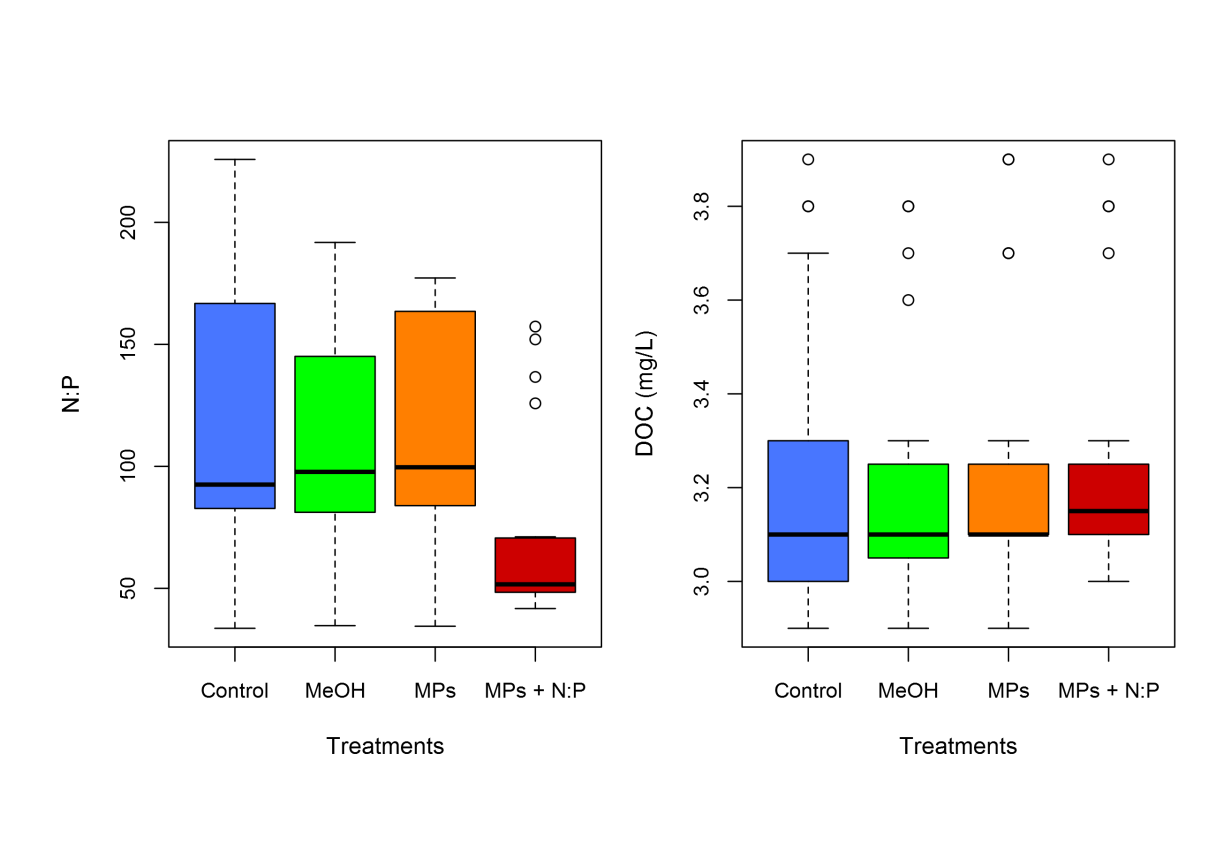


**Figure SD21** Comparison of N:P ratios and the DOC levels across the four treatments in Exp.3.

#### Micropollutants

The measurements indicate that the MP concentrations were generally higher in the MP as compared to the MP+N&P treatment. This was pronounced during Week 3 of the experiment (Day 23). The comparison among the different compounds that were dosed reveals a very consistent pattern such that the differences had a common cause (Figs.SD22–25).


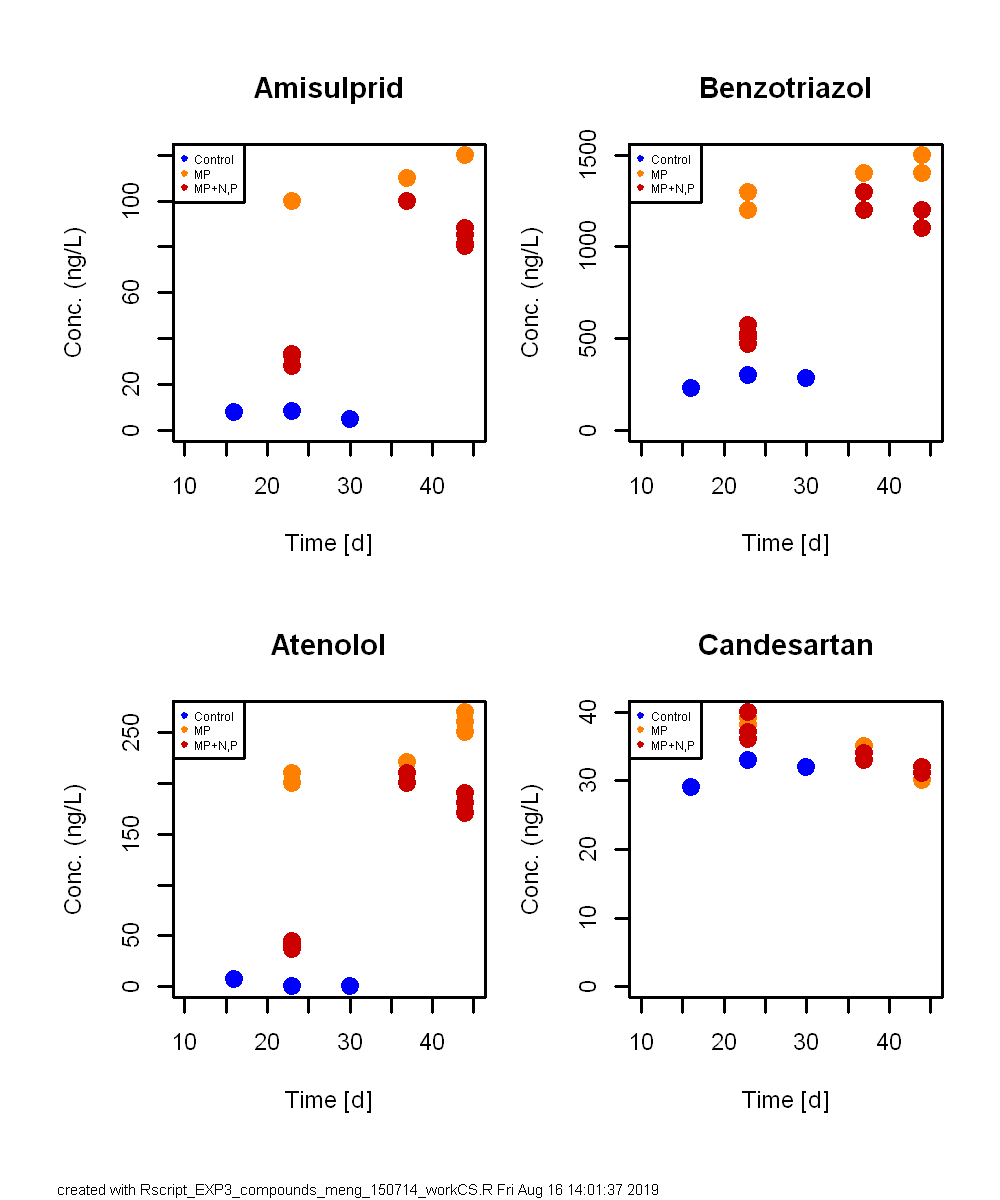


**Figure SD22** Set 1: Concentrations of micropollutants in the three Exp.3 treatments (Glatt control, blue; MPs, orange; MP+N&P, red).


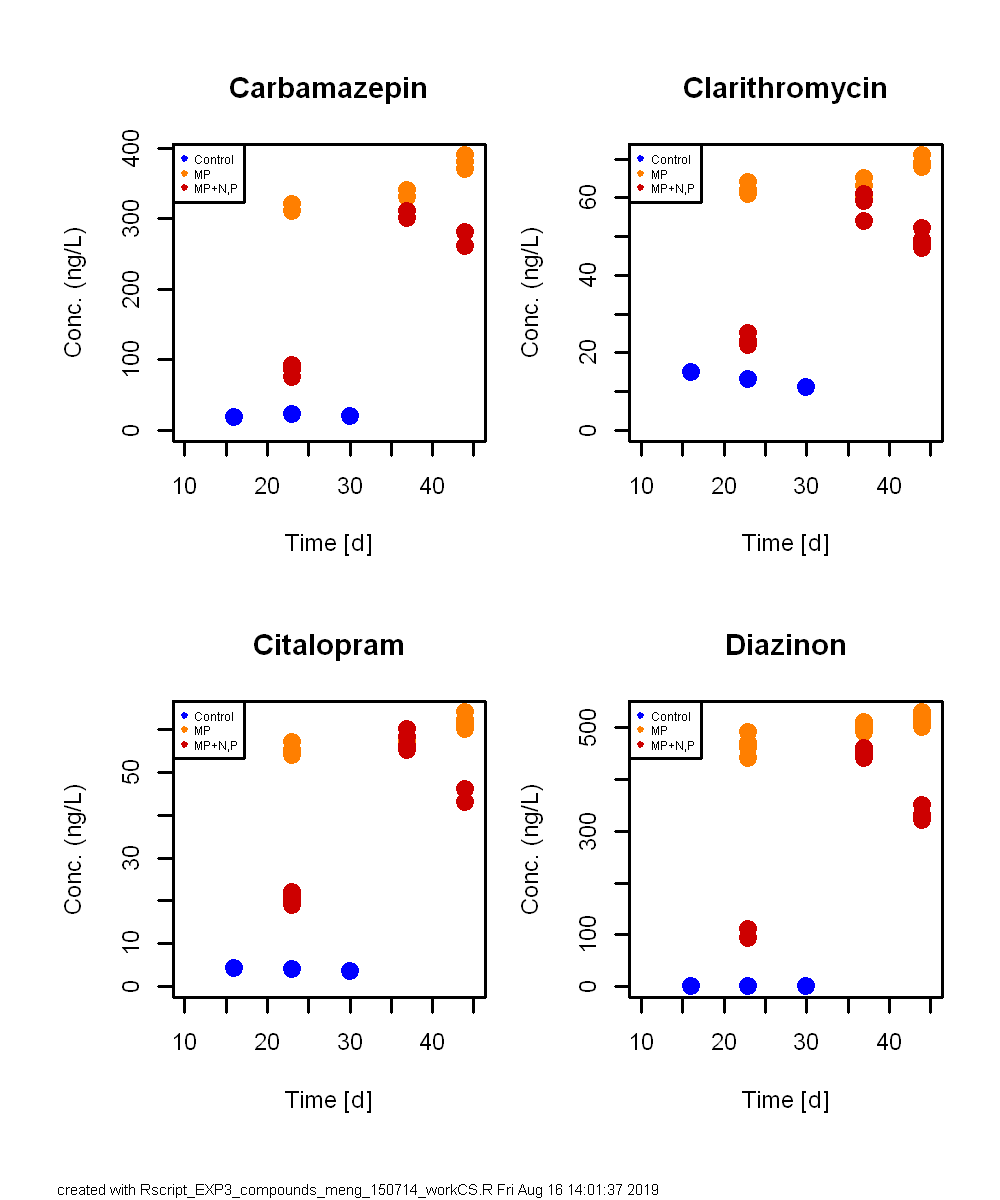


**Figure SD23** Set 2: Concentrations of micropollutants in the three Exp.3 treatments (Glatt control, blue; MPs, orange; MP+N&P, red).


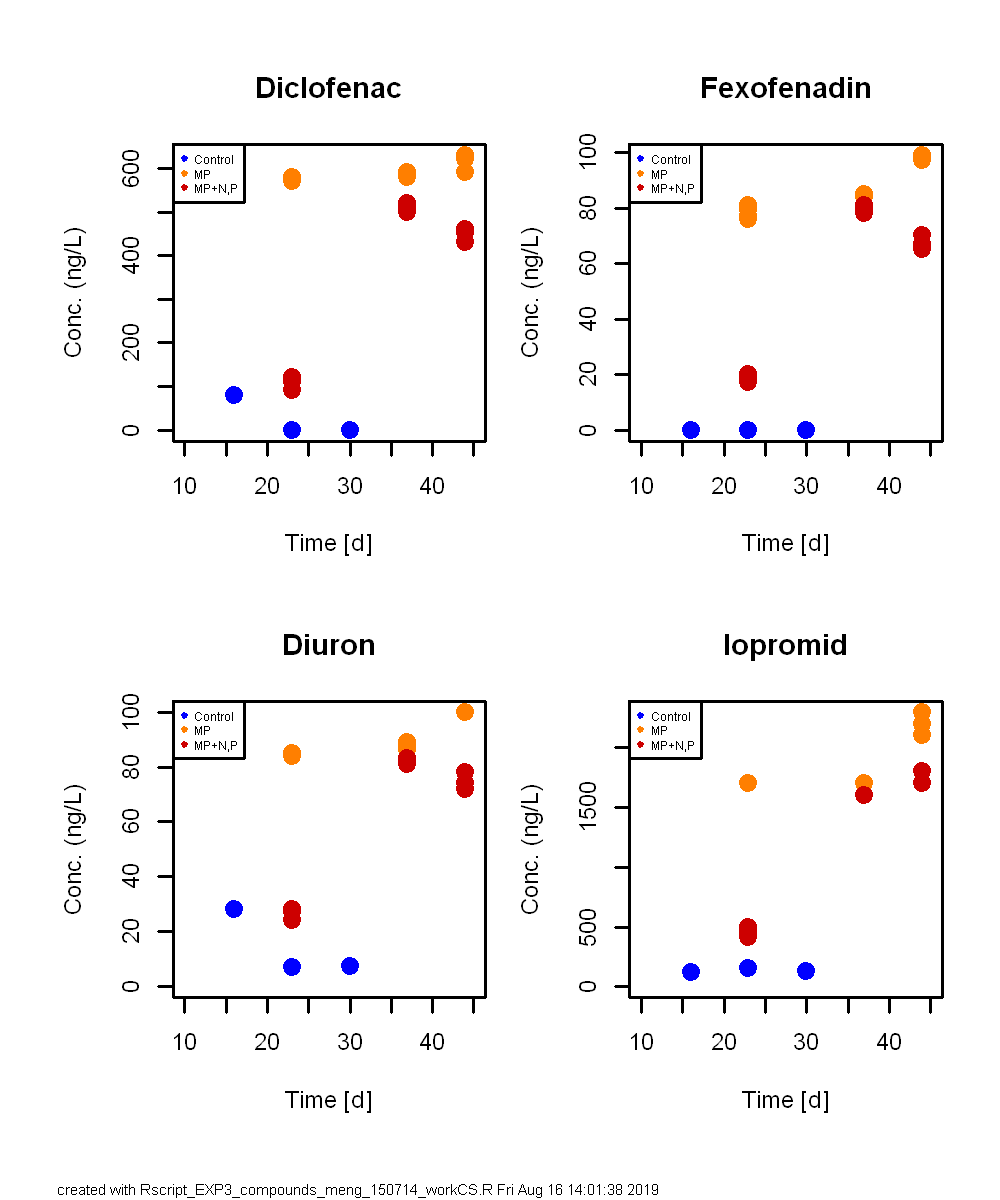


**Figure SD24** Set 3: Concentrations of micropollutants in the three Exp.3 treatments (Glatt control, blue; MPs, orange; MP+N&P, red).


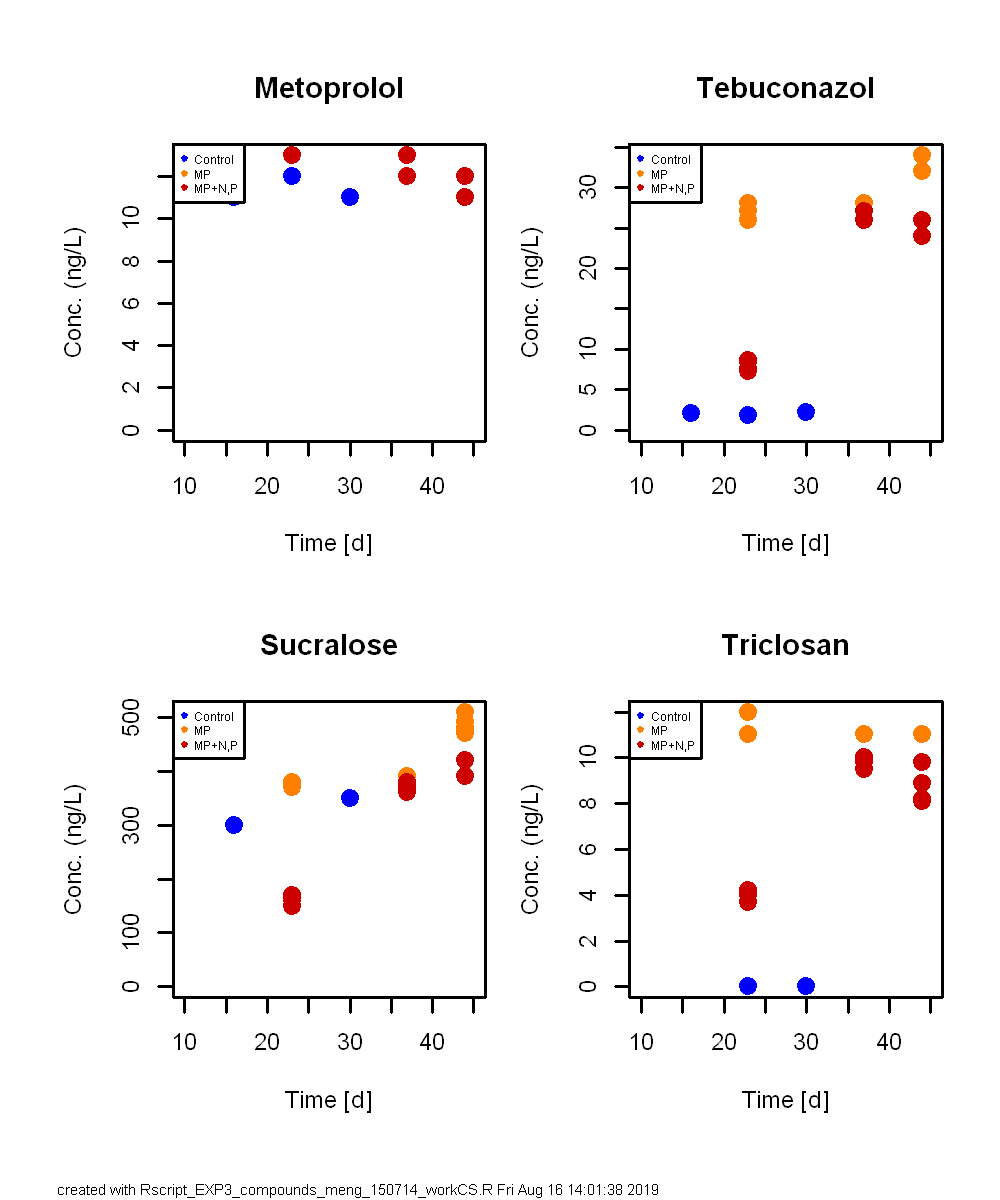


**Figure SD25** Set 4: Concentrations of micropollutants in the three Exp.3 treatments (Glatt control, blue; MPs, orange; MP+N&P, red).

### D.2.4 Experiment 4

#### Water quality

**Table SD12**. Observed nutrient levels in the two water quality treatments used in Exp.4.

| Treatment | Conductivity | Nitrate | SRP | DOC |
| --- | --- | --- | --- | --- |
|  | µS/cm 20°C | mg/L | µg/L | mg/L |
| Glatt (control) | 401 | 0.8 | 8.0 | 3.1 |
| 50% WW | 648 | 4.6 | 107 | 4.6 |

## D.3 Additional experimental results including mass-loss rates

### D.3.1 Comparison of CSA responses from Exp.2 & 3


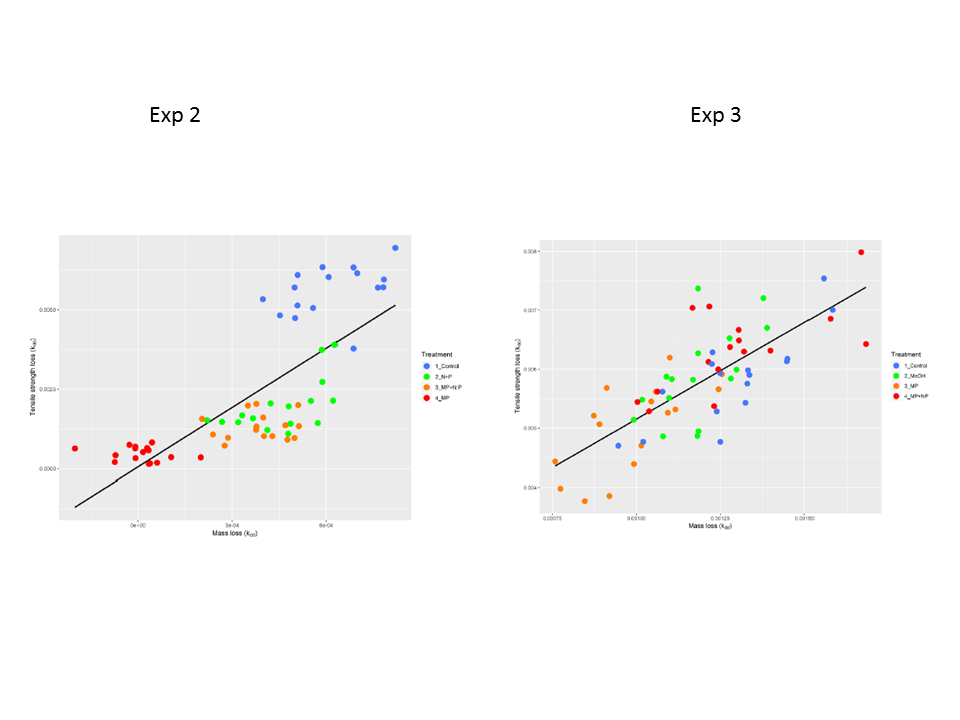


**Figure SD26** Relationships between rates of cotton mass loss and tensile-strength loss during Exp.2 (left) and Exp.3 (right).


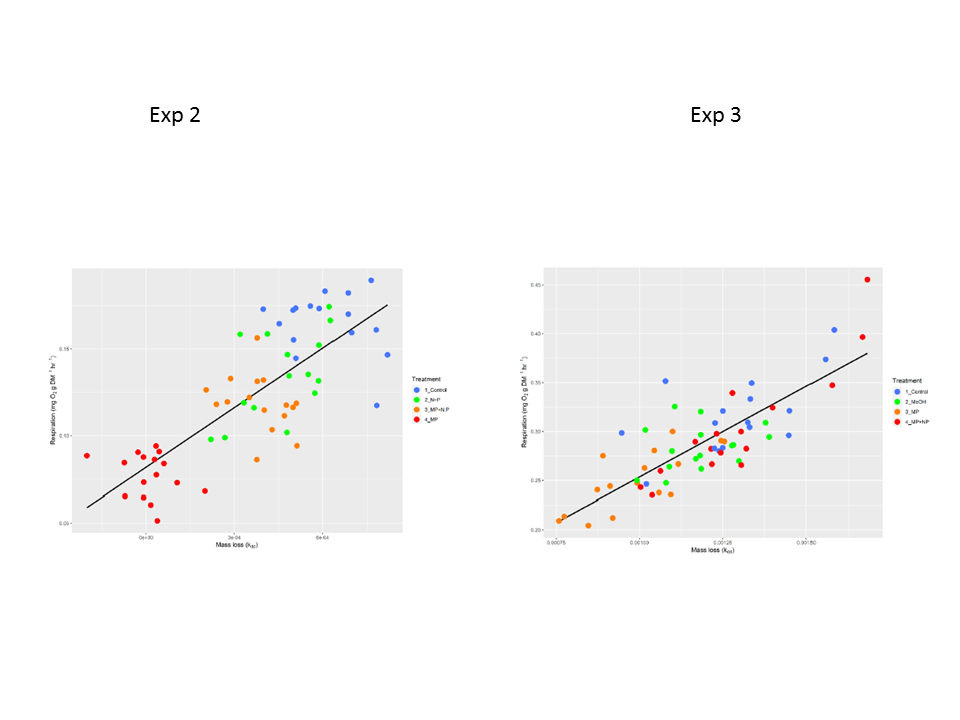


**Figure SD27** Relationships between rates of cotton mass loss and respiration during Exp.2 (left) and Exp.3 (right).


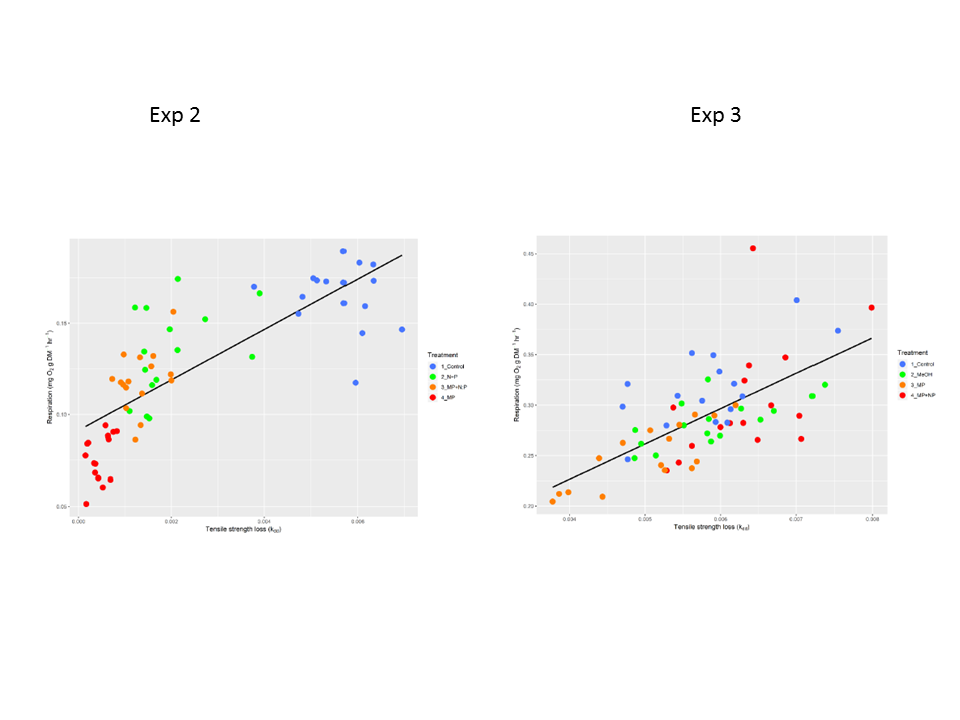


**Figure SD28** Relationships between rates of cotton tensile-strength loss and respiration during Exp.2 (left) and Exp.3 (right).

### D.3.2 Mass-loss rates from flumes experiments

**
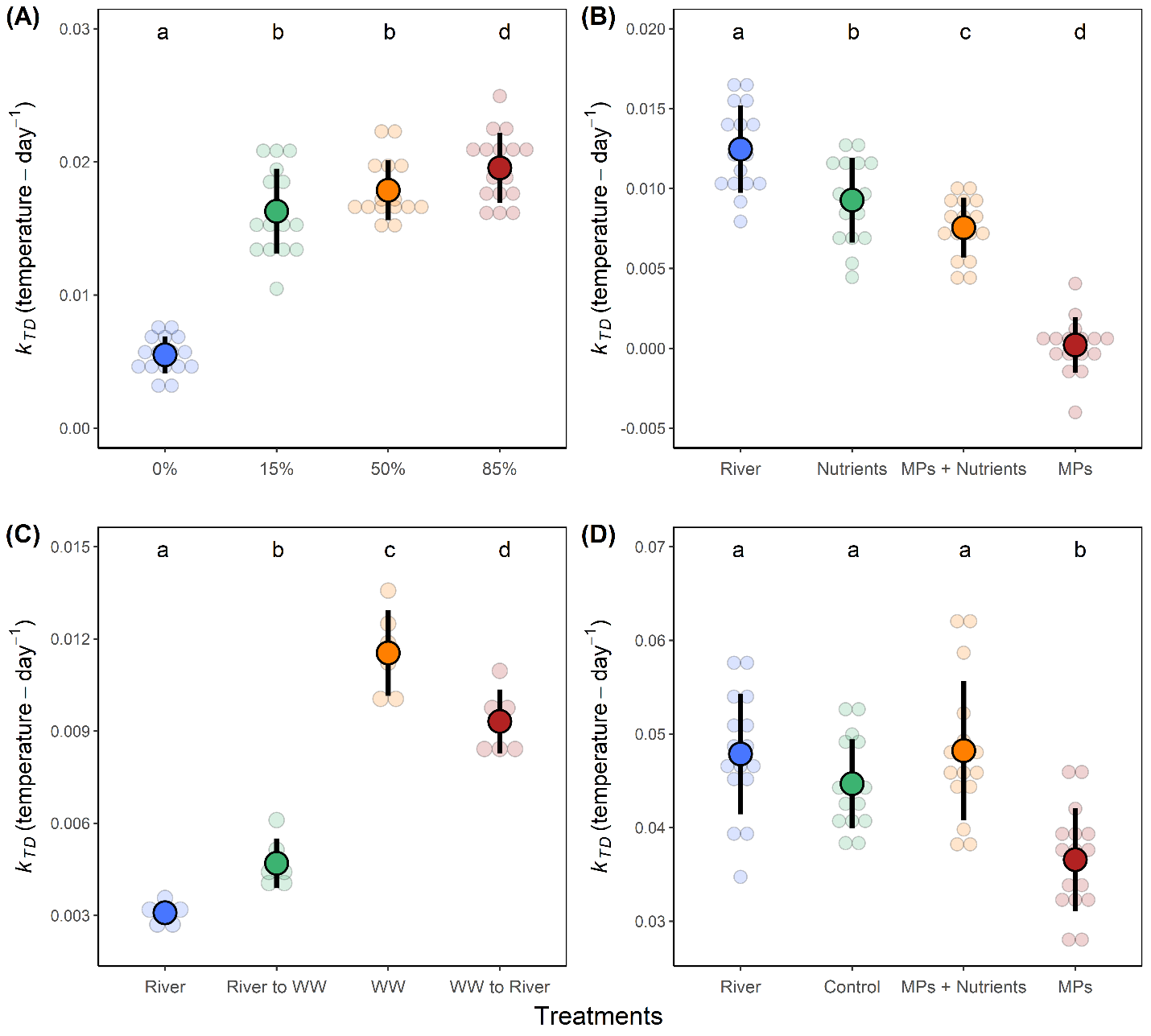
**

**Figure SD29** Mean (± 1 S.D) rates of cotton-strip mass loss from experiments in the Maiandros flumes system. From clockwise: A) the wastewater ‘Dilution’ experiment (Exp.1); B) the first ‘Dosing’ experiment (Exp.2); D) the second ‘Dosing’ experiment (Exp.3); and C) the inoculation experiment (Exp.4). For further details, see Table 1, Main text.

### D.3.3 Additional results from laboratory experiment

**
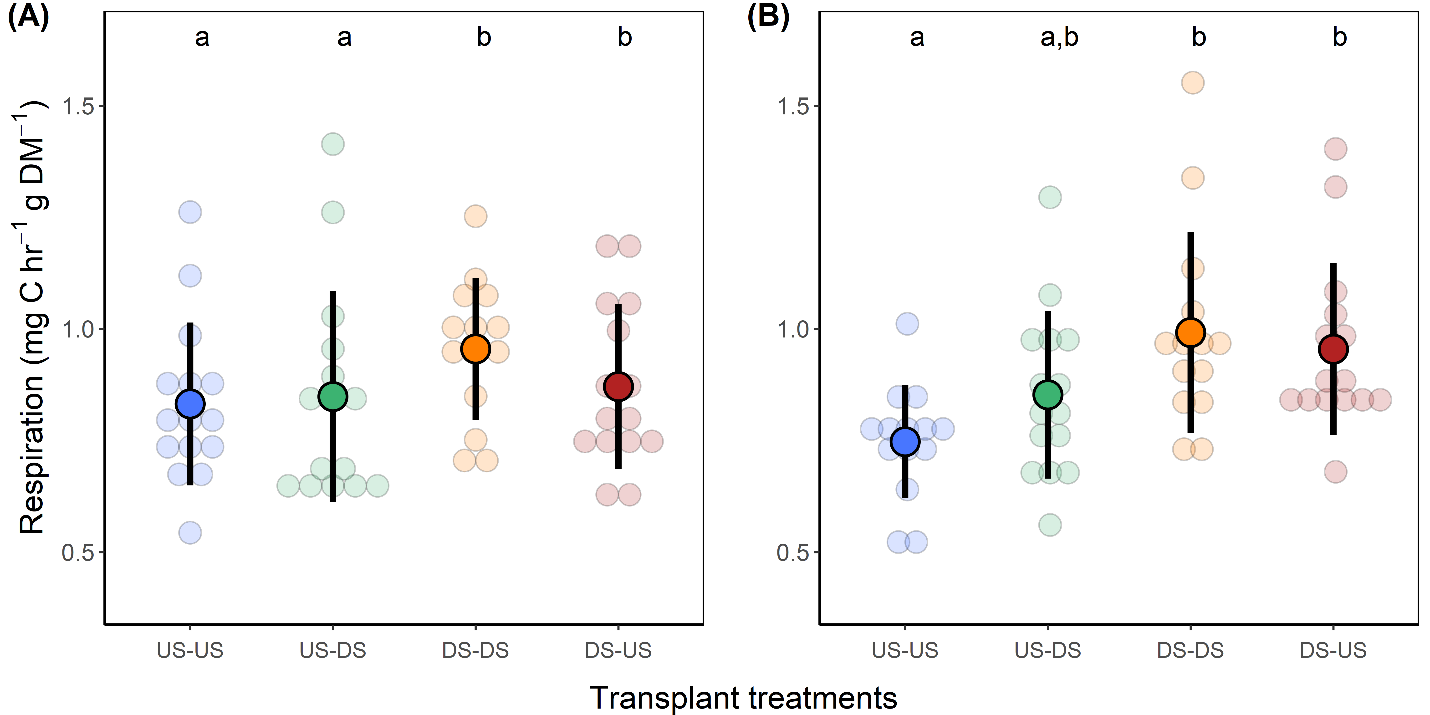
**

**Figure SD30** Mean (± 1 S.D) rates of cotton-strip respiration from the laboratory experiment with river water that was A) untreated; and B) filtered and sterilised. For further details, see Methods, Main text. This experiment involved cotton strips inoculated at three study sites with locations upstream (US) and downstream (DS) of WW inputs, then laboratory-incubated in water collected from the field (US/DS locations).

**
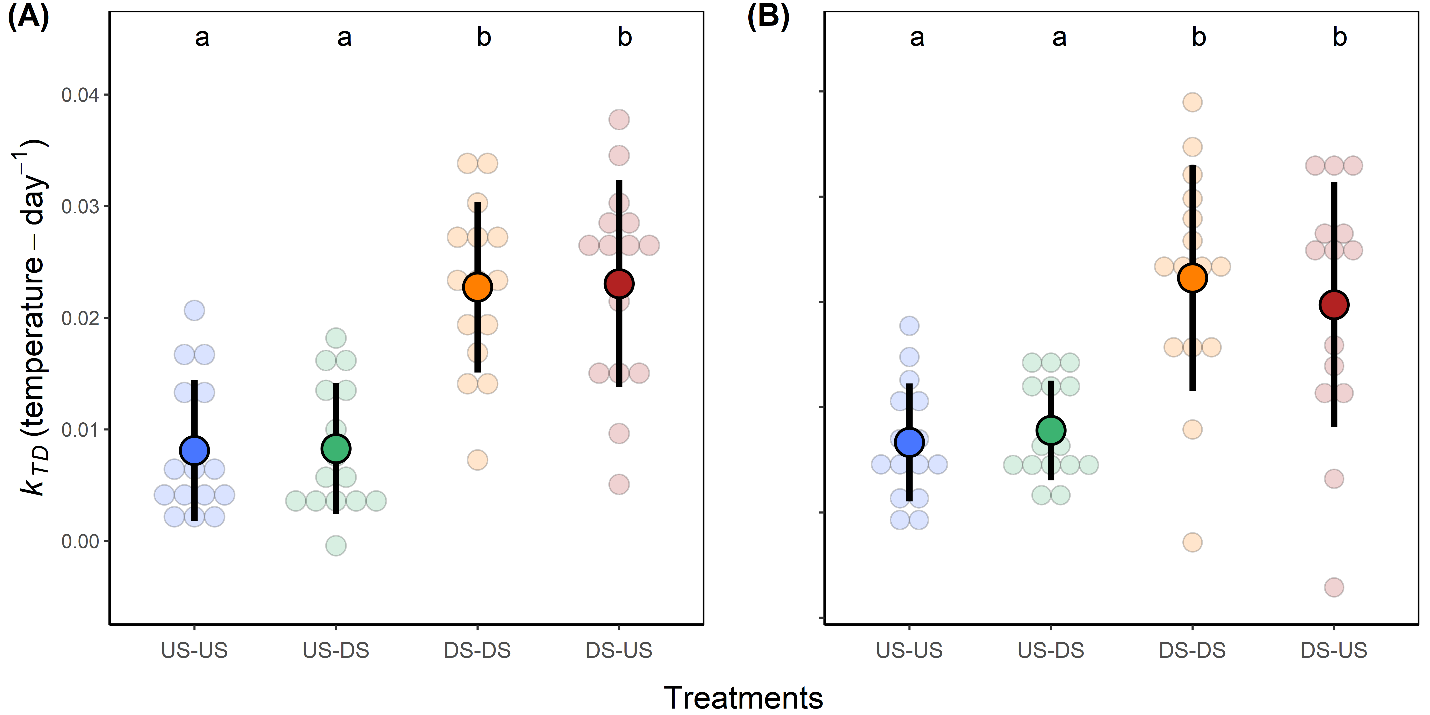
**

**Figure SD31** Mean (± 1 S.D) rates of cotton-strip tensile-strength loss from the laboratory experiment with river water that was A) untreated; and B) filtered and sterilised.

**
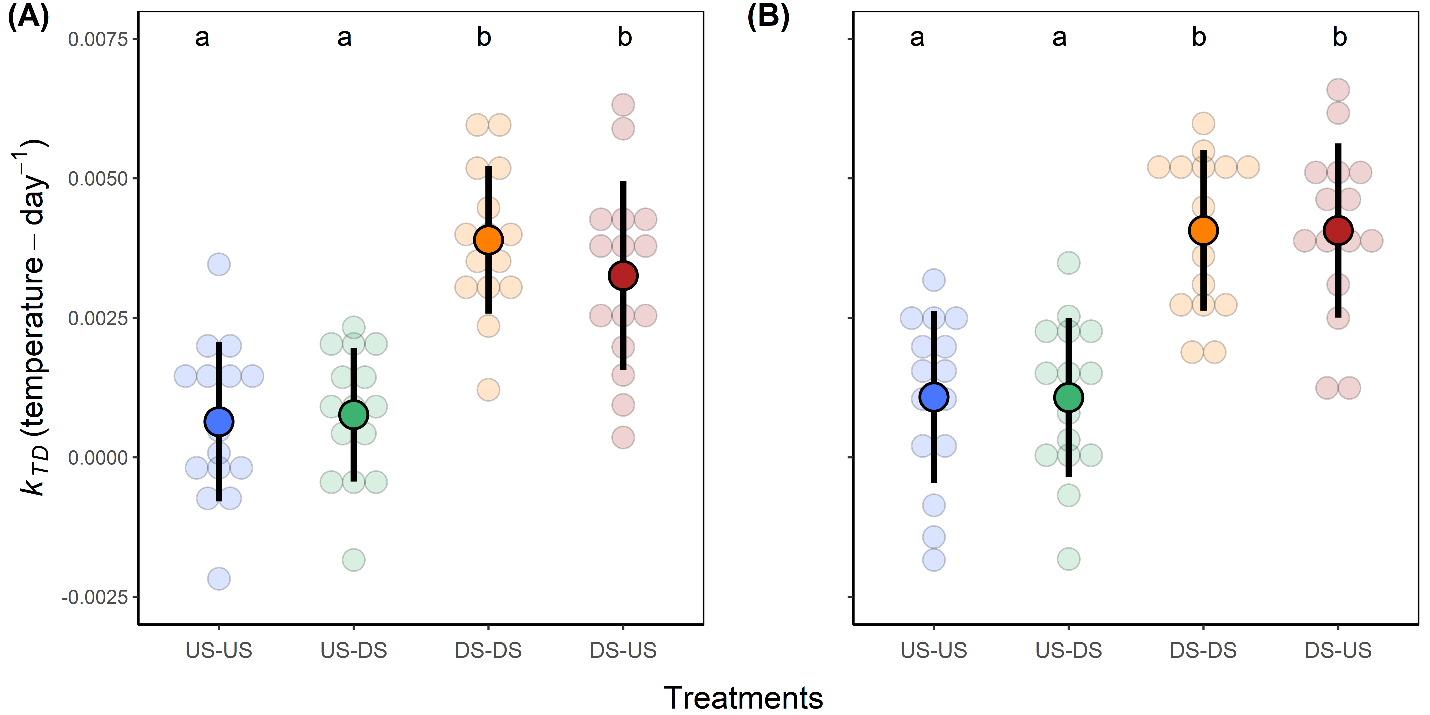
Figure SD32** Mean (± 1 S.D) rates of cotton-strip mass loss from the laboratory experiment with river water that was A) untreated; and B) filtered and sterilised.

**References**

Allen, G. H., and Pavelsky, T. M. (2018). Global extent of rivers and streams. Science 361:585-588. doi: 10.1126/science.aat0636

Berggren, M., Lapierre, J.-F., and del Giorgio, P. A. (2012). Magnitude and regulation of bacterioplankton respiratory quotient across freshwater environmental gradients. The ISME journal 6:984-993. doi: 10.1038/ismej.2011.157

Blanchet, F. G., Legendre, P., and Borcard, D. (2008). Forward selection of explanatory variables. Ecology 89:2623-2632. doi: 10.1890/07-0986.1

Blunden, J., and Arndt (eds), D. S. (2014). State of the Climate in 2013. Bulletin of the American Meteorological Society 95:S1–S257. doi: 10.1175/2014BAMSStateoftheClimate.1

Borcard, D., and Legendre, P. (2002). All-scale spatial analysis of ecological data by means of principal coordinates of neighbour matrices. Ecological Modelling 153:51-68. doi: 10.1016/S0304-3800(01)00501-4

Borcard, D., Legendre, P., Avois-Jacquet, C., and Tuomisto, H. (2004). Dissecting the spatial structure of ecological data at multiple scales. Ecology 85:1826-1832. doi: 10.1890/03-3111

Burdon, F. J., Munz, N. A., Reyes, M., Focks, A., Joss, A., Räsänen, K., Altermatt, F., Eggen, R. I. L., and Stamm, C. (2019). Agriculture versus wastewater pollution as drivers of macroinvertebrate community structure in streams. Science of The Total Environment 659:1256-1265. doi: 10.1016/j.scitotenv.2018.12.372

Burdon, F. J., Reyes, M., Alder, A. C., Joss, A., Ort, C., Räsänen, K., Jokela, J., Eggen, R. I. L., and Stamm, C. (2016). Environmental context and magnitude of disturbance influence trait-mediated community responses to wastewater in streams. Ecology and Evolution 6:3923–3939. doi: 10.1002/ece3.2165

Bürgmann, H., Jenni, S., Vazquez, F., and Udert, K. M. (2011). Regime Shift and Microbial Dynamics in a Sequencing Batch Reactor for Nitrification and Anammox Treatment of Urine. Applied and Environmental Microbiology 77:5897-5907. doi: 10.1128/aem.02986-10

Butman, D., and Raymond, P. A. (2011). Significant efflux of carbon dioxide from streams and rivers in the United States. Nature Geoscience 4:839-842. doi: 10.1038/ngeo1294

Canonica, S., Meunier, L., and von Gunten, U. (2008). Phototransformation of selected pharmaceuticals during UV treatment of drinking water. Water Research 42:121-128. doi: 10.1016/j.watres.2007.07.026

Chiu, C.-H., and Chao, A. (2016). Estimating and comparing microbial diversity in the presence of sequencing errors. PeerJ 4:e1634. doi: 10.7717/peerj.1634

Chung, Y., Rabe-Hesketh, S., Dorie, V., Gelman, A., and Liu, J. (2013). A nondegenerate penalized likelihood estimator for variance parameters in multilevel models. Psychometrika 78:685-709. doi: 10.1007/s11336-013-9328-2

Colas, F., Woodward, G., Burdon, F. J., Guérold, F., Chauvet, E., Cornut, J., Cébron, A., Clivot, H., Danger, M., Danner, M. C., Pagnout, C., and Tiegs, S. D. (2019). Towards a simple global-standard bioassay for a key ecosystem process: organic-matter decomposition using cotton-strip assays. Ecological Indicators 106:105466. doi: 10.1016/j.ecolind.2019.105466

Demars, B. O. L., and Manson, J. R. (2013). Temperature dependence of stream aeration coefficients and the effect of water turbulence: A critical review. Water Research 47:1-15. doi: 10.1016/j.watres.2012.09.054

Dray, S., Legendre, P., and Peres-Neto, P. R. (2006). Spatial modelling: a comprehensive framework for principal coordinate analysis of neighbour matrices (PCNM). Ecological Modelling 196:483-493. doi: 10.1016/j.ecolmodel.2006.02.015

Ferreira, V., and Chauvet, E. (2011). Synergistic effects of water temperature and dissolved nutrients on litter decomposition and associated fungi. Global Change Biology 17:551-564. doi: 10.1111/j.1365-2486.2010.02185.x

Frykman, P. K., Nordenskjöld, A., Kawaguchi, A., Hui, T. T., Granström, A. L., Cheng, Z., Tang, J., Underhill, D. M., Iliev, I., Funari, V. A., Wester, T., and Group, H. C. R. (2015). Characterization of Bacterial and Fungal Microbiome in Children with Hirschsprung Disease with and without a History of Enterocolitis: A Multicenter Study. PloS one 10:e0124172-e0124172. doi: 10.1371/journal.pone.0124172

Gulis, V., and Suberkropp, K. (2003). Interactions between stream fungi and bacteria associated with decomposing leaf litter at different levels of nutrient availability. Aquatic Microbial Ecology 30:149-157. doi: 10.1128/AEM.70.9.5266-5273.2004

Hsieh, T. C., Ma, K. H., and Chao, A. (2016). iNEXT: an R package for rarefaction and extrapolation of species diversity (Hill numbers). Methods in Ecology and Evolution 7:1451-1456. doi: 10.1111/2041-210x.12613

Jähne, B., Heinz, G., and Dietrich, W. (1987). Measurement of the diffusion coefficients of sparingly soluble gases in water. Journal of Geophysical Research: Oceans 92:10767-10776. doi: 10.1029/JC092iC10p10767

Kaviraj, A., Bhunia, F., and Saha, N. C. (2004). Toxicity of Methanol to Fish, Crustacean, Oligochaete Worm, and Aquatic Ecosystem. International Journal of Toxicology 23:55-63. doi: 10.1080/10915810490265469

Legendre, P. (2008). Studying beta diversity: ecological variation partitioning by multiple regression and canonical analysis. Journal of Plant Ecology 1:3-8. doi: 10.1093/jpe/rtm001

Legendre, P., and Gallagher, E. D. (2001). Ecologically meaningful transformations for ordination of species data. Oecologia 129:271-280. doi: 10.1007/s004420100716

Love, M. I., Huber, W., and Anders, S. (2014). Moderated estimation of fold change and dispersion for RNA-seq data with DESeq2. Genome Biology 15:550. doi: 10.1186/s13059-014-0550-8

Luke, S. G. (2017). Evaluating significance in linear mixed-effects models in R. Behaviour Research Methods 49:1494-1502. doi: 10.3758/s13428-016-0809-y

Maclntyre, S., Wanninkhof, R., and Chanton, J. P. (1995). Trace gas exchange across the air-water interface in freshwaters and coastal marine environments Pages 52-97 *in* P. A. Matson and R. C. Harriss, editors. Biogenic trace gases: Measuring emissions from soil and water. Wiley-Blackwell.

Mangold, S. (2015). From a real aquatic environment to an experimental approach – developing and monitoring stream mesocosm experiments to simulate a wastewater polluted stream. Diploma Thesis. Technical University Berlin in collaboration with Eawag, Zurich, Switzerland.

Meylan, S., Behra, R., and Sigg, L. (2003). Accumulation of copper and zinc in periphyton in response to dynamic variations of metal speciation in freshwater. Environmental Science & Technology 37:5204-5212. doi: 10.1021/es034566+

Munz, N., Leu, C., and Wittmer, I. (2012). Pestizidmessungen in Fliessgewässern - Schweizweite Auswertung. Aqua & Gas 92:32-41. doi: https://www.dora.lib4ri.ch/eawag/islandora/object/eawag:15423

Munz, N. A., Burdon, F. J., de Zwart, D., Junghans, M., Melo, L., Reyes, M., Schönenberger, U., Singer, H. P., Spycher, B., Hollender, J., and Stamm, C. (2016). Pesticides drive risk of micropollutants in wastewater-impacted streams during low flow conditions. Water Research 110:366-377. doi: 10.1016/j.watres.2016.11.001

Peres-Neto, P. R., Jackson, D. A., and Somers, K. M. (2003). Giving meaningful interpretation to ordination axes: assessing loading significance in principal component analysis. Ecology 84:2347-2363. doi: 10.2307/3450140

Perkins, D. M., Yvon-Durocher, G., Demars, B. O. L., Reiss, J., Pichler, D. E., Friberg, N., Trimmer, M., and Woodward, G. (2012). Consistent temperature dependence of respiration across ecosystems contrasting in thermal history. Global Change Biology 18:1300-1311. doi: 10.1111/j.1365-2486.2011.02597.x

Pomati, F., and Nizzetto, L. (2013). Assessing triclosan-induced ecological and trans-generational effects in natural phytoplankton communities: a trait-based field method. Ecotoxicology 22:779-794. doi: 10.1007/s10646-013-1068-7

Raymond, P. A., Caraco, N. F., and Cole, J. J. (1997). Carbon dioxide concentration and atmospheric flux in the Hudson River. Estuaries 20:381-390. doi: 10.2307/1352351

Raymond, P. A., Hartmann, J., Lauerwald, R., Sobek, S., McDonald, C., Hoover, M., Butman, D., Striegl, R., Mayorga, E., Humborg, C., Kortelainen, P., Dürr, H., Meybeck, M., Ciais, P., and Guth, P. (2013). Global carbon dioxide emissions from inland waters. Nature 503:355. doi: 10.1038/nature12760

Raymond, P. A., Zappa, C. J., Butman, D., Bott, T. L., Potter, J., Mulholland, P., Laursen, A. E., McDowell, W. H., and Newbold, D. (2012). Scaling the gas transfer velocity and hydraulic geometry in streams and small rivers. Limnology and Oceanography: Fluids and Environments 2:41-53. doi: 10.1215/21573689-1597669

Schielzeth, H. (2010). Simple means to improve the interpretability of regression coefficients. Methods in Ecology and Evolution 1:103-113. doi: 10.1111/j.2041-210X.2010.00012.x

Slocum, M. G., Roberts, J., and Mendelssohn, I. A. (2009). Artist canvas as a new standard for the cotton-strip assay. Journal of Plant Nutrition and Soil Science 172:71-74. doi: 10.1002/jpln.200800179

Sprague, J. B. (1971). Measurement of pollutant toxicity to fish—III. Water Research 5:245-266. doi: 10.1016/0043-1354(71)90171-0

Stamm, C., Räsänen, K., Burdon, F. J., Altermatt, F., Jokela, J., Joss, A., Ackermann, M., and Eggen, R. I. L. (2016). Unravelling the impacts of micropollutants in aquatic ecosystems: cross-disciplinary studies at the interface of large-scale ecology. Advances in Ecological Research 24:183 - 223. doi: 10.1016/bs.aecr.2016.07.002

Strahm, I., Munz, N., Leu, C., Wittmer, I., and Stamm, C. (2013). Landnutzung entlang des Gewässernetzes. Quellen für Mikroverunreinigungen. Aqua & Gas 93:36-44. doi: https://www.dora.lib4ri.ch/eawag/islandora/object/eawag:8951

Tiegs, S. D., Clapcott, J. E., Griffiths, N. A., and Boulton, A. J. (2013). A standardized cotton-strip assay for measuring organic-matter decomposition in streams. Ecological Indicators 32:131-139. doi: 10.1016/j.ecolind.2013.03.013

Tuan, N. N., Chang, Y.-C., Yu, C.-P., and Huang, S.-L. (2014). Multiple approaches to characterize the microbial community in a thermophilic anaerobic digester running on swine manure: A case study. Microbiological Research 169:717-724. doi: 10.1016/j.micres.2014.02.003

Wanninkhof, R. (1992). Relationship between wind speed and gas exchange over the ocean. Journal of Geophysical Research: Oceans 97:7373-7382. doi: 10.1029/92jc00188

Weiss, R. F. (1974). Carbon dioxide in water and seawater: the solubility of a non-ideal gas. Marine Chemistry 2:203-215. doi: 10.1016/0304-4203(74)90015-2

Yannarell, A. C., Busby, R. R., Denight, M. L., Gebhart, D. L., and Taylor, S. J. (2011). Soil bacteria and fungi respond on different spatial scales to invasion by the legume *Lespedeza cuneata*. Front Microbiol 2:127. doi: 10.3389/fmicb.2011.00127

Yannarell, A. C., Kent, A. D., Lauster, G. H., Kratz, T. K., and Triplett, E. W. (2003). Temporal patterns in bacterial communities in three temperate lakes of different trophic status. Microbial Ecology 46:391-405. doi: 10.1007/s00248-003-1008-9
